# Hsp70/J-protein machinery from *Glossina morsitans morsitans*, vector of African trypanosomiasis

**DOI:** 10.1371/journal.pone.0183858

**Published:** 2017-09-13

**Authors:** Stephen J. Bentley, Aileen Boshoff

**Affiliations:** Biotechnology Innovation Centre, Rhodes University, Grahamstown, South Africa; California State University Fullerton, UNITED STATES

## Abstract

Tsetse flies (*Glossina* spp.) are the sole vectors of the protozoan parasites of the genus *Trypanosoma*, the causative agents of African Trypanosomiasis. Species of *Glossina* differ in vector competence and *Glossina morsitans morsitans* is associated with transmission of *Trypanosoma brucei rhodesiense*, which causes an acute and often fatal form of African Trypanosomiasis. Heat shock proteins are evolutionarily conserved proteins that play critical roles in proteostasis. The activity of heat shock protein 70 (Hsp70) is regulated by interactions with its J-protein (Hsp40) co-chaperones. Inhibition of these interactions are emerging as potential therapeutic targets. The assembly and annotation of the *G*. *m*. *morsitans* genome provided a platform to identify and characterize the Hsp70s and J-proteins, and carry out an evolutionary comparison to its well-studied eukaryotic counterparts, *Drosophila melanogaster* and *Homo sapiens*, as well as *Stomoxys calcitrans*, a comparator species. In our study, we identified 9 putative Hsp70 proteins and 37 putative J-proteins in *G*. *m*. *morsitans*. Phylogenetic analyses revealed three evolutionarily distinct groups of Hsp70s, with a closer relationship to orthologues from its blood-feeding dipteran relative *Stomoxys calcitrans*. *G*. *m*. *morsitans* also lacked the high number of heat inducible Hsp70s found in *D*. *melanogaster*. The potential localisations, functions, domain organisations and Hsp70/J-protein partnerships were also identified. A greater understanding of the heat shock 70 (Hsp70) and J-protein (Hsp40) families in *G*. *m*. *morsitans* could enhance our understanding of the cell biology of the tsetse fly.

## Introduction

African trypanosomiasis is a parasitic disease giving rise to infection in both humans and animals. Human African trypanosomiasis (HAT) is a neglected tropical disease that burdens 37 countries in sub-Saharan Africa, with an estimated population of 70 million at risk of contracting this potentially lethal disease [1]. Animal African trypanosomiasis (AAT), also known as *Nagana*, afflicts both wild animals and domesticated livestock and has a detrimental impact on the economic development within sub-Saharan Africa as rearing livestock is nearly impossible in endemic areas [2]. The etiological agent of African trypanosomiasis belongs to the genus *Trypanosoma*, an extracellularly blood- and tissue-borne unicellular parasitic protozoan. The parasite is comprised of three subspecies: *Trypanosoma brucei*, *Trypanosoma brucei gambiense* and *Trypanosoma brucei rhodesiense*, with the latter two being human-infective [3] and all three-subspecies having the potential to be vectors for AAT. *T*. *b*. *gambiense* gives rise to a chronic infection with symptoms that may be dormant for months and even years and represents over 90% of reported cases [4]. *T*. *b*. *rhodesiense* is mainly a zoonotic disease responsible for less than 10% of reported cases and causes an acute infection, which is rapidly fatal if untreated [5].

The tsetse fly, which belongs to the *Glossinidae* family, which is comprised of only the *Glossina* genus [6], is the sole insect vector for all the *Trypanosoma spp*. residing in sub-Saharan Africa [7]. The trypanosomes are transmitted to its mammalian host when an infected tsetse fly vector takes a blood meal, which ensures the cyclical transmission of the parasite between hosts [1]. Thirty-three species and subspecies of tsetse flies have been identified [8], and classified into three subgenera: the *Palpalis* group, the *Morsitans* group and the *Fusca* group [9–10]. Host specificity of these groups differs, with the *Palpalis* group associating with humans and human activities, while the *Morsitans* and *Fusca* groups are associated with wild animals and cattle [11]. Flies of the *Morsitans* group prefer savannah and woodland habitats and are found mainly in East Africa and might be involved in the transmission of *T*. *b*. *rhodesiense* [12].

Tools for controlling the neglected tropical disease are limited, due to the inability to develop a vaccine, slow development of new and effective drugs, and the ever-increasing drug resistance in African trypanosomes to the current drug treatment regiments [13]. Strategies to control the vector have gained prominence in recent years and vector control could be improved by genome analysis [14–15]. An International *Glossina* Genome Initiative was established in 2004 to expand research capacity in sub-Saharan Africa, with the goal of sequencing a *Glossina* species [15]. This goal was realised in 2014 with the release of the *Glossina morsitans morsitans* genome which has enabled exploration of the cell biology of the insect vector, essentially aiding in the search for alternative strategies in controlling African trypanosomiasis [16]. Part of the original International *Glossina* Genome Initiative also included the sequencing of the non-vector obligate blood feeder *Stomoxys calcitrans*, also known as the stable fly [16–17]. Knowledge is lacking on growth and differentiation of trypanosomes in the tsetse fly, as well as vector-parasite interactions [18]. Heat shock proteins and the complexes that they form have gained significant interest as potential drug targets for a variety of diseases [19].

Heat shock proteins (Hsps) play a prominent role in protein biosynthesis, and maintaining homeostasis within the cell under both normal and stressful conditions [20]. Hsps are either constitutively expressed (heat shock cognates, Hsc), and maintain cellular homeostasis, or are up-regulated in response to external stimuli (heat shock proteins, Hsp) [21]. Hsps are traditionally classified according to their molecular weight (kDa), although an alternative nomenclature has been proposed for the major human Hsp families [22]. Members of the Hsp70 superfamily, comprising of the Hsp70/HSPA family and the Hsp110/HSPH family, are the most highly conserved heat shock protein family due to the indispensable role played in maintaining cellular homeostasis, as well as a host of other cellular processes [23]. The Hsp70 and J-proteins function together to bind polypeptides in a variety of essential cellular processes, including folding and unfolding of polypeptides, protein translocation and degradation [24]. Hsp70 proteins function in all major subcellular compartments of the cell, including the cytosol, nucleus, endoplasmic reticulum (ER), and the mitochondria. The number of J-proteins typically exceeds the number of Hsp70s in the cell, and as a result multiple J-proteins can interact with a single Hsp70, which enhances the functional diversity of Hsp70s [25]. The Hsp110s are divergent members of the Hsp70 superfamily and belong to one of the four classes of nucleotide exchange factors of the eukaryotic Hsp70 cycle that accelerate ADP-ATP exchange [26]. A few Hsp110s are able to bind substrate and prevent aggregation by functioning as “holdases” as the interaction cannot be modulated [27]. In addition, Hsp110s have been shown to play a prominent role in the protein disaggregation and reactivation machinery [28–29].

The Hsp70-based chaperone machineries are ATP-dependent processes that involve repetitive cycles of peptide binding and release that are facilitated by ATP binding and hydrolysis [30]. J-proteins play a crucial function of stimulating the basal ATPase activity of Hsp70 partners, while nucleotide exchange factors facilitate the exchange of ADP for ATP resulting in a conformational change in the substrate binding domain and bound substrates are released as the affinity of Hsp70 for its client protein is reduced [31]. The ~ 70 amino acid signature region known as the J-domain possesses an invariant His-Pro-Asp (HPD) motif that has been shown to play a vital role in stimulating the ATPase activity [32]. J-proteins are generally grouped into four classes based on their structural homology to the *E*. *coli* DnaJ [33–34]. While all J-proteins contain the canonical J-domain, most have additional domains that perform a variety of functions, including binding client proteins for subsequent transfer to Hsp70, targeting J-proteins to a particular cellular location or obtaining further factors necessary for their function [35].

The aim of this study was to analyse both the Hsp70 and J-protein complements found in the *G*. *m*. *morsitans* genome. Many Hsp70 proteins from *Drosophila* spp. have been characterised and *D*. *melanogaster hsp70* genes are often used as a reference for comparative genome studies in other organisms [36–37]. Dipteran insects often display an evolutionary proliferation of their *hsp70* genes and the Hsp70s from *D*. *melanogaster* include multiple constitutively expressed proteins (Hsc) and heat-inducible heat shock proteins (Hsp) [38]. This paper provides a comprehensive depiction of the Hsp70 and J-protein family from *G*. *m*. *morsitans* based on structural, functional and evolutionary analyses. *In silico* tools were used to evaluate the domain conservation, predicted subcellular localisation, syntenic and phylogenetic analysis of the Hsp70 and J-protein complements within *G*. *m*. *morsitans*. The Hsp70 and J-protein complements were also comparatively analysed in relation to those found in *D*. *melanogaster*, *H*. *sapiens*, and *S*. *calcitrans*, with the aim of identifying all Hsp70 and J-protein members, and potentially identifying Hsp70-J-protein partnerships. It is envisioned that the results of this study will provide a future context for studying the biology of the tsetse fly.

## Methodology

### Database mining and sequence analyses

In order to identify the Hsp70 complement of *G*. *m*. *morsitans*, the full set of Hsp70 genes from *D*. *melanogaster* were retrieved from FlyBase v6 (http://flybase.org/; [39]), and submitted as queries in a BLASTP search of the *G*. *m*. *morsitans* genome on the VectorBase (https://www.vectorbase.org; [17]) database. The e-value was set at an intermediately stringent level of e-10 for collecting as many potential hsp70-related sequences for further analysis. Keywords were also used to scan the genome of *G*. *m*. *morsitans* for *hsp70* genes on the VectorBase database, and these included “Hsp70”, “Heat shock protein”, and “molecular chaperone”. The retrieved amino acid sequences were then screened for the Hsp70 domain using SMART 7 (Simple Modular Architecture Research Tool; http://smart.embl-heidelberg.de/; [40]), and Prosite (http://prosite.expasy.org/; [41]).

Retrieval of the protein sequences for the J-protein complement of *G*. *m*. *morsitans* was conducted in the same manner, except the J-domain (1-77aa) of *Escherichia coli* DnaJ (EcDnaJ) was used as the query, as the signature region for all J-proteins is the J-domain [33], and J-proteins are divided into the four type classes based on their structural homology to *Escherichia coli* DnaJ [33–34]. The keywords: “Hsp40”, “DnaJ”, “Heat shock protein”, and “molecular chaperone” were also used to scan the genome of *G*. *m*. *morsitans* for *J-protein* genes on the VectorBase database. The retrieved amino acid sequences from the various keyword searches were then screened for the J-domain using SMART 7 (Simple Modular Architecture Research Tool; http://smart.embl-heidelberg.de/; [40]), and Prosite (http://prosite.expasy.org/; [41]). The molecular weight (Da) of each gene were calculated using compute pI/Mw tool from ExPASy [42].

### Phylogenetic and conserved syntenic analyses

Phylogenetic trees were constructed to analyse the phylogenetic relationship of the HSPA/Hsp70, HSPH/Hsp110, and J-protein complements of *G*. *m*. *morsitans*, *Stomoxys calcitrans* (*S*. *calcitrans*; stable fly), *Homo sapiens* (*H*. *sapiens*; humans), and *Drosophila melanogaster* (*D*. *melanogaster*; fruit fly). Separate phylogenetic trees for HSPA/Hsp70 and HSPH/Hsp110 were constructed, as the two Hsp70 subfamilies are very divergent. The Type III J-protein subfamily was omitted from the phylogenetic analysis, as the subfamily is diverse with regards to amino acid composition and protein length, with the only common feature being the J-domain. The full length amino acid sequences for the Hsp70 superfamily and selected J-protein subfamilies in the tsetse fly and stable fly were obtained from VectorBase [17], fruit fly protein sequences were obtained from FlyBase v6 [38], and human protein sequences were obtained from the National Centre for Biotechnology Information (NCBI) website (www.ncbi.nlm.nih.gov). Multiple sequence alignments were performed using the in-built ClustalW program [43] with default parameters in MEGA 7.0 [44], and are provided in the supplementary data, S1–S3 Figs. Maximum-likelihood was utilized to find the best model of evolution, and based on the Bayesian Information Criteria (BIC) the substitution pattern that was best described for the protein families was the Le Gascuel (LG) model matrix [45] with a discrete Gamma (G) distribution to model evolutionary rates amongst sites (Hsp70/HSPA, gamma value = 1.1925; Hsp110/HSPH, gamma value = 1.3525; J-protein, gamma value = 2.3202). Maximum likelihood phylogenetic trees were constructed using MEGA 7.0 [45]. The accuracy of the reconstructed trees was assessed using a bootstrap test using a 1000 replicates with a pairwise gap deletion mode. The phylogenetic trees for Hsp70/HSPA and Hsp110/HSPH were rooted with the *Escherichia coli* HscC (EcHscC) sequence. The phylogenetic tree for the J-proteins was unrooted.

In order to provide additional evidence for orthology, conserved syntenic regions surrounding selected Hsp70 genes were searched by examining the conserved co-localization of neighbouring genes on a scaffold of *G*. *m*. *morsitans* and the selected organisms for this study using genome information from VectorBase, FlyBase, and NCBI database. The identities of unknown neighbour genes of the selected Hsp70 genes were conducted using a BLASTP search on the NCBI database.

### Protein domain mapping, subcellular localisation and determination of fruit fly, stable fly and human orthologues

The protein domain mapping for the Hsp70 and J-protein complements from *G*. *m*. *morsitans* was conducted using a combination of online programs that included TPRpred (http://toolkit.tuebingen.mpg.de/tprpred; [46]), SMART 7 (Simple Modular Architecture Research Tool; http://smart.embl-heidelberg.de/; [40]), and Prosite (http://prosite.expasy.org/; [41]). The organelle distribution for the Hsp70 and J-protein complements were conducted, in the absence of experimental data, using a number of online programs that included NucPred (http://www.sbc.su.se/~maccallr/nucpred/cgi-bin/single.cgi; [47]), MitoPROT (http://ihg.gsf.de/ihg/mitoprot.html; [48]), MultiLoc (http://abi.inf.uni-tuebingen.de/Services/MultiLoc; [49]), SignalP version 4.1 (http://www.cbs.dtu.dk/services/SignalP/; [50]), and WoLF PSORT (http://www.genscript.com/wolf-psort.html.; [51]).

Aside from phylogenetic and syntenic analysis, identification of orthologues for the *G*. *m*. *morsitans* Hsp70 and J-protein genes in stable fly (*Stomoxys calcitrans*), humans (*Homo sapiens*), and the fruit fly (*Drosophila melanogaster*) were also conducted using reciprocal BLASTP. In the first query, the putative amino acid sequences of the 9 Hsp70 and 37 J-proteins of *G*. *m*. *morsitans* were used as queries in a BLASTP search on the National Centre for Biotechnology Information (NCBI) website (www.ncbi.nlm.nih.gov), using the default parameters. The amino acid sequences of the putative orthologues were then used as second queries in BLASTP searches using default parameters on the VectorBase database. If the most similar orthologue in *G*. *m*. *morsitans* was exactly the Hsp70 or J-protein sequence used as the first query, the sequence of the second query was selected as an orthologue.

## Results and discussion

### The Hsp70 complement of *G*. *m*. *morsitans*

As *G*. *m*. *morsitans* (referred to as Gmm in this study) and *D*. *melanogaster* (referred to as Dmel in this study) are both dipteran insects, the already well characterised Hsp70 proteins from *D*. *melanogaster* were used as queries and as a reference to explore the Hsp70 superfamily from *G*. *m*. *morsitans*, which has not been previously analysed. The nomenclature of the *G*. *m*. *morsitans* Hsp70s from VectorBase were derived from the nomenclature used for *D*. *melanogaster*, though, the nomenclature proposed in this study for the members of the Hsp110/HSPH family were based on their sequence similarity to their *Drosophila* and human orthologues. The nomenclature for the Hsp70 superfamily from the stable fly, *Stomoxys calcitrans* (referred to as Scal in this study) was derived in the same manner. A total of 9 putative Hsp70 genes, listed in Table 1, were identified in *G*. *m*. *morsitans*, with 3 of these belonging to the Hsp70 subfamily, Hsp110/HSPH. The domain architecture of the members of the GmmHsp70 and GmmHsp110 families are shown in S4 Fig.

**Table 1:** The predicted Hsp70 proteins from *G*. *m*. *morsitans* with their respective *D*. *melanogaster*, *S*. *calcitrans*, *and H*. *sapiens* orthologues.

*G*. *m*. *morsitans*		*D*. *melanogaster*		*S*. *calcitrans*		*H*. *sapiens*	
Gene ID^a^	Name^b^	Gene ID^a^	Name	Gene ID^a^	Name	Name	Localisation^c^
**A: Hsp70s**							
GMOY009493	GmmHsp70A	FBgn0013275	Hsp70Aa	SCAU008520	Hsp70	-	CYT/NUC
GMOY009495	GmmHsp68	FBgn0001230	Hsp68	SCAU003728	Hsp68	-	CYT/NUC
GMOY004286	GmmHsc70-1	FBgn0001216	Hsc70-1	SCAU005225	Hsc70-1	-	CYT
GMOY003216	GmmHsc70-3	FBgn0001218	Hsc70-3	SCAU000678	Hsc70-3	HSPA5	ER
GMOY012049	GmmHsc70-4	FBgn0266599	Hsc70-4	SCAU015347	Hsc70-4	HSPA8	CYT
GMOY010851	GmmHsc70-5	FBgn0001220	Hsc70-5	SCAU003620	Hsc70-5	HSPA9	MITO
**B: NEFs**							
GMOY011246	GmmHsp110-1	FBgn0026418	Hsp110	SCAU005995	Hsp110	HSPH1	CYT
GMOY013289	GmmHsp110-2	-	-	-	-	-	CYT
GMOY006943	GmmGrp170	FBgn0023529	Grp170	SCAU010922	Grp170	HSPH4	ER

^**a**^ The Gene IDs for the *G*. *m*. *morsitans*, *S*. *calcitrans* and *D*. *melanogaster* Hsp70 proteins were acquired from the VectorBase database (https://www.vectorbase.org/; [17]), and FlyBase v6 database (http://flybase.org/; [39]) respectively.

^**b**^ The nomenclature for the Hsp70 proteins from *G*. *m*. *morsitans* was derived from the VectorBase database (https://www.vectorbase.org/; 38]); the proposed names in this study for the *G*. *m morsitans* NEFs were derived from their orthologues in *D*. *melanogaster*.

^**c**^ The subcellular localisations for the *G*. *m*. *morsitans* and *S*. *calcitrans* Hsp70 proteins were predicted using various online prediction servers, which are listed in the methods and *D*. *melanogaster* and *H*. *sapiens* have been experimentally determined (see text for details).

CYT-Cytosol; MITO- Mitochondria; ER- Endoplasmic reticulum; NUC-nucleus.

All retrieved amino acid sequences from VectorBase were full-length except for GmmHsp68 (GMOY009495). Analysis of the coding region of GmmHsp68 indicated that the protein was truncated to 619 amino acids due to a premature stop codon, and an additional 15 amino acids were found in the flanking 3’ downstream sequence that ended in a variant C-terminal EEID motif. Though the isoleucine substitution in the C-terminal EEVD motif could be a result of a sequencing error as the amino acids share similar side chains, but further sequence validation is needed to identify if this is indeed a miss-annotation. The amino acid sequence of GmmHsp110-2 (GMOY010029) was also re-annotated as it was found to possess all the functional domains of a typical Hsp110 protein but also possessed unusually an alkyl hydro peroxide reductase subunit C (AhpC) at the C-terminus of the protein. Insertion of a stop codon at the C-terminus of the protein prior to the AhpC yields a full-length protein but further sequence validation is needed to identify if this is indeed a miss-annotation.

**Fig 1:**
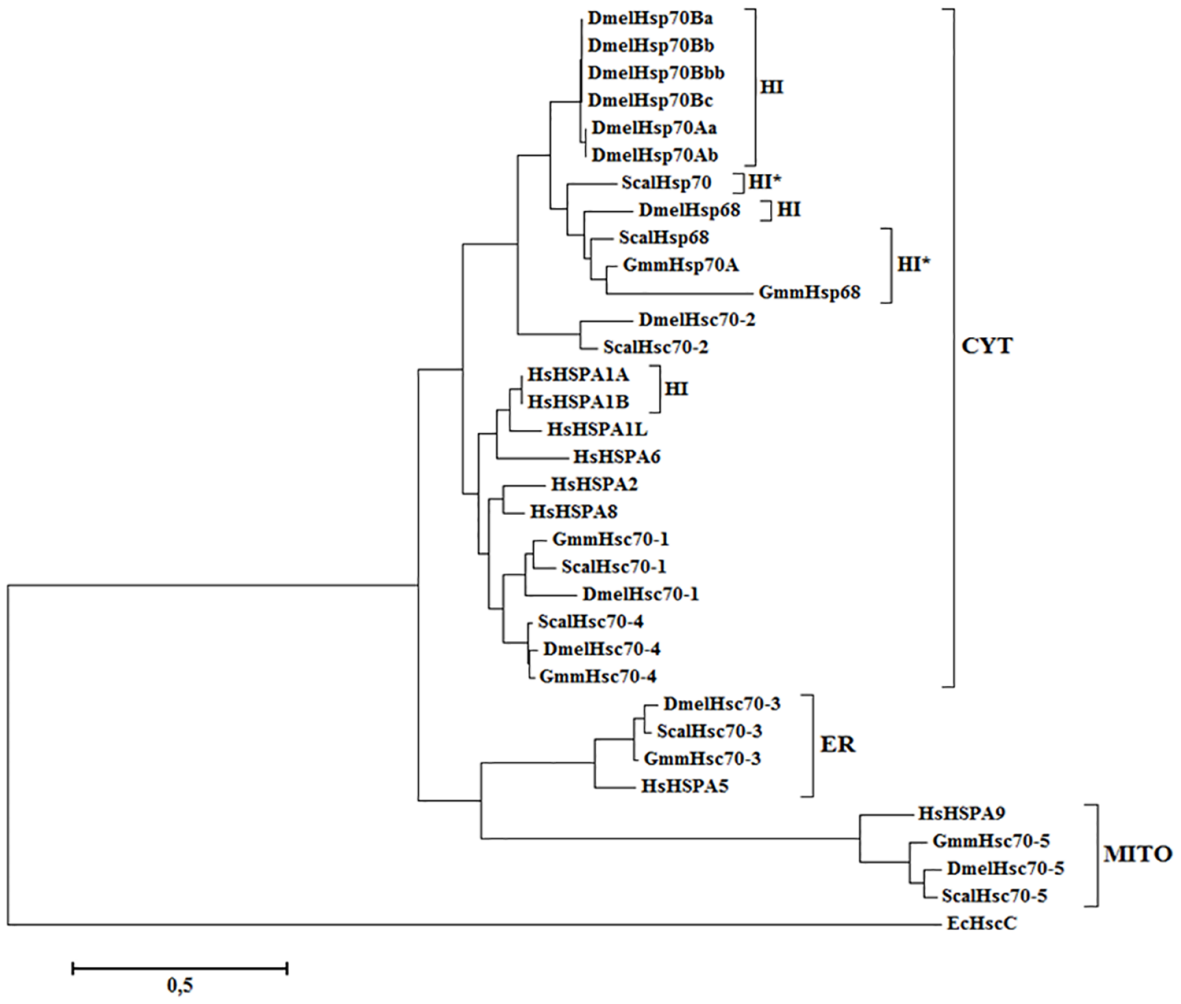
Phylogenetic analysis of the predicted Hsp70/HSPA family from *G*. *m*. *morsitans* in relation to *D*. *melanogaster*, *H*. *sapiens and S*. *calcitrans*. Multiple sequence alignment of the full-length amino acid sequences of the Hsp70/HSPA gene families in humans, tsetse flies, fruit flies, and stable flies. The multiple sequence alignment (S1 Fig) was performed using the in-built ClustalW program [43] with default parameters on the MEGA7 software [44]. The phylogenetic tree was constructed by MEGA 7 using the Maximum-likelihood method based on the Le Gascuel (LG) matrix-based model of amino acid substitution. A discrete Gamma distribution was used to model evolutionarily rate differences among sites (2 categories (+G, parameter = 1.1925). The alignment gaps were excluded from the analysis, and the number of amino acid sites used to construct the tree numbered 457. Bootstrap analysis was computed with 1000 replicates. Accession numbers of the sequences used: *E*. *coli*: HscC (NP_415183.1). *S*. *calcitrans*: Hsp70 (SCAU008520); Hsp68 (SCAU003728); Hsc70-1 (SCAU005225); Hsc70-2 (SCAU008036); Hsc70-3 (SCAU000678); Hsc70-4 (SCAU015347); Hsc70-5 (SCAU003620). *D*. *melanogaster*: Hsp68 (NP_524474.1); Hsp70Aa (NP_731651.1); Hsp70Ab (NP_524798.2); Hsp70Ba (NP_731716.1); Hsp70Bb (NP_524927.2); Hsp70Bbb (NP_788663.1); Hsp70Bc (NP_650209.1); Hsc70-1 (NP_524063.1); Hsc70-2 (NP_524339.1); Hsc70-3 (NP_727563.1); Hsc70-4 (NP_524356.1); Hsc70-5 (NP_523741.2). *H*. *sapiens*: HSPA1A (NP_005336.3); HSPA1B (NP_005337.2); HSPA1L (NP_005518.3); HSPA2 (NP_068814.2); HSPA5 (NP_005338.1); HSPA6 (NP_002146.2); HSPA8 (NP_006588.1); HSPA9 (NP_004125.3). Accession numbers for the *G*. *m*. *morsitans* Hsp70 sequences can be found in Table 1. The subcellular localisation for Hsp70s is indicated by a bracket on the right-hand side. CYT: cytosolic; ER: endoplasmic reticulum; MITO: mitochondrion. Hsp70 genes that are heat-inducible are depicted with HI. Hsp70 genes in *G*. *m*. *morsitans* that are predicted to be heat inducible are depicted with HI*.

The predicted subcellular localisations and the orthologous relationships of the Hsp70 and Hsp110 proteins from *G*. *m*. *morsitans* to the selected organisms in this study, as determined by pBLAST analysis (S1 Table) are presented in Table 1. Hsp70s are one of the most conserved groups of proteins [52–54], and thus, it was not surprising that the members of the Hsp70/HSPA family of *G*. *m*. *morsitans* showed a high degree of sequence identity to its orthologues in humans, and the selected dipteran species in this study (S1 Table). Though, there were no human orthologues of GmmHsp70A, GmmHsp68 or GmmHsc70-1 (Fig 1, Table 1). Notably absent from the Hsp70/HSPA family in *G*. *m*. *morsitans* is the six highly conserved copies of the inducible *Hsp70* gene (*Hsp70Aa*, *Hsp70Ab*, *Hsp70Ba*, *Hsp70Bb*, *Hsp70Bbb*, and *Hsp70Bc*) that are found in *D*. *melanogaster*. The inducible *Hsp70* gene in *D*. *melanogaster* has gone through extensive duplication during evolution [55], as this system has been specialized for intense expression during heat shock [56]. DmelHsp70 is virtually undetectable at normal growing temperatures of 25°C and is rapidly induced during heat shock where it plays an essential role in thermotolerance [57]. The absence of these inducible *Hsp70* genes could imply that the duplication event did not occur in *G*. *m*. *morsitans*.

Hsp68 proteins are very closely related to the Hsp70 proteins. Interestingly, the sequence similarity of GmmHsp68 was 70% identical to DmelHsp70Aa and 69% identical to DmelHsp68 (S1 Table). DmelHsp68 has been shown to partially compensate for the loss of Hsp70 in *Hsp70*-deficient flies, as the *Hsp68* expression increased in the absence of Hsp70 [55]. Recently, DmelHsp68 has been shown to assist Hsp70-null larvae in cold acclimation when exposed to relatively mild doses of cold [58]. DmelHsp68 has also been implicated as a component in JNK-signalling where this gene regulatory network utilizes the chaperone in limiting oxidative damage, and thus extending the lifespan of the fly [59]. Both GmmHsp70A and GmmHsp68 were predicted to localise in the nucleus and cytosol (Table 1). Heat shock has shown to cause a concentration of DmelHsp70 in the nuclei, with some remaining in the cytosol, and during recovery the protein returns to the cytosol [60].

The GmmHsc70s were predicted to localise to the same subcellular compartments as their *D*. *melanogaster* and *S*. *calcitrans* orthologues, however no Hsc70-2 orthologue was found for *G*. *m*. *morsitans* (Table 1). GmmHsc70-3 was predicted to be localised to the ER as a hydrophobic leader sequence and C-terminal KDEL motif, characteristic of ER proteins, was reported, and a mitochondrial leader sequence detected for the larger Hsc70-5 protein (S1 and S4 Figs). The Hsc70 family from *D*. *melanogaster* carries out critical functions at normal temperatures as mutations in several of these proteins caused lethality [61]. Transcription of *D*. *melanogaster Hsc70* genes are regulated during development, Hsc70-4 was present at high levels during embryonic, larval, and adult developmental stages, while Hsc70-1 and Hsc70-2 were detected in adults at low levels [62]. Proteomic profiling in *D*. *melanogaster* revealed that Hsc70-3, Hsc70-4 and Hsc70-5 as well as Hsp70Bb were significantly up-regulated during thermal acclimation [63]. The localisations of the human Hsp70 orthologues have been experimentally determined and corresponded to those of *G*. *m*. *morsitans* and the other selected dipteran species [64].

The phylogenetic tree in Fig 1 shows the classification of Hsp70 genes into three major monophyletic groups based on sub-cellular localisation (CYT, ER, MITO) among the different eukaryotes in this study, with heat inducible Hsp70s highlighted in the CYT subfamily. Functional differences of the three groups is reinforced by the phylogenetic analysis. The six inducible DmelHsp70 proteins phylogenetically clustered together, while DmelHsp68 appeared to be more closely related to the Hsp70 and Hsp68 proteins from *G*. *m*. *morsitans* and *S*. *calcitrans* (Fig 1). The Gmm cytosolic, mitochondrial and ER Hsp70s phylogenetically clustered with the dipteran Hsp70s, suggesting that they may be more functionally similar to the fruit fly and stable fly than to their human Hsp70 orthologues (Fig 1). Based on phylogenetic analysis both GmmHsp68 and GmmHsp70A are probably heat inducible, although the presence of heat shock elements needs to be confirmed. Not surprisingly the GmmHsc70 proteins clustered with their respective orthologues from *D*. *melanogaster* and *S*. *calcitrans* (Fig 1). DmelHsc70-2 and ScalHsc70-2 forms a unique clade and appears to be derived from the inducible Hsp70s, although an orthologue is absent from *G*. *m*. *morsitans* as well as *H*. *sapiens* (Fig 1). Based on phylogenetic analysis, the constitutive GmmHsc70 proteins appear to follow the same model of divergent evolution evident in *D*. *melanogaster* (Fig 1) [65]. Thus, it is possible that the Hsc70 proteins from *G*. *m*. *morsitans* and *S*. *calcitrans* could play a similar functional role to those from *Drosophila*.

**Fig 2:**
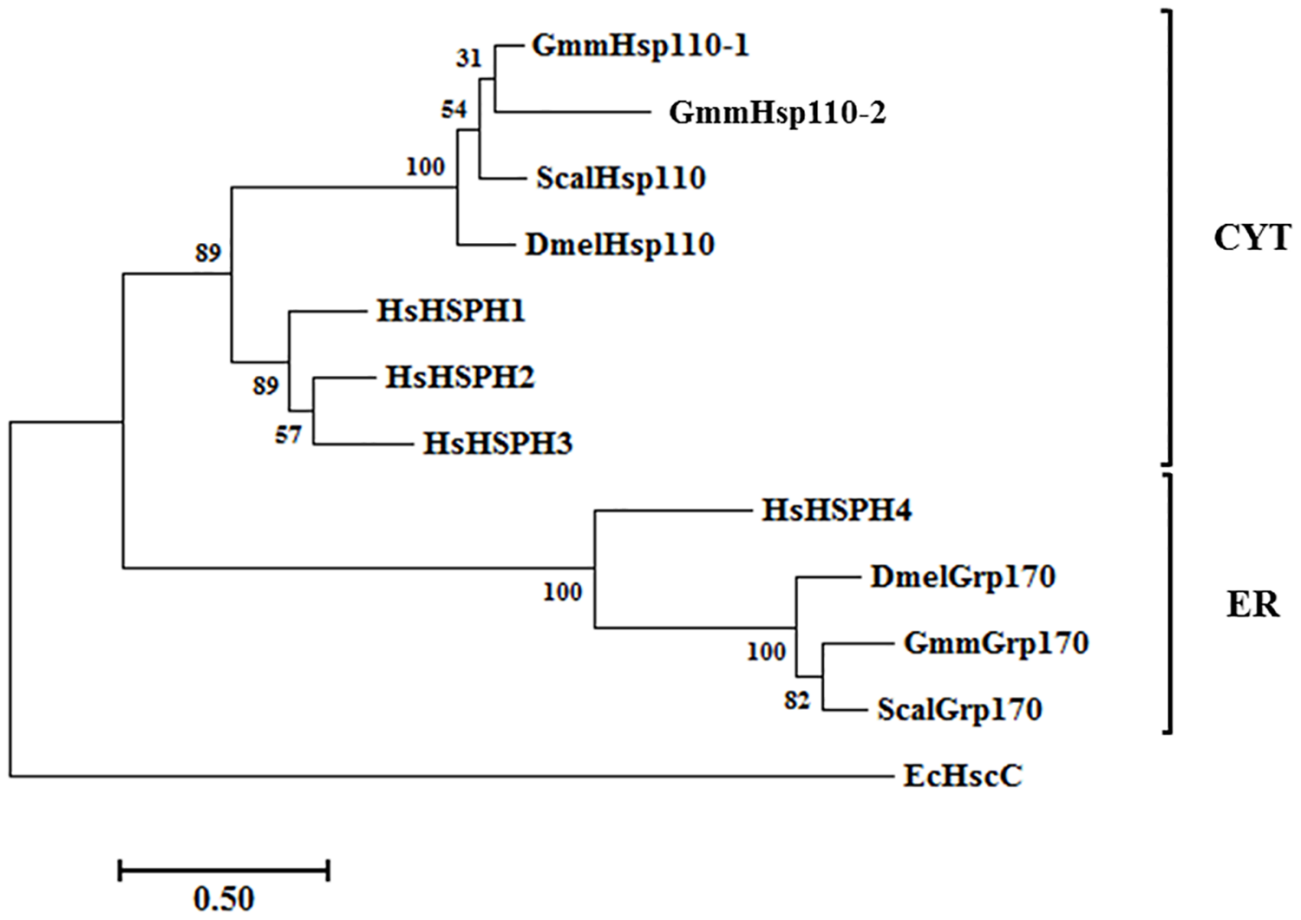
Phylogenetic relationship of the Hsp110/HSPH protein family from *G*. *m*. *morsitans* (Gmm), *D*. *melanogaster* (Dmel), *S*. *calcitrans* (Scal), and *H*. *sapiens* (Hs). Multiple sequence alignment of the full-length amino acid sequences of the Hsp110/HSPH gene families in humans, tsetse flies, fruit flies, and stable flies. The multiple sequence alignment (S2 Fig) was performed using the in-built ClustalW program [43] with default parameters on the MEGA7 software [44]. The phylogenetic tree was constructed by MEGA 7 using the Maximum-likelihood method based on the Le Gascuel (LG) matrix-based model of amino acid substitution. A discrete Gamma distribution was used to model evolutionarily rate differences among sites (2 categories (+G, parameter = 1.3525). The alignment gaps were excluded from the analysis, and the number of amino acid sites used to construct the tree numbered 544. Bootstrap analysis was computed with 1000 replicates. Accession numbers of the sequences used: *E*. *coli*: HscC (NP_415183.1). *S*. *calcitrans*: Hsp110 (SCAU005995); Grp170 (SCAU010922). D. melanogaster: Hsp110 (NP_648687.1); Grp170 (NP_569995.1). *H*. *sapiens*: HSPH1 (NP_006635.2); HSPH2 (NP_002145.3); HSPH3 (NP_055093.2); HSPH4 (NP_006380.1). Accession numbers for the *G*. *m*. *morsitans* Hsp110 sequences can be found in Table 1. The subcellular localisation for Hsp110s is indicated by a bracket on the right. CYT: cytosolic; ER: endoplasmic reticulum.

Two GmmNEFs were predicted to reside in the cytosol, whilst no Hsp110 orthologue was found in the mitochondria (Table 1, S4 Fig). This is not surprising as the mitochondrial GmmNEF is probably GmmRoe1 (GMOY010619), an orthologue of Mge1 which belongs to the GrpE class of NEFs for eukaryotic Hsp70s [66]. According to FlyBase, eight splice variants exist for Dmel Hsc70Cb/Hsp110, and further analysis of the NEFs revealed that GmmHsp110-1 (GMOY011246) exhibited the highest sequence identities to Hsc70Cb isoforms G and H (68.2%), while Hsp110-2 protein from *G*. *m*. *morsitans* (GMOY010029) exhibited the highest sequence identities to DmelHsc70Cb isoforms A, B, C, E, F and I (58.4%). The Hsp110s can be classified into the polyphyletic CYT group and the monophyletic ER group. Unlike *D*. *melanogaster* and *S*. *calcitrans*, *G*. *m*. *morsitans* has evolved three Hsp110 proteins, and the expression of additional isoforms cannot be ruled out (Fig 2). The human genome encodes three Hsp110 homologues that reside in the cytosol and one Grp170 homologue in the ER (Fig 2) [reviewed by 25]. A single Hsp110 homologue was found in the ER for all species (Fig 2).

GmmHsp110-1, GmmHsp110-2 and GmmGrp170 were found to be considerably longer in sequence length than the other identified Hsp70 members (S4 Fig). These are features typical of the Hsp110/HSPH family. Hsp110 and Grp170 have similar domains as canonical Hsp70s but have long insertions and C-terminal extensions [26]. DmelHsp110 was shown in a genome-wide RNAi screen to be a mitigating factor for aggregation of Huntington proteins [67]. The ATPase domain and C-terminal helical lid of Hsp110 have been shown to mediate the interaction with Hsp70 [27]. The putative peptide binding domain of Hsp110 is also unique with regards to the molecular basis on which the chaperone binds its client proteins, Hsp110 prefer to bind aromatic rings as opposed to canonical Hsp70s that prefer aliphatic side chains and proline residues [68–69]. Additionally, the putative peptide binding domain of the *Plasmodium falciparum* Hsp110c was shown to be modified to handle the asparagine repeat-rich proteome of the parasite particularly during febrile episode [70].

**Fig 3:**
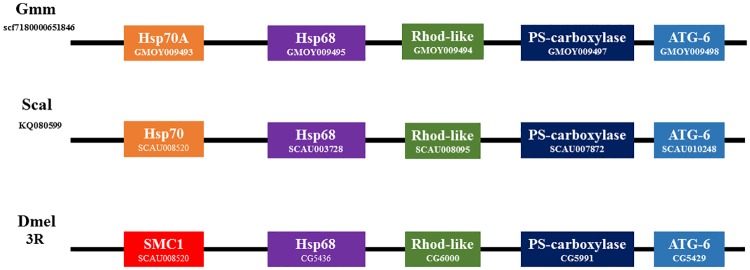
Schematic representation of the conserved synteny blocks neighbouring GmmHsp70A and GmmHsp68. Note that the gene order and orientation is relatively conserved. Abbreviations: SMC1, Structural maintenance of chromosomes 1; Rhod-like, Rhodanese-like domain protein; PS-carboxylate, Phosphatidylserine decarboxylase; ATG-6, autophagy-related protein 6. Accession numbers for each gene is shown in each synteny block.

Syntenic analysis provided additional evidence for orthology of selected members of the Hsp70 complement from *G*. *m*. *morsitans* which included GmmHsp68, GmmHsp70A, and GmmHsp110-2. Even though the genome of *G*. *m*. *morsitans* has yet to be assembled into chromosomes, position of these genes and their neighbouring genes were identified from the genome scaffolds on VectorBase [17]. GmmHsp68 and GmmHsp70A formed a clade with the Hsp68 and Hsp70A proteins from *D*. *melanogaster* and *S*. *calcitrans*, but as observed in Fig 1 the proteins did not exclusively phylogenetically cluster with their respective orthologues, and pBLAST analysis illustrated that the sequence identity of GmmHsp68 and GmmHsp70A is relatively similar to both the Hsp68 and Hsp70A proteins from *D*. *melanogaster* and *S*. *calcitrans* (S1 Table). Syntenic analysis revealed that the *GmmHsp70A* and *GmmHsp68* genes are located on the same chromosome in a head to head orientation, with the same genomic organisation being observed in *S*. *calcitrans* (Fig 3). Physical mapping of gene regions from *Drosophila serrata* illustrated that the *Hsp70* and *Hsp68* genes are located on the same chromosome [71], and the chromosomal gene position changes observed in *D*. *melanogaster* (Fig 3) may be a result of duplication/deletion events, and the movement of transposable elements [72–73]. Despite this, the neighbouring genes of Hsp68 are shown to be conserved among the three-dipteran species (Fig 3), supporting the orthologous relationship of GmmHsp68 to the Hsp68 proteins in *D*. *melanogaster* and *S*. *calcitrans*, and the orthologous relationship of GmmHsp70A to ScalHsp70.

**Fig 4:**
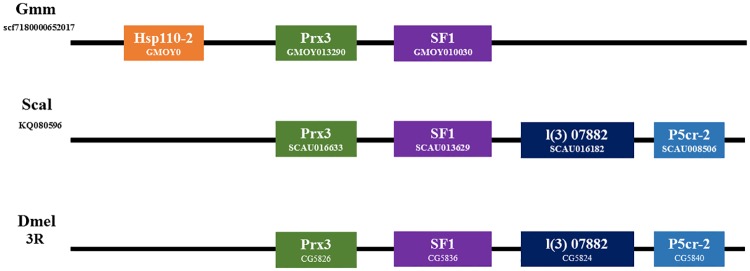
Schematic representation of the conserved synteny blocks neighbouring GmmHsp110-2. Note that the gene order and orientation is relatively conserved. Abbreviations: Prx3, Peroxiredoxin 3; SF1, Splicing factor 1; l (3) 07882, lethal (3) 07882; P5cr-2, Pyrroline-5-carboxylate reductase-like 2.

Syntenic analysis of GmmHsp110-2, as shown in Fig 4, was conducted in order to validate that the Hsp110 protein is specific to *G*. *m*. *morsitans*, as it was the only dipteran species in our study to possess two cytosolic Hsp110 protein members (Fig 2, Table 1). Syntenic analysis illustrated that GmmHsp110-2 is on the same region of the chromosome as Peroxiredoxin 3 (Prx3) and Splicing factor 1 (SF1) (Fig 3). The gene order and orientation of Peroxiredoxin 3 (Prx3) and Splicing factor 1 (SF1), as shown in Fig 4, is conserved in all three-dipteran species, but notably absent is a Hsp110 protein in *S*. *calcitrans* and *D*. *melanogaster*. Further neighbouring genes of Peroxiredoxin 3 (Prx3) and Splicing factor 1 (SF1) in *S*. *calcitrans* and *D*. *melanogaster* were shown to be conserved. However, these genes were also absent in *G*. *m*. *morsitans* (Fig 4). Overall, the genomic organisation of GmmHsp110-2 shows that it is a unique cytosolic Hsp110 protein to *G*. *m*. *morsitans* and may have arisen due to a duplication event.

### The J-protein complement

**Fig 5:**
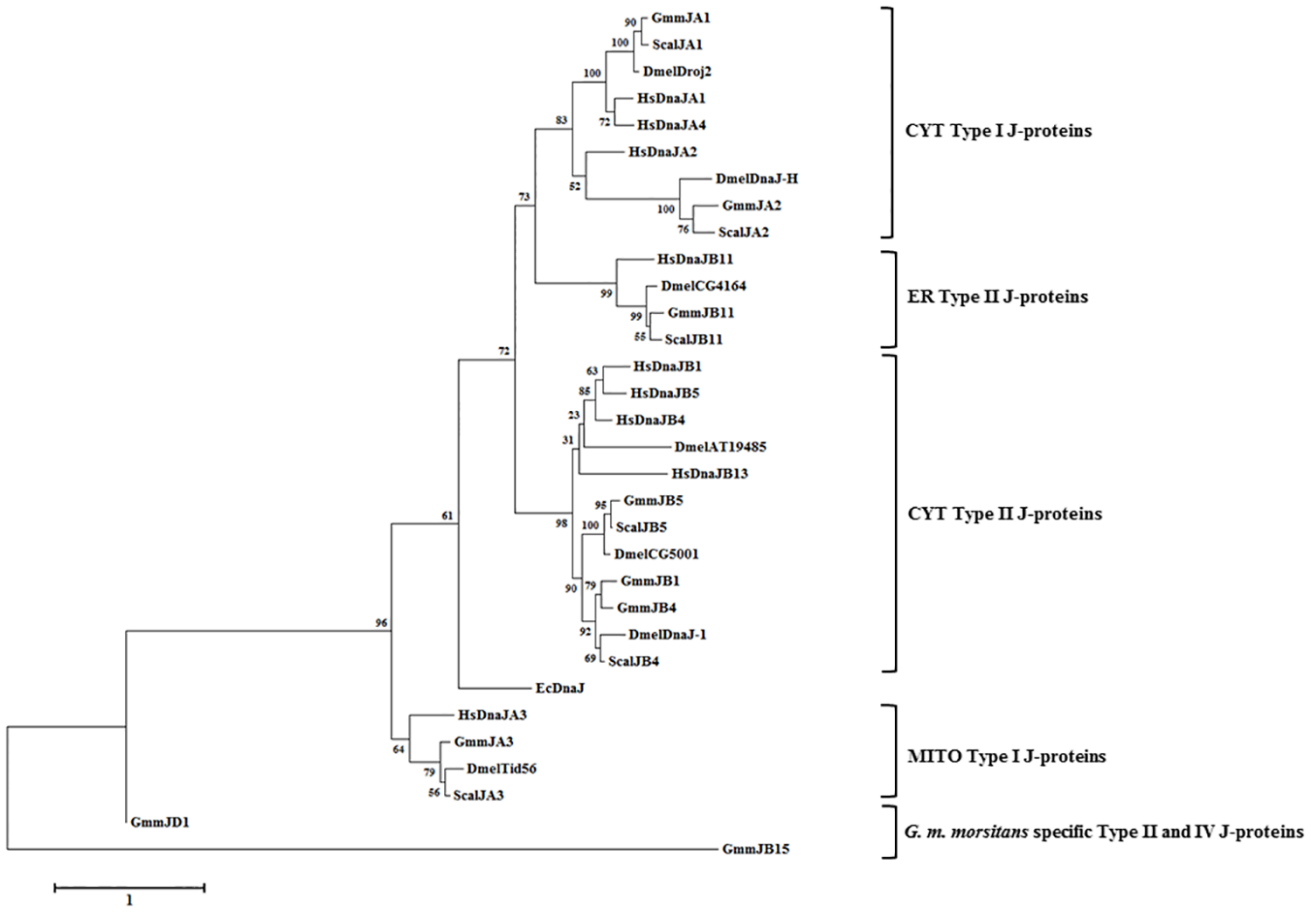
Phylogenetic analysis of the predicted Type I, II, and IV J-protein subfamilies from *G*. *m*. *morsitans* in relation to *D*. *melanogaster*, *H*. *sapiens and S*. *calcitrans*. A neighbour-joining tree was constructed using a multiple sequence alignment of the full-length amino acid sequences of the Type I, II, and IV J-protein subfamilies in humans, tsetse flies, fruit flies, and yeast. The multiple sequence alignment (S3 Fig) was performed using the in-built ClustalW program [43] with default parameters on the MEGA7 software [44]. The phylogenetic tree was constructed by MEGA 7 using the Maximum-likelihood method based on the Le Gascuel (LG) matrix-based model of amino acid substitution. A discrete Gamma distribution was used to model evolutionarily rate differences among sites (2 categories (+G, parameter = 2.3202). The alignment gaps were excluded from the analysis, and the number of amino acid sites used to construct the tree numbered 159. Bootstrap analysis was computed with 1000 replicates. Accession numbers of the sequences used: *E*. *coli*: DnaJ (NP_308042.1). *S*. *calcitrans*: ScalJA1 (SCAU009538); ScalJA2 (SCAU013613); ScalJA3 (SCAU003912); ScalJB4 (SCAU013247); ScalJB5 (SCAU015003); ScalB11 (SCAU015416). *D*. *melanogaster*: DnaJ-1 (NP_523936.2); CG5001 (NP_608586.2); AT19485 (NP_572633.1); Droj2 (NP_650283.1); Tid56 (NP_524932.2); DnaJ-H (NP_609605.1); CG4164 (NP_608525.1). *H*. *sapiens*: DnaJA1 (NP_001530.1); DnaJA2 (NP_005871.1); DnaJA3 (NP_005138.3); DnaJA4 (NP_061072.3); DnaJB1 (NP_006136.1); DnaJB4 (NP_008965.2); DnaJB5 (NP_001128476.2); DnaJB11 (NP_057390.1); DnaJB13 (NP_705842.2). Accession numbers for the *G*. *m*. *morsitans* J-protein sequences can be found in Table 2. The subcellular localisation for the J-proteins are indicated by a bracket on the right-hand side. CYT: cytosolic; ER: endoplasmic reticulum; MITO: mitochondrion.

**Fig 6:**
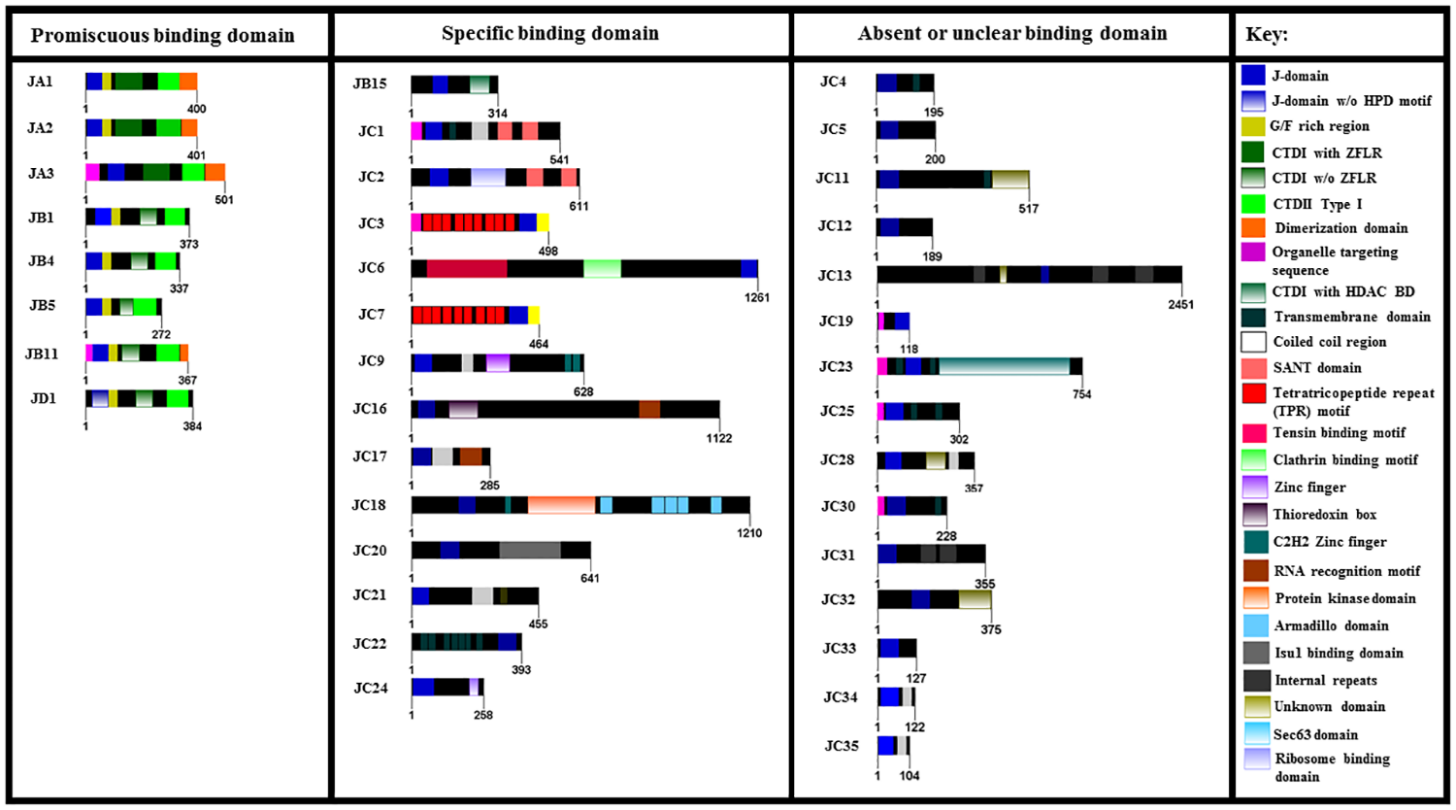
Schematic representation of the domain architecture of the different classes of J-proteins in *G*. *m*. *morsitans*. Each protein sequence for the *G*. *m*. *morsitans* J-protein family is represented by an open bar with the number of amino acids indicated on either side of the protein bar. The name of the respective J-protein is indicated on the left-hand side. The various domains are highlighted by coloured blocks within the protein bar. A key is provided to give a short description of the various domains and features. The J-proteins were also categorized based on assumed client binding ability and mechanistic mode of functioning as proposed by Kampinga et al. [22].

The J-protein complement for *G*. *m*. *morsitans* was identified through a genome-wide search using the J-domain from *Escherichia coli* DnaJ, as the J-domain is the signature region for all J-proteins [33]. A total of 37 J-proteins were identified in the *G*. *m*. *morsitans* genome. All retrieved amino acid sequences of the J-proteins were full-length sequences on VectorBase database except for GmmJC33 (GMOY003881) and GmmJC34 (GMOY004160/1), which are partial sequences. All J-proteins were further categorized into the 4 J-protein subfamilies, I-IV. Nomenclature proposed for the Gmm J-proteins was based on the guidelines in Kampinga et al. [22], except GmmJD was devised to incorporate Type IV J-proteins. Types I to IV in Gmm are designated as A-D respectively. Nomenclature for the Scal J-proteins were derived in the same manner. The predicted subcellular localisations, identification of orthologues and functional diversity of the Gmm J-proteins are summarized in Table 2. The results of the pBLAST analysis to determine the orthologous relationship of the Gmm J-proteins to the selected organisms in this study are presented in S1 Table. Phylogenetic analysis of the selected J-protein subfamilies as illustrated in Fig 5, shows that the J-proteins cluster based on their different classes and subcellular localisation. A comprehensive domain organisation of the predicted Gmm J-proteins is illustrated in Fig 6.

**Table 2:** The predicted J-proteins from *G*. *m*. *morsitans* with their respective *D*. *melanogaster*, *H*. *sapiens and S*. *calcitrans* orthologues.

	*G*. *m*. *morsitans*^b^		*D*. *melanogaster*^c^		*S*. *calcitrans*		*H*. *sapiens*^c^			
Type	Name^a^	Gene ID	Name^a^	Gene ID	Name	Gene ID		Localisation^d^	Function^e^	References
**I**	GmmJA1	GMOY007075	Droj2	FBgn0038145	ScalJA1	SCAU009538	DnaJA1	CYT	Androgen receptor signalling	[76; 107]
								NUC	Spermatogenesis	
									hERG maturation and trafficking	
									Protein aggregation and refolding	
									Protein import-MITO	
	GmmJA2	GMOY002478	DnaJ-H	FBgn0032474	ScalJA2	SCAU013613	DnaJA2	CYT	hERG maturation and trafficking	[77; 107–108]
									JNK signalling	
									G-protein signalling	
	GmmJA3	GMOY006702	Tid56	FBgn0002174	ScalJA3	SCAU003912	DnaJA3	MITO	Intracellular signalling pathways	[95]
									Tumour suppressor	
									Protein aggregation and folding	
									mtDNA maintenance	
**II**	GmmJB1	GMOY005834	-	-	-	-	DnaJB1	CYT	Protein (re)folding	[109]
	GmmJB4	GMOY003219	DnaJ-1	FBgn0263106	ScalJB4	SCAU013247	DnaJB4	CYT	ERAD	[110]
								NUC		
	GmmJB5	GMOY007851	CG5001	FBgn0031322	ScalJB5	SCAU015003	DnaJB5	CYT	HDAC shuttling	[111]
								NUC		
	GmmJB11	GMOY005166	CG4164	FBgn0031256	ScalJB11	SCAU015416	DnaJB11	ER	Protein folding	[112]
									mRNA editing	
	GmmJB15	GMOY001519	-	-	-	-	-	MITO?	?	-
**III**	GmmJC1	GMOY000574	CG7556	FBgn0030990	ScalJC1	SCAU000575	DnaJC1	ER	ACT secretion	[113–114]
								PLASMA	Protein folding	
								MEM		
	GmmJC2	GMOY000389	CG10565	FBgn0037051	ScalJC2	SCAU003775	DnaJC2	CYT	Translation	[115]
								NUC		
	GmmJC3	GMOY009982	P58IPK	FBgn0037718	ScalJC3	SCAU013977	DnaJC3	ER	Viral pathogenesis	[116–117]
									ER protein synthesis	
									UPR	
	GmmJC4	GMOY002749	DnaJ60	FBgn0260775	ScalJC4	SCAU004329	DnaJC4	CYT	Spermatogenesis	[118]
	GmmJC5	GMOY000122	Cysteine string protein?	FBgn0004179	ScalJC5	SCAU013958	DnaJC5?	CYT	Synaptic transmission	[119]
									Exocytosis	
	GmmJC6	GMOY012999	Auxilin	FBgn0037218	ScalJC6	SCAU011318	DnaJC6	CYT	Clathrin uncoating	[120]
	GmmJC7	GMOY010330	TPR2A	FBgn0032586	ScalJC7	SCAU011528	DnaJC7	CYT	Steroid hormone maturation	[121]
								NUC	Protein folding quality control	
	GmmJC9	GMOY010007	CG6693	FBgn0037878	ScalJC9	SCAU003671	DnaJC9	NUC	Anti-protein aggregation	[122]
									Nuclear exit upon stress	
	GmmJC11	GMOY010852	CG8531	FBgn0033918	ScalJC11	SCAU005931	DnaJC11	MITO	Mitochondrial cristae morphology	[123]
								PLASM		
	GmmJC12	GMOY000552	Jdp	FBgn0027654	ScalJC12	SCAU015463	DnaJC12	CYT	Anti-protein aggregation	[124]
	GmmJC13	GMOY009168	Rme-8	FBgn0015477	ScalJC13	SCAU006475	DnaJC13	CYT	EGFR trafficking	[125–126]
								MITO	Endosome trafficking	
	GmmJC16	GMOY002603	CG40178	FBgn0058178	ScalJC16	SCAU006018	DnaJC16	PLASM	?	-
	GmmJC17	GMOY011131	CG17187	FBgn0037882	ScalJC17	SCAU016288	DnaJC17	CYT	Pre-mRNA splicing	[127]
								NUC		
	GmmJC18	GMOY004658	-	-	ScalJC18	SCAU005547	-	PLASM	?	-
	GmmJC19	GMOY002685	CG7394	FBgn0036173	ScalJC19	SCAU007810	DnaJC19	MITO	Protein import-MITO	[127]
	GmmJC20	GMOY000945	l (3)72Dp	FBgn0263607	ScalJC20	SCAU000691	DnaJC20	MITO	FeS cluster biogenesis	[128–129]
	GmmJC21	GMOY006819	CG2790	FBgn0027599	ScalJC21	SCAU016142	DnaJC21	CYT	Ribosome biogenesis	[127]
								NUC		
	GmmJC22	GMOY009242	Wurst	FBgn0030805	ScalJC22	SCAU013054	DnaJC22	PLASM	Clathrin-mediated endocytosis	[130]
	GmmJC23	GMOY008009	Sec63	FBgn0035771	ScalJC23	SCAU014339	DnaJC23	ER	Protein import	[131]
								PLASM		
	GmmJC24	GMOY009973	CG2911	FBgn0037350	ScalJC24	SCAU012042	DnaJC24	CYT	Dipthamide synthesis	[132]
								NUC		
	GmmJC25	GMOY003438	CG7872	FBgn0030658	ScalJC25	SCAU002837	DnaJC25	SEC	?	-
	GmmJC28	GMOY003297	CG43322	FBgn0263027	ScalJC28	SCAU012595	DnaJC28	CYT	?	-
	GmmJC30	GMOY001329	CG11035	FBgn0037544	ScalJC30	SCAU007712	DnaJC30	MITO	?	-
	GmmJC31	GMOY007250	Mrj	FBgn0034091	ScalJC31	SCAU012526	DnaJB3	CYT	Protein folding in sperm	[127]
	GmmJC32	GMOY005661	CG3061	FBgn0038195	ScalJC32	SCAU005586	DnaJB12	CYT	ERAD	[133]
	GmmJC33	GMOY003881	CG17187?	FBgn0037882	ScalJC17	SCAU016288	DnaJC17?	CYT	?	-
	GmmJC34	GMOY004160	CG17187?	FBgn0037882	ScalJC17	SCAU016288	DnaJC17?	CYT	?	-
		GMOY004161						NUC		
	GmmJC35	GMOY007968	CG17187?	FBgn0037882	ScalJC17	SCAU016288	DnaJC17?	CYT	?	-
**IV**	GmmJD1	GMOY011949	-	-	-	-	-	CYT	?	-

^a^ The proposed nomenclature for the J-proteins of *G*. *m*. *morsitans* and *S*. *calcitrans*; these J-proteins were classified into Types I-IV (A-D).

^b^ Gene IDs for *G*. *m*. *morsitans* and *S*. *calcitrans* were obtained from VectorBase (https://www.vectorbase.org/; [17]).

^c^ Orthologues identified from *Homo sapiens*, *Stomoxys calcitrans*, and *Drosophila melanogaster* by NCBI database analysis.

^d^ Subcellular localizations for the *G*. *m*. *morsitans* and *S*. *calcitrans* J-proteins were predicted using the online prediction servers listed in the methods. Subcellular localizations have been experimentally determined for certain J-proteins from *Homo sapiens*, and *Drosophila melanogaster* (see text for details).

CYT-Cytosol; MITO- Mitochondria; NUC- Nucleus; ER- Endoplasmic reticulum; PLASM- Plasma membrane; SEC- Secreted.

^e^ The predicted cellular role and functions for each J-protein from *G*. *m*. *morsitans* were implied from either Gene Ontology [127], or published literature on the identified functions/cellular roles of their identified Drosophila and human orthologues.

The large and diverse family of J-proteins contain a number of domains, which have been used as the basis for classification of J-proteins into four different classes [33–34]. The basis for classification of a J-protein is their homology to the prokaryotic canonical J-protein, DnaJ [33]. The domain architecture of DnaJ is divided into an N-terminal J-domain, glycine-phenylalanine (G/F) rich region, zinc finger-like region (ZFLR), and a C-terminal peptide binding domain [33]. The C-terminal domain is comprised of two-barrel topology domains, CTDI and CTDII. CTDI has a hydrophobic pocket for peptide binding and a zinc-finger domain which may also bind peptides [25]. Type I J-proteins possess all these canonical domains, and thus, are highly conserved with respect to DnaJ [32]. Type II J-proteins lack the zinc finger-like region, which is substituted by a glycine-methionine (G/M) rich region [32]. Type III J-proteins contain only the signature J-domain which can occur anywhere along the protein sequence [33]. Type IIIs also possess specialized domains that assist in localizing the J-protein to certain locations within the cell, and specifying the clientele for substrate binding [34]. Type IV proteins possess a J-domain with a compromised or absent HPD motif and may also possess domain structures from other J-protein types [33].

#### Type I J-proteins

This study identified that the Type I J-protein subfamily in *G*. *m*. *morsitans* has three members: GmmJA1, GmmJA2, and GmmJA3 (Table 2). GmmJA1 and GmmJA2 are Type I J-proteins that are predicted to reside in the cytosol based on their orthology and phylogeny (Fig 5, Table 2), and thus are proposed to assist the predicted cytosolic Gmm Hsp70s in promoting the folding of nascent polypeptides. Though the main role of J-proteins is co-chaperone to their Hsp70 partner, a growing number of cellular roles independent of Hsp70 have been established [74]. The mammalian orthologue of GmmJA1, DnaJA1 has been shown to independently associate and prevent the aggregation of unfolded proteins [75], and is a regulator in the maturation of the androgen receptor (AR) [76]. DnaJA2, mammalian orthologue of GmmJA2, is an enhancer of G-protein-coupled signalling by the β2-adrenergic receptor [77], and assists in the ER-associated degradation of HERG potassium channels by the ubiquitin-proteasome system [78]. Despite their strong homology, the deletion of DnaJA1 in mammalian cells and mice could not be compensated by DnaJA2 and vice versa [76]. A study conducted by Baaklini and colleagues [79] showed that the substrate release mechanism and apparent conformations of DnaJA2 is biochemically different to DnaJA1, and it is inferred as one of the reasons for their functional divergence in their Hsp70 dependent and independent roles.

Loss of DnaJA1 in mice results in severe defects in the late stages of spermatogenesis due to aberrant AR signalling [76]. However, the biological and biochemically properties of GmmJA1 need to be first elucidated. Both GmmJA1 and GmmJA2 were found to possess CTSS and CQTG C-terminal CaaX motifs respectively (S1 Fig), which play a role in protein isoprenylation and farnesylation [80–82]. This post-translational modification has been observed to be integral to the proper functioning of Type I J-proteins, as alternation of this motif within Ydj1 (Type I J-protein from *Saccharomyces cerevisiae*) resulted in the development of a temperature-sensitive growth phenotype in *S*. *cerevisiae* as the motif redirects J-proteins to the plasma membrane or to multi-protein complexes that require its function under stressful conditions [80]. Farnesylation of the CaaX motif has been shown to influence Ydj1 co-operation with Hsp90 [82], and the transferring of substrates to Hsp70 [83].

GmmJA3 is predicted to localise in the mitochondria as it clusters with the known mitochondrial Type I J-proteins HsDnaJA3, and DmelTid56 (Fig 5), and has a N-terminal mitochondrial signal peptide (Fig 6). DmelTid56 is a J-protein that was first discovered as a tumour suppressor, as the deletion of the *tid56* gene lead to malignant growth of imaginal disc cells and subsequent embryonic lethality [84]. DnaJA3, the mammalian counterpart of Tid56, has also been shown to be critical for early embryonic development [85], though its role in oncogenesis is controversial [86]. The *dnaJA3* gene encodes for two alternatively spliced forms of the protein, which exhibit opposing biological functions in response to exogenous cytotoxic stimuli [87]. The human and *Drosophila* orthologues of GmmJA3, have been shown to co-operate with mitochondrial Hsp70 in the folding of mitochondrial synthesized and newly imported proteins within the mitochondrial matrix. Thus, GmmJA3 potentially interacts with GmmHsc70-5 in the mitochondria to promote protein folding and disaggregate toxic proteins.

#### Type II J-proteins

Our study revealed that 5 Type II J-proteins are present in the *G*. *m*. *morsitans* J-protein complement (Table 2). The domain architecture of Type II J-proteins is similar to the Type I J-proteins, except that the zinc finger region that is protruding from the client binding cleft (CTDI) in Type I J-proteins is absent [88]. Despite the difference in the CTDI, Type II J-proteins have been shown to bind non-native substrates, and promote folding in conjunction with Hsp70 [89]. Interestingly, the CTDI of Type II J-proteins have been shown to bind to their cytosolic Hsp70 partners via the C-terminal EEVD motif [90], and it would be interesting to investigate whether GmmJB1 displays the same stringent binding requirements in order to mediate the (re)folding of client proteins. DnaJ-1, *Drosophila* orthologue of GmmJB4, was shown to interact with Hsc70Cb/Hsp110 in suppressing polyglutamine-induced cell death in *Drosophila*, and thus, these proteins may function together to maintain protein homeostasis [91]; whilst DnaJ-1 suppressed the toxicity of aggregated proteins, Droj2 and CG5001 lacked this function. It could be proposed that GmmJB4 may also interact with either or both GmmHsp110-1 and GmmHsp110-2 in the same manner within the cell, and carry out the same role in maintaining protein homeostasis as its *Drosophila* orthologues.

GmmJB11 is a predicted ER Type II J-protein as it forms a clade with the ER luminal Type II J-protein, HsDnaJB11 (Fig 5), and the domain architecture of GmmJB11 shows it possesses an N-terminal signalling peptide (Fig 6). HsDnaJB11 has been experimentally shown to localize within the ER [92], where it binds directly to several nascent, unfolded and mutant secretory proteins, and presents them for HSPA5-dependent folding [93]. The expression of DnaJB11 has also been shown to be up-regulated in response to unfolded secretory protein stress, and is an integral part of the ER stress response [92–93]. The functionality of HSPA5 is highly dependent on its interaction with ER J-proteins during homeostasis and stress, as blocking the partnership will significantly impact HSPA5-dependent folding *in vivo* [94]. Thus, knockdown or inhibition of GmmJB11 and subsequently its partnership with GmmHsc70-3 could impede the secretion of nascent proteins from the ER. Though, elucidating the role of GmmJB11 and potential GmmJB11-GmmHsc70-3 partnership needs to be conducted.

Thioredoxin1 (Trx1) targets the *dnaJB5* gene, the human orthologue of GmmJB5, resulting in an up-regulation of gene expression; DnaJB5 then recruits TBP-2, and orchestrates the formation of the Trx1-DnaJB5-TBP2 complex which mediates the reduction of class II histone deacetylases (HDAC4), essentially restoring its nuclear localisation [95–96]. The reduction of HDAC4 enables the transfer of NADPH-generated electrons to downstream targets, which in turn regulates cardiac hypertrophy [95–96]. The RNA-mediated knockdown of HDAC4 within *Drosophila* clock cells has been shown to impair the circadian rhythm [97], and long-term memory development within *Drosophila* [98]. Therefore, it will be interesting to explore the effect of knockout or inhibition of DnaJB5, and its subsequent effect on the function of HDAC4.

GmmJB15 is a Type II J-protein that is unique to the tsetse fly as it has no orthologues in the selected organisms in this study (Table 2). Phylogenetic analysis reinforces that this Type II J-protein is specific to *G*. *m*. *morsitans* as it forms a distinct clade on the tree (Fig 5). However, the domain architecture of GmmJB15 is similar to the human Type II J-proteins: DnaJB6, DnaJB7, and DnaJB8 due to the presence of a HDAC binding domain in the CTDI of GmmJB15 (Fig 6). DnaJB6 and DnaJB8 have been shown to be the two most potent suppressors of aggregation and related toxicity of expanded polyQ proteins [99]. Though, the functional role of DnaJB7 and the HDAC domain have not yet been determined, and therefore no infer of possible function can be made for GmmJB15. However, it raises interesting questions on the biological role of GmmJB15 within the tsetse fly, and it should be prioritized for future studies.

#### Type III J-proteins

The majority of J-proteins are often comprised of the Type III J-protein subfamily, and *G*. *m*. *morsitans* is no exception as 76% of the J-proteins are Type III J-proteins (Table 2). The functional diversity of the J-protein complement is predominately due to the Type III J-proteins as these members possess a variety of protein domains and motifs, as illustrated in Fig 6, that enable these members to carry out diverse functions within the cell [35]. Eleven of the identified Type III J-proteins (GmmJC2, GmmJC4-7, GmmJC12-13, GmmJC17, GmmJC21, GmmJC24, GmmJC28, and GmmJC31-35) were predicated to localize within the cytosol, with six of these also predicted to be exported to the nucleus, and one exported to the mitochondria (Table 2). Three Type III J-proteins were predicted to localize in the mitochondria (GmmJC19, GmmJC20, and GmmJC30) and one in the ER (GmmJC3) (Table 2). Many of the J-proteins were predicted to associate with the plasma membrane of the cell or subcellular compartments (Table 2), as several of the J-proteins were shown to possess transmembrane domains (Fig 6).

Despite the fact that the human J-proteins DnaJB3 and DnaJB12 are categorised as Type II J-proteins [25], their Gmm orthologues, GmmJC31 and GmmJC32, have been categorised as Type III due to the identification of only a J-domain (Fig 6, Table 2). All of the predicted Type III J-proteins were found to possess human and *Drosophila* orthologues, and thus could possess similar functions/roles to their identified orthologues (Table 2). GmmJC5, despite its orthology to HsDnaJC5 and the cysteine string protein in *D*. *melanogaster*, does not contain the characteristic cysteine-rich region for palmitoylation, and subsequent export to the post-Golgi membranes (Fig 5) [100]. Studies conducted on *Drosophila* demonstrated that the loss of cysteine string protein expression has been reported to result in very rapid death of adult flies [101]. However, the absence of the cysteine-rich region may possibly be the result of a miss-annotation of the coding region, or sequencing error of GmmJC5.

Additional investigations of miss-annotations/sequencing errors of the *G*. *m*. *morsitans* genome include GmmJC33 and GmmJ34 as these appear to be partial amino acid sequences. The domain architecture for these J-proteins is entirely comprised of the J-domain (Fig 6). Both these J-proteins are putative orthologues of HsDnaJC17, and indicated to be involved in pre-mRNA splicing [102]. Though, this is inconclusive due to the absence of the RNA recognition motif and spliceosome interaction domain that are present in its human counterpart [25].

#### Type IV J-proteins

Type IV J-proteins are characterized by a J-domain with an abrogated or absent HPD motif [34]. GmmJD1 was the only Type IV J-protein identified in the *G*. *m*. *morsitans* genome and phylogenetic analysis revealed that this J-protein is a unique Type IV J-protein to *G*. *m*. *morsitans* (Fig 4). GmmJD1 was shown to possesses a HNY motif, but also the canonical domains of a typical Type I J-protein (Fig 6). DnaJB13 is the only mammalian J-protein that has a J-domain with an imperfect HPD motif, as it has a HPL motif instead [22]. Due to the abrogated HPD motif, it was questioned whether DnaJB13 could serve as a typical J-protein as the HPD residues are critical to the function of the J-domain. However, it has been shown that DnaJB13 is a cytosolic J-protein involved in the process of spermiogenesis, and sperm movement [103–104]. It would be interesting to investigate the cellular role of GmmJD1, and whether it forms a potential partnership with the Gmm Hsp70s. It marks another J-protein that should be prioritized for future studies.

#### Expression of Hsp70 and J-protein genes in *G*. *m*. *morsitans* after trypanosome infection

Transcriptomic analysis of trypanosome-infected tsetse flies revealed an increase in the expression of GmmHsp70s, in particular Hsc70-3 (GMOY003216), 4 (GMOY012049), 5 (GMOY010851) and Hsp110-1 (GMOY011246), linked to structural damage of the salivary glands in comparison to uninfected flies, and induction of the stress response could be used as a tool to aid cell renewal [18]. An additional detailed transcriptomic study to determine the effect of trypanosome infection on the salivary gland functions of tsetse flies revealed that a number of genes encoding heat shock proteins were differentially expressed [105]. In a comparison of flies with a mature parasite infection in the salivary glands versus non-infected flies, Hsp70/Hsp90 organising protein (HOP; GMOY003596), Hsc70-5, Hsp110-2 (GMOY013289), JA1 (GMOY007075) were moderately upregulated. Whilst Hsc70-3, Grp170 (GMOY006943), JC16 (GMOY002603) and JC31 (GMOY007250) were moderately down regulated [105]. A further comparison of flies with a mature parasite infection in the salivary glands versus flies with only an established midgut infection revealed that Hsp70A (GMOY009493), HOP and JB4 (GMOY003219) were moderately upregulated [105]. Interestingly, GmmHsp110-2, the Hsp110 unique to *G*. *m*. *morsitans* identified also in this study, was the only heat shock protein that showed increased expression in the salivary glands of flies with an existing trypanosome infection in the midgut in comparison to uninfected flies, which suggests a preliminary response in the salivary glands ahead of parasite infection [105]. GmmHsp110-2 may also be essential for viability and prevention of protein aggregation during stress conditions in the tsetse fly.

## Conclusion

The Hsp70 and J-protein complements were comparatively analysed in relation to those found in *D*. *melanogaster*, *H*. *sapiens*, and *S*. *calcitrans*. This study resulted in the identification of 9 putative Hsp70 proteins. The arrangement of the 6 inducible Hsp70 proteins in *Drosophila* was absent in *G*. *m*. *morsitans* and *S*. *calcitrans*. The Hsc70 proteins in *Drosophila* are regulated during development and exhibit cell and tissue specificity, the same will probably be true of *G*. *m*. *morsitans*.

In this study 37 J-proteins were identified, with two of these being partial sequences. Based on the available data from the eukaryotic orthologues, it was possible to infer functions of many of the Hsp70 and J-proteins from *G*. *m*. *morsitans*. Obviously, many of our inferences will need be to be confirmed experimentally. The diversity of the J-protein complement has evolved to fulfil specific functions. Some heat shock proteins from the trypanosomes have been studied and those essential for differentiation and survival have been identified [106]. A comparative analysis of the Hsp70-J-protein complex from the human and animal hosts, as well as the *Trypanosoma brucei* parasites and the insect vectors will enhance our understanding of the differences in host specificities, in addition it will be possible to gain a better understanding of vector-parasite and host-parasite interactions.

## Supporting information

S1 Fig Alignment of the predicted Hsp70/HSPA family from *G*. *m*. *morsitans* (Gmm) in relation to *D*. *melanogaster* (Dmel), *H*. *sapiens* (Hs) and *S*. *calcitrans* (Scal). Multiple sequence alignment of the full-length amino acid sequences of the Hsp70/HSPA gene families in humans, tsetse flies, fruit flies, and stable flies. The multiple sequence alignment was performed using the in-built ClustalW program [43] with default parameters on the MEGA7 software [44]. Degree of amino acid conservation is symbolized by the following: (*) all fully conserved residues; (:) one of the residues is fully conserved and (.) residues are weakly conserved. Accession numbers of the sequences used: *E*. *coli*: HscC (NP_415183.1). *S*. *calcitrans*: Hsp70 (SCAU008520); Hsp68 (SCAU003728); Hsc70-1 (SCAU005225); Hsc70-2 (SCAU008036); Hsc70-3 (SCAU000678); Hsc70-4 (SCAU015347); Hsc70-5 (SCAU003620). *D*. *melanogaster*: Hsp68 (NP_524474.1); Hsp70Aa (NP_731651.1); Hsp70Ab (NP_524798.2); Hsp70Ba (NP_731716.1); Hsp70Bb (NP_524927.2); Hsp70Bbb (NP_788663.1); Hsp70Bc (NP_650209.1); Hsc70-1 (NP_524063.1); Hsc70-2 (NP_524339.1); Hsc70-3 (NP_727563.1); Hsc70-4 (NP_524356.1); Hsc70-5 (NP_523741.2). *H*. *sapiens*: HSPA1A (NP_005336.3); HSPA1B (NP_005337.2); HSPA1L (NP_005518.3); HSPA2 (NP_068814.2); HSPA5 (NP_005338.1); HSPA6 (NP_002146.2); HSPA8 (NP_006588.1); HSPA9 (NP_004125.3). Accession numbers for the *G*. *m*. *morsitans* Hsp70 sequences can be found in Table 1.
(PDF)

S2 Fig Alignment of the Hsp110/HSPH protein family from *G*. *m*. *morsitans* (Gmm) in relation to *D*. *melanogaster* (Dmel), *H*. *sapiens* (Hs) and *S*. *calcitrans* (Scal). Multiple sequence alignment of the full-length amino acid sequences of the Hsp110/HSPH gene families in humans, tsetse flies, fruit flies, and yeast. The multiple sequence alignment was performed using the in-built ClustalW program [43] with default parameters in the MEGA7 software [44]. Degree of amino acid conservation is symbolized by the following: (*) all fully conserved residues; (:) one of the residues is fully conserved and (.) residues are weakly conserved. Accession numbers of the sequences used: *E*. *coli*: HscC (NP_415183.1). *S*. *calcitrans*: Hsp110 (SCAU005995); Grp170 (SCAU010922). *D*. *melanogaster*: Hsp110 (NP_648687.1); Grp170 (NP_569995.1). *H*. *sapiens*: HSPH1 (NP_006635.2); HSPH2 (NP_002145.3); HSPH3 (NP_055093.2); HSPH4 (NP_006380.1). Accession numbers for the *G*. *m*. *morsitans* Hsp110 sequences can be found in Table 1.
(PDF)

S3 Fig Alignment of the predicted Type I, II, and IV J-protein subfamilies from *G*. *m*. *morsitans* (Gmm) in relation to *D*. *melanogaster* (Dmel), *H*. *sapiens* (Hs) and *S*. *calcitrans* (Scal). The multiple sequence alignment was performed using the in-built ClustalW program [43] with default parameters on the MEGA7 software [44]. Degree of amino acid conservation is symbolized by the following: (*) all fully conserved residues; (:) one of the residues is fully conserved and (.) residues are weakly conserved. Accession numbers of the sequences used: *E*. *coli*: DnaJ (NP_308042.1). *S*. *calcitrans*: ScalJA1 (SCAU009538); ScalJA2 (SCAU013613); ScalJA3 (SCAU003912); ScalJB4 (SCAU013247); ScalJB5 (SCAU015003); ScalB11 (SCAU015416). *D*. *melanogaster*: DnaJ-1 (NP_523936.2); CG5001 (NP_608586.2); AT19485 (NP_572633.1); Droj2 (NP_650283.1); Tid56 (NP_524932.2); DnaJ-H (NP_609605.1); CG4164 (NP_608525.1). *H*. *sapiens*: DnaJA1 (NP_001530.1); DnaJA2 (NP_005871.1); DnaJA3 (NP_005138.3); DnaJA4 (NP_061072.3); DnaJB1 (NP_006136.1); DnaJB4 (NP_008965.2); DnaJB5 (NP_001128476.2); DnaJB11 (NP_057390.1); DnaJB13 (NP_705842.2). Accession numbers for the *G*. *m*. *morsitans* J-protein sequences can be found in Table 2.
(PDF)

S4 Fig Schematic representation of the domain architecture of the predicted *G*. *m*. *morsitans* Hsp70 superfamily. Each protein sequence for the *G*. *m*. *morsitans* Hsp70 superfamily is represented by an open bar with the various protein domains and other associated features that were identified using Prosite [44] and SMART 7 [43] are displayed as colour blocks within the open bar. These domains and associated features include the N-terminal ATPase domain (red), substrate binding domain (SBD; green), putative substrate binding domain for NEFs (SBD; dark green), C-terminal region (C-terminal; purple) and targeting signal peptides (S; dark blue).
(TIF)

S1 Table Orthologous relationship of the Hsp70 and J-protein complements from *G*. *m*. *morsitans* to *D*. *melanogaster*, *S*. *calcitrans*, and *H*. *sapiens*. (XLSX)
