# Supplementary material for: Hsp70/J-protein machinery from Glossina morsitans morsitans, vector of African trypanosomiasis
Source: PLoS One. 2017 Sep 13;12(9):e0183858. doi: 10.1371/journal.pone.0183858 (PMC5597180; doi:10.1371/journal.pone.0183858)
Supplement: S1 Fig — Multiple sequence alignment of the full-length amino acid sequences of the Hsp70/HSPA gene families in humans, tsetse flies, fruit flies, and stable flies. The multiple sequence alignment was performed using the in-built ClustalW program [43] with default parameters on the MEGA7 software [44]. Degree of amino acid conservation is symbolized by the following: (*) all fully conserved residues; (:) one of the residues is fully conserved and (.) residues are weakly conserved. Accession numbers of the sequences used: E. coli: HscC (NP_415183.1). S. calcitrans: Hsp70 (SCAU008520); Hsp68 (SCAU003728); Hsc70-1 (SCAU005225); Hsc70-2 (SCAU008036); Hsc70-3 (SCAU000678); Hsc70-4 (SCAU015347); Hsc70-5 (SCAU003620). D. melanogaster: Hsp68 (NP_524474.1); Hsp70Aa (NP_731651.1); Hsp70Ab (NP_524798.2); Hsp70Ba (NP_731716.1); Hsp70Bb (NP_524927.2); Hsp70Bbb (NP_788663.1); Hsp70Bc (NP_650209.1); Hsc70-1 (NP_524063.1); Hsc70-2 (NP_524339.1); Hsc70-3 (NP_727563.1); Hsc70-4 (NP_524356.1); Hsc70-5 (NP_523741.2). H. sapiens: HSPA1A (NP_005336.3); HSPA1B (NP_005337.2); HSPA1L (NP_005518.3); HSPA2 (NP_068814.2); HSPA5 (NP_005338.1); HSPA6 (NP_002146.2); HSPA8 (NP_006588.1); HSPA9 (NP_004125.3). Accession numbers for the G. m. morsitans Hsp70 sequences can be found in Table 1. (PDF) [file pone.0183858.s001.pdf]

```
EcHscC          -----------------------------------------------------MDNAELA      7
HsHSPA9         MISASRAAAARLVGAAASRGPTAARHQDSWN-----GLSHEAFRLVSRRDYASEAIKGAV      55
GmmHsc70-5      MLRIPKF-LPRILTQHQAN-LLGGRSSSIFENKLMQANSVLNNTLAAQFSQRSGEVKGAV      58
DmelHsc70-5     MLRVPKF-LPRLARQAGVVPSHMSGASSMFR-NLPG----ASNGISSQLRYKSGEVKGAV      54
ScalHsc70-5     MLRATKF-LPRLIGQRQATALVSGKNSSIFENQLAA----LNNGLAAQLRHKSGEVKGAV      55
HsHSPA5         -----------------------------MKLSLVAAMLLLLS-AARAEEEDKKEDVGTV      30
GmmHsc70-3      -----------------------------MRLLLILAITAFVGFSYGEEKKEKEKDIGTV      31
DmelHsc70-3     -----------------------------MKLCILLAVVAFVGLSLGEEKKEKDKELGTV      31
ScalHsc70-3     -----------------------------MRLLTLLAVVAFVGLALGEDKKEKDKDIGTV      31
DmelHsc70-4     ------------------------------------------------------MSKAPA      6
GmmHsc70-4      ------------------------------------------------------MSKAPA      6
ScalHsc70-4     ------------------------------------------------------MSKAPA      6
DmelHsc70-1     ------------------------------------------------------MPKLPA      6
GmmHsc70-1      ------------------------------------------------------MAKVPA      6
ScalHsc70-1     ------------------------------------------------------------      0
HsHSPA6         ----------------------------------------------------MQAPRELA      8
HsHSPA1A        ------------------------------------------------------MAKAAA      6
HsHSPA1B        ------------------------------------------------------MAKAAA      6
HsHSPA1L        ----------------------------------------------------MATAKGIA      8
HsHSPA2         -----------------------------------------------------MSARGPA      7
HsHSPA8         ------------------------------------------------------MSKGPA      6
GmmHsp68        ---------------------------------------------------------MVA      3
DmelHsc70-2     ------------------------------------------------------MGKIPA      6
ScalHsc70-2     ------------------------------------------------------MVKAPA      6
DmelHsp70Aa     ---------------------------------------------------------MPA      3
DmelHsp70Ab     ---------------------------------------------------------MPA      3
DmelHsp70Ba     ---------------------------------------------------------MPA      3
DmelHsp70Bb     ---------------------------------------------------------MPA      3
DmelHsp70Bc     ---------------------------------------------------------MPA      3
DmelHsp70Bbb    ---------------------------------------------------------MPA      3
ScalHsp70       ---------------------------------------------------------MVA      3
DmelHsp68       ---------------------------------------------------------MPA      3
GmmHsp70A       ---------------------------------------------------------MVA      3
ScalHsp68       ---------------------------------------------------------MVA      3

EcHscC          IGIDLGTTNSLIAVWKDGAAQLIPNKFGEYLTPSIISMDENNHILVGKPAVSRRTSHPDK      67
HsHSPA9         VGIDLGTTNSCVAVMEGKQAKVLENAEGARTTPSVVAFTADGERLVGMPAKRQAVTNPNN      115
GmmHsc70-5      IGIDLGTTNSCVAIMEGKQAKVIENAEGARTTPSHIAFTKDGERLVGMPAKRQAVTNAAN      118
DmelHsc70-5     IGIDLGTTNSCLAVMEGKQAKVIENAEGARTTPSHVAFTKDGERLVGMPAKRQAVTNSAN      114
ScalHsc70-5     IGIDLGTTNSCVAVMEGKQAKVIENAEGARTTPSHVAFTKDGERLVGMPAKRQAVTNAAN      115
HsHSPA5         VGIDLGTTYSCVGVFKNGRVEIIANDQGNRITPSYVAFTPEGERLIGDAAKNQLTSNPEN      90
GmmHsc70-3      IGIDLGTTYSCVGVYKNGRVEIIANDQGNRITPSYVAFTADGERLIGDAAKNQLTTNPEN      91
DmelHsc70-3     IGIDLGTTYSCVGVYKNGRVEIIANDQGNRITPSYVAFTADGERLIGDAAKNQLTTNPEN      91
ScalHsc70-3     IGIDLGTTYSCVGVYKNGRVEIIANDQGNRITPSYVAFTADGERLIGDAAKNQLTTNPEN      91
DmelHsc70-4     VGIDLGTTYSCVGVFQHGKVEIIANDQGNRTTPSYVAFT-DTERLIGDAAKNQVAMNPTQ      65
GmmHsc70-4      VGIDLGTTYSCVGVFQHGKVEIIANDQGNRTTPSYVAFT-ETERLIGDAAKNQVAMNPQN      65
ScalHsc70-4     VGIDLGTTYSCVGVFQHGKVEIIANDQGNRTTPSYVAFT-DTERLIGDAAKNQVAMNPTN      65
DmelHsc70-1     VGIDLGTTYSCVGVFQHGKVEIIANDQGNRTTPSYVAFT-ESERLIGDAAKNQVAMNPNN      65
GmmHsc70-1      VGIDLGTTYSCVGVFQHGKVEIIANDQGNRVTPSYVAFT-DTERLIGDAAKNQVAMNPNN      65
ScalHsc70-1     ------------------------------------------------------------      0
HsHSPA6         VGIDLGTTYSCVGVFQQGRVEILANDQGNRTTPSYVAFT-DTERLVGDAAKSQAALNPHN      67
HsHSPA1A        IGIDLGTTYSCVGVFQHGKVEIIANDQGNRTTPSYVAFT-DTERLIGDAAKNQVALNPQN      65
HsHSPA1B        IGIDLGTTYSCVGVFQHGKVEIIANDQGNRTTPSYVAFT-DTERLIGDAAKNQVALNPQN      65
HsHSPA1L        IGIDLGTTYSCVGVFQHGKVEIIANDQGNRTTPSYVAFT-DTERLIGDAAKNQVAMNPQN      67
HsHSPA2         IGIDLGPTYSCVGVFQHGKVEIIANDQGNRTTPSYVAFT-DTERLIGDAAKNQVAMNPTN      66
HsHSPA8         VGIDLGTTYSCVGVFQHGKVEIIANDQGNRTTPSYVAFT-DTERLIGDAAKNQVAMNPTN      65
GmmHsp68        IGIDLGTTYSCVGVLQNGKVEIIANDQGNRTTPSYVAFT-DTERFIGSIAKNQGVMNPRN      62
DmelHsc70-2     IGIDLGTTYSCVGVWQNSKVEIIANDQGNRTTPSYVAFN-ETERLIGDPAKNQVAMNAKN      65
ScalHsc70-2     IGIDLGTTYSCVGVWQHNKVEIIANDQGNRTTPSYVAFN-DTERLIGDAAKNQVAMNAKN      65
DmelHsp70Aa     IGIDLGTTYSCVGVYQHGKVEIIANDQGNRTTPSYVAFT-DSERLIGDPAKNQVAMNPRN      62
DmelHsp70Ab     IGIDLGTTYSCVGVYQHGKVEIIANDQGNRTTPSYVAFT-DSERLIGDPAKNQVAMNPRN      62
DmelHsp70Ba     IGIDLGTTYSCVGVYQHGKVEIIANDQGNRTTPSYVAFT-DSERLIGDPAKNQVAMNPRN      62
DmelHsp70Bb     IGIDLGTTYSCVGVYQHGKVEIIANDQGNRTTPSYVAFT-DSERLIGDPAKNQVAMNPRN      62
DmelHsp70Bc     IGIDLGTTYSCVGVYQHGKVEIIANDQGNRTTPSYVAFT-DSERLIGDPAKNQVAMNPRN      62
DmelHsp70Bbb    IGIDLGTTYSCVGVYQHGKVEIIANDQGNRTTPSYVAFT-DSERLIGDPAKNQVAMNPRN      62
ScalHsp70       IGIDLGTTYSCVGVFQHGKVEIIANDQGNRTTPSYVAFS-ETERLIGDAAKNQVAMNPKN      62
DmelHsp68       IGIDLGTTYSCVGVFQYGKVEIIANDQGNRTTPSYVAFT-DSERLIGDAAKNQVAMNPKN      62
GmmHsp70A       IGIDLGTTYSCVGVFQHGKVEIIANDQGNRTTPSYVAFT-DSERLIGDAAKNQVAMNPKN      62
ScalHsp68       IGIDLGTTYSCVGVFQHGKVEIIANDQGNRTTPSYVAFS-DTERLIGDAAKNQVAMNPKN      62

EcHscC          TAALFKRAMGSNT----------NWR------------------LGSDTFNAPELSSLVL      99
HsHSPA9         TFYATKRLIGRRYDDPEVQKDIKNVPFKIVRASNG-DAWVEA---HGKLYSPSQIGAFVL      171
GmmHsc70-5      TFYATKRLIGRRFDDPEIKKDLKNLSYKVVKASNG-DAWVQS--SDGKVYSPSQIGAFIL      175
DmelHsc70-5     TFYATKRLIGRRFDDPEVKKDITNLSYKVVKASNG-DAWVSS--TDGKVYSPSQIGAFIL      171
ScalHsc70-5     TFYATKRLIGRRFDDPEIKKDLKNLSYKVTKASNG-DAWVQS--SDGKVYSPSQIGAFVL      172
```

```
HsHSPA5         TVFDAKRLIGRTWNDPSVQQDIKFLPFKVVEKKTKPYIQVDIGGGQTKTFAPEEISAMVL    150
GmmHsc70-3      TVFDAKRLIGREWSDINVQHDIKFFPFKVIEKNSKPHINVATSQG-NKVFAPEEISAMVL    150
DmelHsc70-3     TVFDAKRLIGREWSDTNVQHDIKFFPFKVVEKNSKPHISVDTSQG-AKVFAPEEISAMVL    150
ScalHsc70-3     TVFDAKRLIGREWTDTNVQHDIKYFPFKVVEKNSKPHISVDTSQG-KKVFAPEEISAMVL    150
DmelHsc70-4     TIFDAKRLIGRKFDDAAVQSDMKHWPFEVVSADGKPKIEVTYK-DEKKTFFPEEISSMVL    124
GmmHsc70-4      TIFDAKRLIGRKFDDPPVQADMKHWPFDVVNIDGKPKIQVIYK-DEKKTFFPEEISSMVL    124
ScalHsc70-4     TIFDAKRLIGRKFDDAPVQADMKHWPFDVVNVDSKPKIQVIYK-DEKKTFFPEEISSMVL    124
DmelHsc70-1     TIFDAKRLIGRRFDDATVQSDMKHWPFEVFAENGKPRIRVEYK-GERKSFYPEEVSSMVL    124
GmmHsc70-1      TIFDAKRLIGRKFDDPTVQSDMKHWPFEVISDSGKPKIRVEYK-GEKKTFFPEEVSSMVL    124
ScalHsc70-1     ----------------------MKHWPFEIVSDNGKPKIRVEYK-GEKKCFFPEEVSSMVL   38
HsHSPA6         TVFDAKRLIGRKFADTTVQSDMKHWPFRVVSEGGKPKVRVCYR-GEDKTFYPEEISSMVL    126
HsHSPA1A        TVFDAKRLIGRKFGDPVVQSDMKHWPFQVINDGDKPKVQVSYK-GETKAFYPEEISSMVL    124
HsHSPA1B        TVFDAKRLIGRKFGDPVVQSDMKHWPFQVINDGDKPKVQVSYK-GETKAFYPEEISSMVL    124
HsHSPA1L        TVFDAKRLIGRKFNDPVVQADMKLWPFQVINEGGKPKVLVSYK-GENKAFYPEEISSMVL    126
HsHSPA2         TIFDAKRLIGRKFGDATVQSDMKHWPFRVVSEGGKPKVQVEYK-GETKTFFPEEISSMVL    125
HsHSPA8         TVFDAKRLIGRRFDDAVVQSDMKHWPFMVVNDAGRPKVQVEYK-GETKSFYPEEVSSMVL    124
GmmHsp68        TIYDVKRLIGRKYNDSQIQEDIKNWPFKVISEGGKAKIWVEFK-GERKRFAPEEISSMIL    121
DmelHsc70-2     TVFDAKRLIGRKFDDKKIQEDLKLWPFKVINEKGKPKIEVEFK-GERKRFAPEEISSMVL    124
ScalHsc70-2     TVFDAKRLIGRKFDDQKIQEDMKHWPFQVINDCGKPKIEVEFK-GERKRFAPEEISSMVL    124
DmelHsp70Aa     TVFDAKRLIGRKYDDPKIAEDMKHWPFKVVSDGGKPKIGVEYK-GESKRFAPEEISSMVL    121
DmelHsp70Ab     TVFDAKRLIGRKYDDPKIAEDMKHWPFKVVSDGGKPKIGVEYK-GESKRFAPEEISSMVL    121
DmelHsp70Ba     TVFDAKRLIGRKYDDPKIAEDMKHWPFKVVSDGGKPKIGVEYK-GESKRFAPEEISSMVL    121
DmelHsp70Bb     TVFDAKRLIGRKYDDPKIAEDMKHWPFKVVSDGGKPKIGVEYK-GESKRFAPEEISSMVL    121
DmelHsp70Bc     TVFDAKRLIGRKYDDPKIAEDMKHWPFKVVSDGGKPKIGVEYK-GESKRFAPEEISSMVL    121
DmelHsp70Bbb    TVFDAKRLIGRKYDDPKIAEDMKHWPFKVVSDGGKPKIGVEYK-GESKRFAPEEISSMVL    121
ScalHsp70       TVFDAKRLIGRKYDDPKIMEDIKHWPFKVVSDCGKPMICVEFK-GEQKRFAPEEISSMVL    121
DmelHsp68       SVFDAKRLIGRRFDDSKIQEDIKHWPFKVINDNGKPKISVEFK-GANKCFSPEEISSMVL    121
GmmHsp70A       TVFDAKRLIGRKYDDSKIQEDIKHWPFKVISDGGKPKISVEFK-AEQKCFAPEEISSMVL    121
ScalHsp68       TVFDAKRLIGRKFEDPKIQDDMKHWPFKVMNDCGKPKICVEFK-GEQKRFAPEEISSMVL    121
                                                         . :   ::.:::*

EcHscC          RSLKEDAEEFLQRPIKDVVISVPAYFSDEQRKHTRLAAELAGLNAVRLINEPTAAAMAYG    159
HsHSPA9         MKMKETAENYLGHTAKNAVITVPAYFNDSQRQATKDAGQISGLNVLRVINEPTAAALAYG    231
GmmHsc70-5      MKMKETAEAYLNTKVKNAVITVPAYFNDSQRQATKDAGQIAGLNVLRVINEPTAAALAYG    235
DmelHsc70-5     MKMKETAEAYLNTPVKNAVVTVPAYFNDSQRQATKDAGQIAGLNVLRVINEPTAAALAYG    231
ScalHsc70-5     MKMRETAEAYLNTPVKNAVVTVPAYFNDSQRQATKDAGQIAGLNVLRVINEPTAAALAYG    232
HsHSPA5         TKMKETAEAYLGKKVTHAVVTVPAYFNDAQRQATKDAGTIAGLNVMRIINEPTAAAIAYG    210
GmmHsc70-3      GKMKETAEAYLGKKVTHAVVTVPAYFNDAQRQATKDAGVIAGLNVMRIINEPTAAAIAYG    210
DmelHsc70-3     GKMKETAEAYLGKKVTHAVVTVPAYFNDAQRQATKDAGVIAGLQVMRIINEPTAAAIAYG    210
ScalHsc70-3     LKMKETAEAYLGKKVTHAVVTVPAYFNDAQRQATKDAGVIAGLNVMRIINEPTAAAIAYG    210
DmelHsc70-4     TKMKETAEAYLGKTVTNAVITVPAYFNDSQRQATKDAGTIAGLNVLRIINEPTAAAIAYG    184
GmmHsc70-4      SKMKETAEAYLGKLVTNAVITVPAYFNDSQRQATKDAGTIAGLNVLRIINEPTAAAIAYG    184
ScalHsc70-4     SKMKETAEAYLGKTVTNAVITVPAYFNDSQRQATKDAGTIAGLNVLRIINEPTAAAIAYG    184
DmelHsc70-1     TKMRETAEAYLGGTVTDAVVTVPAYFNDSQRQATKDAGAIAGLNVLRIINEPTAAAIAYG    184
GmmHsc70-1      TKMKETAEAYLGKTVTDAVVTVPAYFNDSQRQATKDAGAIAGLNVLRIINEPTAAAIAYG    184
ScalHsc70-1     TKMKETAEAYLGRTVTDAVVTVPAYFNDSQRQATKDAGAIAGLNVLRIINEPTAAAIAYG    98
HsHSPA6         SKMKETAEAYLGQPVKHAVITVPAYFNDSQRQATKDAGAIAGLNVLRIINEPTAAAIAYG    186
HsHSPA1A        TKMKEIAEAYLGYPVTNAVITVPAYFNDSQRQATKDAGVIAGLNVLRIINEPTAAAIAYG    184
HsHSPA1B        TKMKEIAEAYLGYPVTNAVITVPAYFNDSQRQATKDAGVIAGLNVLRIINEPTAAAIAYG    184
HsHSPA1L        TKLKETAEAFLGHPVTNAVITVPAYFNDSQRQATKDAGVIAGLNVLRIINEPTAAAIAYG    186
HsHSPA2         TKMKEIAEAYLGGKVHSAVITVPAYFNDSQRQATKDAGTITGLNVLRIINEPTAAAIAYG    185
HsHSPA8         TKMKEIAEAYLGKTVTNAVVTVPAYFNDSQRQATKDAGTIAGLNVLRIINEPTAAAIAYG    184
GmmHsp68        TRMKETAEAYLGHTVKDAVVTVPAYFNDSQRQATKNAGTIAGLNILRIINEPTSAALAYG    181
DmelHsc70-2     TKMREIAEVYLGGKVKDAVVTVPAYFNDSQRQATKDAGSIAGLNVLRIINEPTAAALAYG    184
ScalHsc70-2     SKMKEVAEVYLGGQVTDAVITVPAYFNDSQRQATKDAGSIAGLNVLRIINEPTAAALAYG    184
DmelHsp70Aa     TKMKETAEAYLGESITDAVITVPAYFNDSQRQATKDAGHIAGLNVLRIINEPTAAALAYG    181
DmelHsp70Ab     TKMKETAEAYLGESITDAVITVPAYFNDSQRQATKDAGHIAGLNVLRIINEPTAAALAYG    181
DmelHsp70Ba     TKMKETAEAYLGESITDAVITVPAYFNDSQRQATKDAGHIAGLNVLRIINEPTAAALAYG    181
DmelHsp70Bb     TKMKETAEAYLGESITDAVITVPAYFNDSQRQATKDAGHIAGLNVLRIINEPTAAALAYG    181
DmelHsp70Bc     TKMKETAEAYLGESITDAVITVPAYFNDSQRQATKDAGHIAGLNVLRIINEPTAAALAYG    181
DmelHsp70Bbb    TKMKETAEAYLGESITDAVITVPAYFNDSQRQATKDAGHIAGLNVLRIINEPTAAALAYG    181
ScalHsp70       TKMKETAEAYLGQSVSDAVITVPAYFNDSQRQATKDAGRIAGLNVLRIINEPTAAALAYG    181
DmelHsp68       TKMKETAEAYLGTTVKDAVITVPAYFNDSQRQATKDAGAIAGINVLRIINEPTAAALAYG    181
GmmHsp70A       TKMKETAEAYLGHTVKDAVVTVPAYFNDSQRQATKDAGAIAGLNVLRIINEPTAAALAYG    181
ScalHsp68       TKMKETAESYLGTTIRDAVITVPAYFNDSQRQATKDAGAIAGLNVLRIINEPTAAALAYG    181
                  ::* ** :*      .*::*****.* **: *: *. ::*:: :*:*****:**:***

EcHscC          LHTQQ----NTRSLVFDLGGGTFDVTVLEYATP-VIEVNASAGDNFLGGEDFTHMLVDEV    214
HsHSPA9         LDKSE----DKVIAVYDLGGGTFDISILEIQKG-VFEVKSTNGDTFLGGEDFDQALLRHI    286
GmmHsc70-5      MDKTD----DKIIAVYDLGGGTFDISILEIQKG-VFEVKSTNGDTMLGGEDFDNAIVDFL    290
DmelHsc70-5     MDKTE----DKIIAVYDLGGGTFDISILEIQKG-VFEVKSTNGDTLLGGEDFDNHIVNFL    286
ScalHsc70-5     MDKTE----DKIIAVYDLGGGTFDISILEIQKG-VFEVKSTNGDTMLGGEDFDNAIVNYL    287
HsHSPA5         LDKR---EGEKNILVFDLGGGTFDVSLLTIDNG-VFEVVATNGDTHLGGEDFDQRVMEHF    266
GmmHsc70-3      LDKK---EGEKNVLVFDLGGGTFDVSLLTIDNG-VFEVVATNGDTHLGGEDFDQRVMDHF    266
DmelHsc70-3     LDKK---EGEKNVLVFDLGGGTFDVSLLTIDNG-VFEVVATNGDTHLGGEDFDQRVMDHF    266
ScalHsc70-3     LDKK---EGEKNVLVFDLGGGTFDVSLLTIDNG-VFEVVSTNGDTHLGGEDFDQRVMDHF    266
DmelHsc70-4     LDKKA--VGERNVLIFDLGGGTFDVSILSIDDG-IFEVKSTAGDTHLGGEDFDNRLVTHF    241
```

```
GmmHsc70-4        LDKKA--AGERNVLIFDLGGGTFDVSILSIDDG-IFEVKSTAGDTHLGGEDFDNRLVTHF        241
ScalHsc70-4       LDKKA--IGERNVLIFDLGGGTFDVSILSIDDG-IFEVKSTAGDTHLGGEDFDNRLVTHF        241
DmelHsc70-1       LDKQG--TSERNVLIFDLGGGTFDVSVLTIEDG-IFEVKATAGDTHLGGEDFDNRLVNHF        241
GmmHsc70-1        LDKKG--THERNVLIFDLGGGTFDVSILTIEDG-IFEVKSTAGDTHLGGEDFDNRMVNHF        241
ScalHsc70-1       LDKKG--TSERNVLIFDLGGGTFDVSILTIEDG-IFEVKSTAGDTHLGGEDFDNRMVNHF        155
HsHSPA6           LDRRG--AGERNVLIFDLGGGTFDVSVLSIDAG-VFEVKATAGDTHLGGEDFDNRLVNHF        243
HsHSPA1A          LDRTG--KGERNVLIFDLGGGTFDVSILTIDDG-IFEVKATAGDTHLGGEDFDNRLVNHF        241
HsHSPA1B          LDRTG--KGERNVLIFDLGGGTFDVSILTIDDG-IFEVKATAGDTHLGGEDFDNRLVNHF        241
HsHSPA1L          LDKGG--QGERHVLIFDLGGGTFDVSILTIDDG-IFEVKATAGDTHLGGEDFDNRLVSHF        243
HsHSPA2           LDKKGCAGGEKNVLIFDLGGGTFDVSILTIEDG-IFEVKSTAGDTHLGGEDFDNRMVSHL        244
HsHSPA8           LDKK--VGAERNVLIFDLGGGTFDVSILTIEDG-IFEVKSTAGDTHLGGEDFDNRMVNHF        241
GmmHsp68          LDKNL--KGERNALIFDLGGGTFDVSILTIDKDSLFEVRATAANAYLGGEDFDSRLVNHL        239
DmelHsc70-2       LDKNL--KGERNVLIFDLGGGTFDVSVLSIDEGSLFEVKATAGDTHLGGEDFDNRLVNHF        242
ScalHsc70-2       LDKNL--KGEKNVLIFDLGGGTFDVSILTIDEGSLFEVKSTAGDTHLGGEDFDNRLVNFF        242
DmelHsp70Aa       LDKNL--KGERNVLIFDLGGGTFDVSILTIDEGSLFEVRSTAGDTHLGGEDFDNRLVTHL        239
DmelHsp70Ab       LDKNL--KGERNVLIFDLGGGTFDVSILTIDEGSLFEVRSTAGDTHLGGEDFDNRLVTHL        239
DmelHsp70Ba       LDKNL--KGERNVLIFDLGGGTFDVSILTIDEGSLFEVRSTAGDTHLGGEDFDNRLVTHL        239
DmelHsp70Bb       LDKNL--KGERNVLIFDLGGGTFDVSILTIDEGSLFEVRSTAGDTHLGGEDFDNRLVTHL        239
DmelHsp70Bc       LDKNL--KGERNVLIFDLGGGTFDVSILTIDEGSLFEVRSTAGDTHLGGEDFDNRLVTHL        239
DmelHsp70Bbb      LDKNL--KDERNVLIFDLGGGTFDVSILTIDEGSLFEVRSTAGDTHLGGEDFDNRLVTHL        239
ScalHsp70         LDKNL--KGERNVLIFDLGGGTFDVSILTIDEGSLFEVRATAGDTHLGGEDFDNRLVTHL        239
DmelHsp68         LDKNL--KGERNVLIFDLGGGTFDVSILTIDEGSLFEVRSTAGDTHLGGEDFDNRLVNHF        239
GmmHsp70A         LDKNL--KGERNVLIFDLGGGTFDVSILTIDEGSLFEVRATAGDTHLGGEDFDSRLVNHF        239
ScalHsp68         LDKNL--KGERNVLIFDLGGGTFDVSILTIDEGSLFEVKATAGDTHLGGEDFDNRLVNHF        239
                  :.       :    ::********:::*      ::** :: .:  ******   ::  .

EcHscC            LKR---AEVAKTTLNDSELAALYACVEAAKCSN----QSPMHIRWQYQDEMRKCEFYE--        265
HsHSPA9           VKEFKRETGVDLTKDNMALQRVREAAEKAKCELSSSVQTDINLPYLTMDSSGPKHLNMKL        346
GmmHsc70-5        VSEFKRDTGIDITKDSIAMQRLKEAAEKAKCELSSSHQTDINLPYLTMDTSGPQHMNLKM        350
DmelHsc70-5       VAEFKKDSGIDIRKDNIAMQRLKEAAEKAKCELSSSQQTDINLPYLTMDAAGPQHMNLKL        346
ScalHsc70-5       VAEFKKGSGIDITKDSIAMQRLKEAAEKAKCELSSSQQTDINLPYLTMDAAGPQHMNLKM        347
HsHSPA5           IKLYKKKTGKDVRKDNRAVQKLRREVEKAKRALSSQHQARIEIESFYE----GEDFSETL        322
GmmHsc70-3        IKLYKKKKGKDIRKDNRAVQKLRREVEKAKRALSSAHQVRLEIESFFE----GEDFSETL        322
DmelHsc70-3       IKLYKKKKGKDIRKDNRAVQKLRREVEKAKRALSGSHQVRIEIESFFE----GDDFSETL        322
ScalHsc70-3       IKLYKKKKGKDIRKDNRAVQKLRREVEKAKRALSSAHQVRIEIESFFE----GDDFSETL        322
DmelHsc70-4       VQEFKRKHKKDLTTNKRALRRLRTACERAKRTLSSSTQASIEIDSLFE----GTDFYTSI        297
GmmHsc70-4        VQEFKRKHKKDLTANKRALRRLRTACERAKRTLSSSTQASIEIDSLFE----GTDFYTSI        297
ScalHsc70-4       VQEFKRKHKKDLASNKRALRRLRTACERAKRTLSSSTQASIEIDSLFE----GTDFYTSI        297
DmelHsc70-1       VQEFQRKHKKDLGQNKRALRRLRTACERAKRTLSSSTQASIEIDSLFE----GVDFYTSV        297
GmmHsc70-1        VQEFQRKYKKDLSSNKRAVRRLRTACERAKRTLSASSQASIEIDSLFD----GIDFYTSI        297
ScalHsc70-1       VQEFQRKHKKDLSANKRALRRLRTACERAKRTLSASSQASIEIDSLLD----GIDFYTSI        211
HsHSPA6           MEEFRRKHGKDLSGNKRALRRLRTACERAKRTLSSSTQATLEIDSLFE----GVDFYTSI        299
HsHSPA1A          VEEFKRKHKKDISQNKRAVRRLRTACERAKRTLSSSTQASLEIDSLFE----GIDFYTSI        297
HsHSPA1B          VEEFKRKHKKDISQNKRAVRRLRTACERAKRTLSSSTQASLEIDSLFE----GIDFYTSI        297
HsHSPA1L          VEEFKRKHKKDISQNKRAVRRLRTACERAKRTLSSSTQANLEIDSLYE----GIDFYTSI        299
HsHSPA2           AEEFKRKHKKDIGPNKRAVRRLRTACERAKRTLSSSTQASIEIDSLYE----GVDFYTSI        300
HsHSPA8           IAEFKRKHKKDISENKRAVRRLRTACERAKRTLSSSTQASIEIDSLYE----GIDFYTSI        297
GmmHsp68          VDEFRRKYNMDLKSNRRVLRRLRTVAERAKRTLSSTTEALVKVNALFA----GIDFYTKV        295
DmelHsc70-2       AEEFKRKHKSDIKGNARALRRLRTACERAKRTLSSSTEASLEIDALHE----GIDFYSKI        298
ScalHsc70-2       VEEFKRKHKRDMSSNVRALRRLRTACERAKRTLSSSSEASLEIDALHE----GIDFYSKI        298
DmelHsp70Aa       ADEFKRKYKKDLRSNPRALRRLRTAAERAKRTLSSSTEATIEIDALFE----GQDFYTKV        295
DmelHsp70Ab       ADEFKRKYKKDLRSNPRALRRLRTAAERAKRTLSSSTEATIEIDALFE----GQDFYTKV        295
DmelHsp70Ba       AEEFKRKYKKDLRSNPRALRRLRTAAERAKRTLSSSTEATIEIDALFE----GQDFYTKV        295
DmelHsp70Bb       AEEFKRKYKKDLRSNPRALRRLRTAAERAKRTLSSSTEATIEIDALFE----GQDFYTKV        295
DmelHsp70Bc       AEEFKRKYKKDLRSNPRALRRLRTAAERAKRTLSSSTEATIEIDALFE----GQDFYTKV        295
DmelHsp70Bbb      AEEFKRKYKKDLRSNPRALRRLRTAAERAKRTLSSSTEATIEIDALFE----GQDFYTKV        295
ScalHsp70         AEEFKRKYKKDLRSNPRALRRLRTAAERAKRTLSSSSEATIEIDALFE----GQDFYTKI        295
DmelHsp68         AEEFKRKYKKDLRSNPRALRRLRTAAERAKRTLSSSTEASLEIDALYE----GHDFYSKV        295
GmmHsp70A         ADEFQRKYKKDLRSNPRALRRLRTAAERAKRTLSSSTEATLEIDAIFE----GIDFYTKV        295
ScalHsp68         AEEFKRKYKKDMRSNPRALRRLRSAAERAKRTLSSSTEATIEVDALFE----GIDFYTKI        295
                              .   :   :  :     * **       :  :.:            .:

EcHscC            --NELEDLWLPLLNRLRVPIEQALRDARLKPSQIDSLVLVGGASQMPLVQRIAVRLF-GK        322
HsHSPA9           TRAQFEGIVTDLIRRTIAPCQKAMQDAEVSKSDIGEVILVGGMTRMPKVQQTVQDLF-GR        405
GmmHsc70-5        TRSKLESLVGELIKRTIQPCQKALSDAEVSKSDIGEVLLVGGMTRMPKVHSTVQELF-GR        409
DmelHsc70-5       TRSKLESLVGDLIKRTIQPCQKALSDAEVSKSEIGEVLLVGGMTRMPKVQSTVQELF-GR        405
ScalHsc70-5       TRSKLESLVGDLIKRTIQPCQKALSDAEVNKSDIGEVLLVGGMTRMPKVQQTVQDLF-GR        406
HsHSPA5           TRAKFEELNMDLFRSTMKPVQKVLEDSDLKKSDIDEIVLVGGSTRIPKIQQLVKEFFNGK        382
GmmHsc70-3        TRAKFEELNMDLFRSTLKPVQKVLEDADMNKKDVHEIVLVGGSTRIPKVQQLVKEFFGGK        382
DmelHsc70-3       TRAKFEELNLDLFRSTLKPVQKVLEDADMNKKDVHEIVLVGGSTRIPKVQQLVKDFFGGK        382
ScalHsc70-3       TRAKFEELNMDLFRSTLKPVQKVLEDADMNKKDVHEIVLVGGSTRIPKVQQLVKDFFGGK        382
DmelHsc70-4       TRARFEELNADLFRSTMDPVEKALRDAKLDKSVIHDIVLVGGSTRIPKVQRLLQDLFNGK        357
GmmHsc70-4        TRARFEELNADLFRSTMDPVEKALRDAKLDKSQIHDIVLVGGSTRIPKVQKLLQDLFNGK        357
ScalHsc70-4       TRARFEELNADLFRSTMDPVEKALRDAKLDKAQIHDIVLVGGSTRIPKVQKLLQDFFNGK        357
DmelHsc70-1       TRARFEELNGDLFRGTMEPVAKALRDAKMDKGQIHDIVLVGGSTRIPKVQRLLQDFFNGK        357
GmmHsc70-1        TRARFEELNGDLFRGTMEPVAKSLRDAKMDKGQVHDIVLVGGSTRIPKVQKLLQDFFNGK        357
ScalHsc70-1       TRARFEELNGDLFRGTMDPVAKALRDAKMDKGQINDIVLVGGSTRIPKVQKLLQDFFNGK        271
```

```
HsHSPA6           TRARFEELCSDLFRSTLEPVEKALRDAKLDKAQIHDVVLVGGSTRIPKVQKLLQDFFNGK     359
HsHSPA1A          TRARFEELCSDLFRSTLEPVEKALRDAKLDKAQIHDLVLVGGSTRIPKVQKLLQDFFNGR     357
HsHSPA1B          TRARFEELCSDLFRSTLEPVEKALRDAKLDKAQIHDLVLVGGSTRIPKVQKLLQDFFNGR     357
HsHSPA1L          TRARFEELCADLFRGTLEPVEKALRDAKMDKAKIHDIVLVGGSTRIPKVQRLLQDYFNGR     359
HsHSPA2           TRARFEELNADLFRGTLEPVEKALRDAKLDKGQIQEIVLVGGSTRIPKIQKLLQDFFNGK     360
HsHSPA8           TRARFEELNADLFRGTLDPVEKALRDAKLDKSQIHDIVLVGGSTRIPKIQKLLQDFFNGK     357
GmmHsp68          SRAHFEELCADLFCQTLITVEKALNEAKMDRNEIHDIVLVGGSTRIPKVQQLLQQFFSGK     355
DmelHsc70-2       SRARFEELNMDLFRSTMQPVERALSDAKMDKKAIHDVVLVGGSTRIPKIQKLLQDLFGGK     358
ScalHsc70-2       TRARFEELNMDLFRSTLMPVEKALNDAKMDKSSINDVVLVGGSTRIPKIQKMLQDFFGGK     358
DmelHsp70Aa       SRARFEELCADLFRNTLQPVEKALNDAKMDKGQIHDIVLVGGSTRIPKVQSLLQDFFHGK     355
DmelHsp70Ab       SRARFEELCADLFRNTLQPVEKALNDAKMDKGQIHDIVLVGGSTRIPKVQSLLQDFFHGK     355
DmelHsp70Ba       SRARFEELCADLFRNTLQPVEKALNDAKMDKGQIHDIVLVGGSTRIPKVQSLLQEFFHGK     355
DmelHsp70Bb       SRARFEELCADLFRNTLQPVEKALNDAKMDKGQIHDIVLVGGSTRIPKVQSLLQEFFHGK     355
DmelHsp70Bc       SRARFEELCADLFRNTLQPVEKALNDAKMDKGQIHDIVLVGGSTRIPKVQSLLQEFFHGK     355
DmelHsp70Bbb      SRARFEELCADLFRNTLQPVEKALNDAKMDKGQIHDIVLVGGSTRIPKVQSLLQEFFHGK     355
ScalHsp70         SRARFEELCADLFRQTLQPVEKALNDAKMDKSDIHDIVLVGGSTRIPKVQNLLQQFFCGK     355
DmelHsp68         SRARFEELCGDLFRNTLEPVEKALKDAKMDKSQIHDIVLVGGSTRIPKVQNLLQNFFGGK     355
GmmHsp70A         SRARFEELCADLFRQTLQPVEKALNDAKMDKNQIHDIVMVGGSTRIPKVQNLLQQFFCGK     355
ScalHsp68         SRARFEELCSDLFRNTLQPVEKALNDAKMDKNQIHDIVLVGGSTRIPKVQNLLQQFFCGK     355
                     .:* :   *:        : : :: :.   : .:::*** :::* ::      * *:

EcHscC            LPYQSYDPSTIVALGAAIQAACRLRN--EDIEEVILTDICPYSLGVEVNRQGVSGIFSPI     380
HsHSPA9           APSKAVNPDEAVAIGAAIQGGVLAGD----VTDVLLLDVTPLSLGIET----LGGVFTKL     457
GmmHsc70-5        QPSRSVNPDEAVAVGAAVQGGVLAGD----VTDVLLLDVTPLSLGIET----LGGVFTRL     461
DmelHsc70-5       QPSRSVNPDEAVAVGAAVQGGVLAGD----VTDVLLLDVTPLSLGIET----LGGVFTRL     457
ScalHsc70-5       QPSRSVNPDEAVAVGAAVQGGVLAGD----VTDVLLLDVTPLSLGIET----LGGVFTRL     458
HsHSPA5           EPSRGINPDEAVAYGAAVQAGVLSGDQD--TGDLVLLDVCPLTLGIET----VGGVMTKL     436
GmmHsc70-3        EPSRGINPDEAVAYGAAVQAGVLSGEQD--TDAIVLLDVNPLTLGIET----VGGVMTKL     436
DmelHsc70-3       EPSRGINPDEAVAYGAAVQAGVLSGEQD--TDAIVLLDVNPLTMGIET----VGGVMTKL     436
ScalHsc70-3       EPSRGINPDEAVAYGAAVQAGVLSGEQD--TDAIVLLDVNPLTMGIET----VGGVMTKL     436
DmelHsc70-4       ELNKSINPDEAVAYGAAVQAAILHGDKSQEVQDLLLLDVTPLSLGIET----AGGVMSVL     413
GmmHsc70-4        ELNKSINPDEAVAYGAAVQAAILHGDKSQEVQDLLLLDVTPLSLGIET----AGGVMSVL     413
ScalHsc70-4       ELNKSINPDEAVAYGAAVQAAILHGDKSQEVQDLLLLDVTPLSLGIET----AGGVMSVL     413
DmelHsc70-1       ELNKSINPDEAVAYGAAVQAAILHGDKSEAVQDLLLLDVTPLSLGIET----AGGVMTTL     413
GmmHsc70-1        ELNKSINPDEAVAYGAAVQAAILHGDKSEAVQDLLLLDVTPLSLGIET----AGGVMTVL     413
ScalHsc70-1       DLNKSINPDEAVAYGAAVQAAILHGDKSEAVQDLLLLDVTPLSLGIET----AGGVMTVL     327
HsHSPA6           ELNKSINPDEAVAYGAAVQAAVLMGDKCEKVQDLLLLDVAPLSLGLET----AGGVMTTL     415
HsHSPA1A          DLNKSINPDEAVAYGAAVQAAILMGDKSENVQDLLLLDVAPLSLGLET----AGGVMTAL     413
HsHSPA1B          DLNKSINPDEAVAYGAAVQAAILMGDKSENVQDLLLLDVAPLSLGLET----AGGVMTAL     413
HsHSPA1L          DLNKSINPDEAVAYGAAVQAAILMGDKSEKVQDLLLLDVAPLSLGLET----AGGVMTAL     415
HsHSPA2           ELNKSINPDEAVAYGAAVQAAILIGDKSENVQDLLLLDVTPLSLGIET----AGGVMTPL     416
HsHSPA8           ELNKSINPDEAVAYGAAVQAAILSGDKSENVQDLLLLDVTPLSLGIET----AGGVMTVL     413
GmmHsp68          KLTMSINPDEAVAYGAAIQAAILNGDTSDNIQDVLLVDVTPLSLGIET----AAGIVTKI     411
DmelHsc70-2       QLNLSINPDEAVAYGAAVQAAILTGVGSSQIQDLLLVDVAPLSLGIET----AGGVMTNL     414
ScalHsc70-2       QLNLSINPDEAVAYGAAVQAAILTGVGSSQIQDVLLVDVTPLSLGIET----AGGVMTKL     414
DmelHsp70Aa       NLNLSINPDEAVAYGAAVQAAILSGDQSGKIQDVLLVDVAPLSLGIET----AGGVMTKL     411
DmelHsp70Ab       NLNLSINPDEAVAYGAAVQAAILSGDQSGKIQDVLLVDVAPLSLGIET----AGGVMTKL     411
DmelHsp70Ba       NLNLSINPDEAVAYGAAVQAAILSGDQSGKIQDVLLVDVAPLSLGIET----AGGVMTKL     411
DmelHsp70Bb       NLNLSINPDEAVAYGAAVQAAILSGDQSGKIQDVLLVDVAPLSLGIET----AGGVMTKL     411
DmelHsp70Bc       NLNLSINPDEAVAYGAAVQAAILSGDQSGKIQDVLLVDVAPLSLGIET----AGGVMTKL     411
DmelHsp70Bbb      NLNLSINPDEAVAYGAAVQAAILSGDQSGKIQDVLLVDVAPLSLGIET----AGGVMTKL     411
ScalHsp70         SLNLSINPDEAVAYGAAIQAAILSGDKSSAIQDVLLVDVAPLSLGIET----AGGVMAKI     411
DmelHsp68         TLNLSINPDEAVAYGAAIQAAILSGDKSSEIKDVLLVDVAPLSLGIET----AGGVMTKL     411
GmmHsp70A         SLNLSINPDEAVAYGAAIQAAILSGDKSSEIQDVLLVDVAPLSLGIET----AGGVMTKI     411
ScalHsp68         SLNLSINPDEAVAYGAAIQAAILSGDKSSVIQDVLLVDVAPLSLGIET----AGGVMTKL     411
                      . :*.  ** ***:*..            ::* *: * ::*:*.     .*:.: :

EcHscC            IERNTTVPVSRVETYSTMHPEQDSITVNVYQGENHKVKNNILVESFDVPLKKTGT--YQS     438
HsHSPA9           INRNTTIPTKKSQVFSTAADGQTQVEIKVCQGEREMAGDNKLLGQFTLIGIPPAPRGVPQ     517
GmmHsc70-5        ISRNTTIPTKKSQVFSTAADGQTQVEIKVHQGEREMAGDNKLLGAFTLVGIPPAPRGVPQ     521
DmelHsc70-5       ISRNTTIPTKKSQVFSTASDGQTQVEIKVHQGEREMANDNKLLGSFTLVGIPPAPRGVPQ     517
ScalHsc70-5       ITRNTTIPTKKSQVFSTAADGQTQVEIKVHQGEREMAADNKKLGSFTLVGIPPAPRGVPQ     518
HsHSPA5           IPRNTVVPTKKSQIFSTASDNQPTVTIKVYEGERPLTKDNHLLGTFDLTGIPPAPRGVPQ     496
GmmHsc70-3        IPRNTVIPTKKSQIFSTASDNQHTVTIQVYEGERPMTKDNHLLGKFDLTGIPPAPRGIPQ     496
DmelHsc70-3       IPRNTVIPTKKSQVFSTASDNQHTVTIQVYEGERPMTKDNHLLGKFDLTGIPPAPRGIPQ     496
ScalHsc70-3       IPRNTVIPTKKSQIFSTASDNQHTVTIQVYEGERPMTKDNHLLGKFDLTGIPPAPRGIPQ     496
DmelHsc70-4       IKRNTTIPTKQTQTFTTYSDNQPGVLIQVYEGERAMTKDNNLLGKFELSGIPPAPRGVPQ     473
GmmHsc70-4        IKRNTTIPTKQTQTFTTYSDNQPGVLIQVYEGERAMTKDNNLLGKFELSGIPPAPRGVPQ     473
ScalHsc70-4       IKRNTTIPTKQTQTFTTYSDNQPGVLIQVYEGERAMTKDNNLLGKFELSGIPPAPRGVPQ     473
DmelHsc70-1       IKRNTTIPTKQTQIFTTYADNQPGVLIQVFEGERAMTRDNNSLGKFELSAIPPAPRGVPQ     473
GmmHsc70-1        INRNTTIPTKQTQVFTTYSDNQPGVLIQVFEGERAMTKDNNILGKFELSGIPPAPRGVPQ     473
ScalHsc70-1       IKRNTTIPTKQTQVFTTYSDNQPGVLIQVFEGERAMTKDNNILGKFELSGIPPAPRGVPQ     387
HsHSPA6           IQRNATIPTKQTQTFTTYSDNQPGVFIQVYEGERAMTKDNNLLGRFELSGIPPAPRGVPQ     475
HsHSPA1A          IKRNSTIPTKQTQIFTTYSDNQPGVLIQVYEGERAMTKDNNLLGRFELSGIPPAPRGVPQ     473
HsHSPA1B          IKRNSTIPTKQTQIFTTYSDNQPGVLIQVYEGERAMTKDNNLLGRFELSGIPPAPRGVPQ     473
HsHSPA1L          IKRNSTIPTKQTQIFTTYSDNQPGVLIQVYEGERAMTKDNNLLGRFDLTGIPPAPRGVPQ     475
HsHSPA2           IKRNTTIPTKQTQTFTTYSDNQSSVLVQVYEGERAMTKDNNLLGKFDLTGIPPAPRGVPQ     476
```

```
HsHSPA8          IKRNTTIPTKQTQTFTTYSDNQPGVLIQVYEGERAMTKDNNLLGKFELTGIPPAPRGVPQ      473
GmmHsp68         TERNNRILCKQRKTLITYADNQPTVAIQVFEDERVMTKENKLLGVFNLTGLPSGSRDIPK      471
DmelHsc70-2      VERNARIPCKQQQIFTTYSDNQNAVTIQVYEGERAMTKDNNLLGTFNLTGIPPAPRGVPQ      474
ScalHsc70-2      IERNARIPCKQQQTFTTYSDNQNAVTIQVYEGERAMTKDNNLLGTFNLTGIPPAPRGVPK      474
DmelHsp70Aa      IERNCRIPCKQTKTFSTYADNQPGVSIQVYEGERAMTKDNNALGTFDLSGIPPAPRGVPQ      471
DmelHsp70Ab      IERNCRIPCKQTKTFSTYADNQPGVSIQVYEGERAMTKDNNALGTFDLSGIPPAPRGVPQ      471
DmelHsp70Ba      IERNCRIPCKQTKTFSTYSDNQPGVSIQVYEGERAMTKDNNALGTFDLSGIPPAPRGVPQ      471
DmelHsp70Bb      IERNCRIPCKQTKTFSTYSDNQPGVSIQVYEGERAMTKDNNALGTFDLSGIPPAPRGVPQ      471
DmelHsp70Bc      IERNCRIPCKQTKTFSTYSDNQPGVSIQVYEGERAMTKDNNALGTFDLSGIPPAPRGVPQ      471
DmelHsp70Bbb     IERNCRIPCKQTKTFSTYSDNQPGVSIQVYEGERAMTKDNNALGTFDLSGIPPAPRGVPQ      471
ScalHsp70        IERNSRIPCKQTQTFSTYSDNQSGVTIQVFEGERVMTRDNNRLGTFDLSGIPPAPRGVPQ      471
DmelHsp68        IERNSRIPCKQSKTFTTYADNQPAVTIQVFEGERALTKDNNVLGTFDLTGVPPAPRGVPK      471
GmmHsp70A        IERNSRIPCKQSKTFTTYADNQPAVTIQVFEGERTMTKDNNLLGTFNLTGIPPAPRGVPK      471
ScalHsp68        IERNSRIPCKQSKTFTTYADNQPAVTIQVFEGERAMTKDNNLLGTFNLTGIPPAPRGVPK      471
                   **  :  .: :   *    *  : ::* :.*.  . :*  :  * :      .     .

EcHscC           I--DIRFSYDINGLLEVDVLLED-GSVKSRVINHSPVTLSAQQIEESRTRLSA-------      488
HsHSPA9          IE--VTFDIDANGIVHVSAKDKGTGREQQIVIQS-SGGLSKDDIENMVKNAEKYAEEDRR      574
GmmHsc70-5       IE--VVFDIDANGIVHVSAKDKGTGREQQIVIQS-SGGLSKDEIENMIKKAEEYATADKK      578
DmelHsc70-5      IE--VVFDIDANGIVHVSAKDKGTGKEQQIVIQS-SGGLSKDEIENMIKKAEEYATADKQ      574
ScalHsc70-5      IE--VVFDIDANGIVHVSAKDKGTGKEQQIVIQS-SGGLSKDEIENMVKKAEEMAAQDKK      575
HsHSPA5          IE--VTFEIDVNGILRVTAEDKGTGNKNKITITNDQNRLTPEEIERMVNDAEKFAEEDKK      554
GmmHsc70-3       IE--VSFEIDANGILQVSAEDKGTGNKEKIVITNDQNRLTPEDIERMIHDAEKFADEDKK      554
DmelHsc70-3      IE--VSFEIDANGILQVSAEDKGTGNKEKIVITNDQNRLTPEDIDRMIRDAEKFADEDKK      554
ScalHsc70-3      IE--VSFEIDANGILQVSAEDKGTGNKEKIVITNDQNRLTPEDIERMIHDAEKFADEDKK      554
DmelHsc70-4      IE--VTFDIDANGILNVTALERSTNKENKITITNDKGRLSKEDIERMVNEAEKYRNEDEK      531
GmmHsc70-4       IE--VTFDIDANGILNVTALERSTNKENKITITNDKGRLSKEDIERMVNEAEKYRSEDEK      531
ScalHsc70-4      IE--VTFDIDANGILNVTALERSTNKENKITITNDKGRLSKEDIERMVNEAEKYRNEDEK      531
DmelHsc70-1      VE--VTFDIDANGILNVTALEKSTGKENRITITNDKGRLSKEDIERMVNDAEAYRQADEQ      531
GmmHsc70-1       IE--VTFDIDANGILNVTAIEKSTGKENKITITNDKGRLSKEDIERMVNEAEQYRNEDEK      531
ScalHsc70-1      IE--VTFDIDANGILNVTAVEKSTNKENKITITNDKGRLSKEDIERMVNDAEAYRHEDEK      445
HsHSPA6          IE--VTFDIDANGILSVTATDRSTGKANKITITNDKGRLSKEEVERMVHEAEQYKAEDEA      533
HsHSPA1A         IE--VTFDIDANGILNVTATDKSTGKANKITITNDKGRLSKEEIERMVQEAEKYKAEDEV      531
HsHSPA1B         IE--VTFDIDANGILNVTATDKSTGKANKITITNDKGRLSKEEIERMVQEAEKYKAEDEV      531
HsHSPA1L         IE--VTFDIDANGILNVTATDKSTGKVNKITITNDKGRLSKEEIERMVLDAEKYKAEDEV      533
HsHSPA2          IE--VTFDIDANGILNVTAADKSTGKENKITITNDKGRLSKDDIDRMVQEAERYKSEDEA      534
HsHSPA8          IE--VTFDIDANGILNVSAVDKSTGKENKITITNDKGRLSKEDIERMVQEAEKYKAEDEK      531
GmmHsp68         ININITFHLDAKCILNVTAKKRSTGVLKKVTINNDRNRLSQAEIDRMVRKAEQHADEDEK      531
DmelHsc70-2      IE--VAFDLNADGILNVNAKDNSTGKSEKITISNDKGRLSKAEIDRMLSEAEKYKVEDDR      532
ScalHsc70-2      IE--VTFDLNADGILNVSAKDNSTGKSEKITITNDKGRLSKADIDRMLSEAEKYKDDDEK      532
DmelHsp70Aa      IE--VTFDLDANGILNVSAKEMSTGKAKNITIKNDKGRLSQAEIDRMVNEAEKYADEDEK      529
DmelHsp70Ab      IE--VTFDLDANGILNVSAKEMSTGKAKNITIKNDKGRLSQAEIDRMVNEAEKYADEDEK      529
DmelHsp70Ba      IE--VTFDLDANGILNVSAKEMSTGKAKNITIKNDKGRLSQAEIDRMVNEAEKYADEDEK      529
DmelHsp70Bb      IE--VTFDLDANGILNVSAKEMSTGKAKNITIKNDKGRLSQAEIDRMVNEAEKYADEDEK      529
DmelHsp70Bc      IE--VTFDLDANGILNVSAKEMSTGKAKNITIKNDKGRLSQAEIDRMVNEAEKYADEDEK      529
DmelHsp70Bbb     IE--VTFDLDANGILNVSAKEMSTGKAKNITIKNDKGRLSQAEIDRMVNEAEKYADEDEK      529
ScalHsp70        IE--VTFDLDANGILNVSAKEMSSGNAKNITIKNDKGRLSQAEIDRMVNEAEKYAEEDEK      529
DmelHsp68        ID--VTFDLDANGILNVTAKEQGTGNAKNITIKNDKGRLSQADIDRMLSEAEKYAEEDER      529
GmmHsp70A        ID--VTFDLDANGILNVTAKEMSTGNAKNITIKNDKGRLSQADIDRMVHEAEKYADEDEK      529
ScalHsp68        VD--VTFDLDANGILNVTAKEMSTGNAKNIVIKNDKGRLSQADIDRMVNEAERYAEEDEK      529
                 :    : *  : . :: * .    . .  :  .*       *:  :::.      .

EcHscC           --LKIYPRDMLINRTF--KAKLEE---LWARALGDEREEIGRVITDFDAALQSNDMARVD      541
HsHSPA9          KKERVEAVNMAEGIIHDTETKMEE---FKDQLPADECNKLKEEISKMRELLARKDSETGE      631
GmmHsc70-5       KRELVELVNQAEGIMHDTEAKMEE---FKNQLPAEECDKLKKSIADLRALLADKDKSEPE      635
DmelHsc70-5      KRELIEIVNQGESIVHDTETKMEE---FKSQLPAEECEKLKKEIADLRTLLANKETADLE      631
ScalHsc70-5      KRELVELVNQGESIVHDTETKMEE---FKSQLPAEECEKLKKEITELRELLANKETAELE      632
HsHSPA5          LKERIDTRNELESYAYSLKNQIGDKEKLGGKLSSEDKETMEKAVEEKIEWLESHQDADIE      614
GmmHsc70-3       LKEKVESRNELESYAYSLKNQIGDKDKLGAKLSEDDKTKMEAAIEDTIKWLDQNSDADPE      614
DmelHsc70-3      LKERVESRNELESYAYSLKNQIGDKDKLGAKLSDDEKNKLESAIDESIKWLEQNPDADPE      614
ScalHsc70-3      LKEKVETRNELESYAYSLKNQIGDKEKLGAKLSEDEKAKMEAAIDESIKWLETNADADPE      614
DmelHsc70-4      QKETIAAKNGLESYCFNMKATLDE-DNLKTKISDSDRTTILDKCNETIKWLDANQLADKE      590
GmmHsc70-4       QKETIAAKNALESYCFNMKATLDE-ENLKTKITESDRSTILDKCNETIKWLDANQLADKE      590
ScalHsc70-4      QKETIAAKNSLESYCFNMKATLDE-ENLKTKISESDRTTIMEKCNETIKWLDANQLADKE      590
DmelHsc70-1      QRDRINAKNQLESYCFQLRSTLDD-EHLSSRFSPADRETIQQRSSETIAWLDANQLAERQ      590
GmmHsc70-1       QRERINAKNTLESYCFQMKSTLDD-ENIKSKISDGDRQTILQKCNETIAWLDGNQQAEKE      590
ScalHsc70-1      QRERVNAKNALEGYCFQMKATIDD-ENIRSKVSESDRQMITQKCNETISWLDGNQMAEKD      504
HsHSPA6          QRDRVAAKNSLEAHVFHVKGSLQE-ESLRDKIPEEDRRKMQDKCREVLAWLEHNQLAEKE      592
HsHSPA1A         QRERVSAKNALESYAFNMKSAVED-EGLKGKISEADKKKVLDKCQEVISWLDANTLAEKD      590
HsHSPA1B         QRERVSAKNALESYAFNMKSAVED-EGLKGKISEADKKKVLDKCQEVISWLDANTLAEKD      590
HsHSPA1L         QREKIAAKNALESYAFNMKSVVSD-EGLKGKISESDKNKILDKCNELLSWLEVNQLAEKD      592
HsHSPA2          NRDRVAAKNALESYTYNIKQTVED-EKLRGKISEQDKNKILDKCQEVINWLDRNQMAEKD      593
HsHSPA8          QRDKVSSKNSLESYAFNMKATVED-EKLQGKINDEDKQKILDKCNEIINWLDKNQTAEKE      590
GmmHsp68         HRHRIEARNQLENCVYNVKQALEE-PG--EKVSPSDRSHLMGKCKVTLKWSDNNMTAKKE      588
DmelHsc70-2      QRERVQSKNNLEAYIYACRQAVDD-AP-SGVLSETERSKVRDKCSSEASWLDKNSLAEKE      590
ScalHsc70-2      QRQRVQARNNLESYIFSCKQAVED-AP-SDKLSDSDKATVRDKCSSEMSWLDNNSLAEKE      590
DmelHsp70Aa      HRQRITSRNALESYVFNVKQAVEQ-AP-AGKLDEADKNSVLDKCNDTIRWLDSNTTAEKE      587
```

```
DmelHsp70Ab       HRQRITSRNALESYVFNVKQAVEQ-AP-AGKLDEADKNSVLDKCNDTIRWLDSNTTAEKE        587
DmelHsp70Ba       HRQRITSRNALESYVFNVKQSVEQ-AP-AGKLDEADKNSVLDKCNETIRWLDSNTTAEKE        587
DmelHsp70Bb       HRQRITSRNALESYVFNVKQSVEQ-AP-AGKLDEADKNSVLDKCNETIRWLDSNTTAEKE        587
DmelHsp70Bc       HRQRITSRNALESYVFNVKQSVEQ-AP-AGKLDEADKNSVLDKCNETIRWLDSNTTAEKE        587
DmelHsp70Bbb      HRQRITSRNALESYVFNVKQSVEQ-AP-AGKLDEADKNSVLDKCNETIRWLDSNTTAEKE        587
ScalHsp70         QKNKIAARNNLESYVFGVKQALDQ-AG--DKISSQEKSEALRACEDTIKWLDANSLAEKE        586
DmelHsp68         HRQRIAARNQLETYLFGVKEAAEN-GG--DRISAADKSSIVERCSEAMKWLDSNTTAEKE        586
GmmHsp70A         HRQRIAARNQLETYVFGVKQALEE-AG--DKVNSSDKNRLMEKCTETIKWLDSNTTAEKD        586
ScalHsp68         HRQRIAARNQLESYVFSVKQAVED-AG--DKIPHSDKDRVLEKCKETIQWLDGNTTAEKE        586
                      :   :      .  .    :           :                 :     :

EcHscC            EVRRRASDYLAIEIP---------------------------------------------        556
HsHSPA9           NIRQAASSLQQASLKLFEMAYKKMASEREGSGSSGTGEQ---KED---------------        673
GmmHsc70-5        EVRKATNQLQQSSLKLFEMAYKKMSAERESAAGNKDSTT---ESTGEGGSGG--TSSSEQ        690
DmelHsc70-5       EVRKATSSLQQSSLKLFELAYKKMSAERESNAGAGSSDS---SSSSDT------------        676
ScalHsc70-5       DVRKATSNLQQSSLKLFEMAYKKMASERESQNAGSSSSE---EQP--S------------        675
HsHSPA5           DFKAKKKELEEIVQPIISKLYGSAGPPPTG---EEDTAEKDEL-----------------        654
GmmHsc70-3        EFKKQKKELETIVQPIIAKLYQGAGGVPPTDGGEESDDLKDEL-----------------        657
DmelHsc70-3       EYKKQKKDLEAIVQPVIAKLYQGAGGAPPPEGGDDA-DLKDEL-----------------        656
ScalHsc70-3       EYKKQKKDLEAIVQPIIAKLYQGTGGVPPTDSEDSD-DLKDEL-----------------        656
DmelHsc70-4       EYEHRQKELEGVCNPIITKLYQGAGFPPGGMPGG-PGGMPGAAGAAGAAGAGGAGPTIEE        649
GmmHsc70-4        EYEHRQKELEGVCNPIITKLYQAGGGAPTGMPTGMPGAFPGAAAAGAGGAASGAGPTIEE        650
ScalHsc70-4       EYEHRQKELEGVCNPIITKLYQGAGGAPGGMPGGMPGFPGGAG--AAPGAGSGAGPTIEE        648
DmelHsc70-1       EFEHKQQELERICSPIITRLYQGAGMAPPPTAG-----------GSNPGATGGSGPTIEE        639
GmmHsc70-1        EFEYKQKELEKICHPIITRLYQGGAPPSNPHG-------------AGGSASAGAGPTIEE        637
ScalHsc70-1       EYEHKQRELEKICNPIITRLYQGGAAPPPNAGG-----------MSGGPSGGSTGPTIEE        553
HsHSPA6           EYEHQKRELEQICRPIFSRLYGGP-----GVPGGSSCGT------QARQGDPSTGPIIEE        641
HsHSPA1A          EFEHKRKELEQVCNPIISGLYQGAGGPGP---GGFGAQ--------GPKGGSGSGPTIEE        639
HsHSPA1B          EFEHKRKELEQVCNPIISGLYQGAGGPGP---GGFGAQ--------GPKGGSGSGPTIEE        639
HsHSPA1L          EFDHKRKELEQMCNPIITKLYQGGCTGPA---CG--TG--------YVPGRPATGPTIEE        639
HsHSPA2           EYEHKQKELERVCNPIISKLYQGGPG-------GGSGGG---------GSGASGGPTIEE        637
HsHSPA8           EFEHQQKELEKVCNPIITKLYQSAGGMPGGMPGGFPGGG------APPSGGASSGPTIEE        644
GmmHsp68          EYECHLEELSEICEPITTGICQQSC------------SI----QRSHC-FDGGRSPAIEE        631
DmelHsc70-2       EFESHLKECQRICTPIMSKIHGGKK-------------------GKSSMGKGSHPTVEE        630
ScalHsc70-2       EFEDHLKDCQRVCSPIMSKMHGAGGAADG--------GQ----KQGQPNSGGRGGPTVEE        638
DmelHsp70Aa       EFDHKLEELTRHCSPIMTKMHQQG-AGAGAGGPGANCGQ----QAGGFG--GYSGPTVEE        640
DmelHsp70Ab       EFDHKLEELTRHCSPIMTKMHQQG-AGAGAGGPGANCGQ----QAGGFG--GYSGPTVEE        640
DmelHsp70Ba       EFDHKMEELTRHCSPIMTKMHQQG-AGA-AGGPGANCGQ----QAGGFG--GYSGPTVEE        639
DmelHsp70Bb       EFDHKMEELTRHCSPIMTKMHQQG-AGA-AGGPGANCGQ----QAGGFG--GYSGPTVEE        639
DmelHsp70Bc       EFDHKMEELTRHCSPIMTKMHQQG-AGA-AGGPGANCGQ----QAGGFG--GYSGPTVEE        639
DmelHsp70Bbb      EFDHKMEELTRHCSPIMTKMHQQG-AGA-AGGPGANCGQ----QAGGFG--GYSGPTVEE        639
ScalHsp70         EYEDKMKSMTQLCTPLMTKLHNGGG-------AGASCGQ----QAGGFGAG-RSGPTVEE        634
DmelHsp68         EYEYKLKELEQFCSPIMTKMHKGG----GDGQQAPNFGQ----Q-----AGGYKGPTVEE        633
GmmHsp70A         EFEYKLEELTKICQPVMTKMHQQQGAGSGDGSRSANCGQ----EAGGFGR----GPTVEE        638
ScalHsp68         EFEYKMQELTKICQPVMTRMHQQANGG----GSGNNCGQ----QSGGFGSGGYKGPTVEE        638
                  :      .

EcHscC            -----------         556
HsHSPA9           -----QKEEKQ         679
GmmHsc70-5        AKSQEKKEEKN         701
DmelHsc70-5       -SGEAKKEEKN         686
ScalHsc70-5       -SEEKKTEGKN         685
HsHSPA5           -----------         654
GmmHsc70-3        -----------         657
DmelHsc70-3       -----------         656
ScalHsc70-3       -----------         656
DmelHsc70-4       VD---------         651
GmmHsc70-4        VD---------         652
ScalHsc70-4       VD---------         650
DmelHsc70-1       VD---------         641
GmmHsc70-1        VD---------         639
ScalHsc70-1       VD---------         555
HsHSPA6           VD---------         643
HsHSPA1A          VD---------         641
HsHSPA1B          VD---------         641
HsHSPA1L          VD---------         641
HsHSPA2           VD---------         639
HsHSPA8           VD---------         646
GmmHsp68          ID---------         633
DmelHsc70-2       VDG--------         633
ScalHsc70-2       VD---------         640
DmelHsp70Aa       VD---------         642
DmelHsp70Ab       VD---------         642
DmelHsp70Ba       VD---------         641
DmelHsp70Bb       VD---------         641
DmelHsp70Bc       VD---------         641
DmelHsp70Bbb      VD---------         641
```

ScalHsp70	VD---------	636
DmelHsp68	VD---------	635
GmmHsp70A	VD---------	640
ScalHsp68	VD---------	640
