# Supplementary material for: Hsp70/J-protein machinery from Glossina morsitans morsitans, vector of African trypanosomiasis
Source: PLoS One. 2017 Sep 13;12(9):e0183858. doi: 10.1371/journal.pone.0183858 (PMC5597180; doi:10.1371/journal.pone.0183858)
Supplement: S2 Fig — Multiple sequence alignment of the full-length amino acid sequences of the Hsp110/HSPH gene families in humans, tsetse flies, fruit flies, and yeast. The multiple sequence alignment was performed using the in-built ClustalW program [43] with default parameters in the MEGA7 software [44]. Degree of amino acid conservation is symbolized by the following: (*) all fully conserved residues; (:) one of the residues is fully conserved and (.) residues are weakly conserved. Accession numbers of the sequences used: E. coli: HscC (NP_415183.1). S. calcitrans: Hsp110 (SCAU005995); Grp170 (SCAU010922). D. melanogaster: Hsp110 (NP_648687.1); Grp170 (NP_569995.1). H. sapiens: HSPH1 (NP_006635.2); HSPH2 (NP_002145.3); HSPH3 (NP_055093.2); HSPH4 (NP_006380.1). Accession numbers for the G. m. morsitans Hsp110 sequences can be found in Table 1. (PDF) [file pone.0183858.s002.pdf]

```
EcHscC          ----------------------------------MDNAELAIGIDLGTTNSLIAVWKDGA   26
HsHSPH3         --------------------------------------MSVVGIDLGFLNCYIAVARSGG   22
HsHSPH1         --------------------------------------MSVVGLDVGSQSCYIAVARAGG   22
HsHSPH2         --------------------------------------MSVVGIDLGFQSCYVAVARAGG   22
GmmHs110-2      --------------------------------------MSVIGIDFGNDSCYVATVKNGG   22
DmelHsp110      --------------------------------------MSVIGIDFGNESCYVAAARSGG   22
GmmHsp110-1     --------------------------------------MSVIGIDFGNESCFVAAAKAGG   22
ScalHsp110      --------------------------------------MSVIGIDFGSESCYIAAAKAGG   22
HsHSPH4         MADKVRRQRPRRRVCWALVAVLL------ADLLALSDTLAVMSVDLGSESMKVAIVKPGV   54
DmelGrp170      -------------------MKLVLLLSALLAGIALSQGAAVMSVDLGSEWMKVGVVSPGV   41
GmmGrp170       ----MMINKKHRASSWWSLSVAVSLFIVLSTYVCEVQAAAVMSVDLGSEWMKVGVVSPGV   56
ScalGrp170      ------MLHKKTTSCWWSMAAVACLLFVTAFHIRGVQSAAVMSVDLGSEWMKVGIVSPGV   54
                                                        .:.:*.*     :.    *

EcHscC          -AQLIPNKFGEYLTPSIISMDENNHILVGKPAVSRRTSHPDKTAALFKRAMGSNT-----   80
HsHSPH3         -IETIANEYSDRCTPACISLGSRTRA-IGNAAKSQIVTNVRNTIHGFKKLHGRSFDDPIV   80
HsHSPH1         -IETIANEFSDRCTPSVISFGSKNRT-IGVAAKNQQITHANNTVSNFKRFHGRAFNDPFI   80
HsHSPH2         -IETIANEYSDRCTPACISFGPKNRS-IGAAAKSQVISNAKNTVQGFKRFHGRAFSDPFV   80
GmmHs110-2      -VETLDNDYSLRATPSFVAFAGRKRI-IGAAAKNQYGSNKENTVVGFKRLLGRKFDDPHV   80
DmelHsp110      -IETLANDYSLRATPSFVAFDGKKRI-IGVAAKNQQVTNMKNTVGGFKRLLGRKFNDPHV   80
GmmHsp110-1     -IETLANDYSLRATPSCVAFDGKKRI-IGVAAKNQQVTNMKNTVSGFKRLLGRKFNDPHV   80
ScalHsp110      -IETLANDYSLRATPSCVAFDGKKRI-IGVAAKNQQVTNMKNTVSGFKHLLGRRFNDPHV   80
HsHSPH4         PMEIVLNKESRRKTPVIVTLKENERF-FGDSAASMAIKNPKATLRYFQHLLGKQADNPHV   113
DmelGrp170      PMEIALNRESKRKTPAILAFRDGTRT-IGEDAQTIGIKDPNSAYGYLLDLLGKTIDNPIV   100
GmmGrp170       PMEIALNRESKRKTPATLAFRDGVRT-FGEDALTVGIRDPSSAYGYLIDLLGKTIDNPIV   115
ScalGrp170      PMEIALNRESKRKTPVTLSFRNGVRS-FGDDAVTDGIKDPASVYSYLLDLLGKQIDNPIV   113
                  :   *  .   **  :::    :  .*  * .    .   .   :    *

EcHscC          -----------------------NWRLGSDTFNAPELSSLVLRSLKEDAEEFLQRPIKDV   117
HsHSPH3         QTERIRLPYELQKMPNGSAGVKVRYLEEERPFAIEQVTGMLLAKLKETSENALKKPVADC   140
HsHSPH1         QKEKENLSYDLVPLKNGGVGIKVMYMGEEHLFSVEQITAMLLTKLKETAENSLKKPVTDC   140
HsHSPH2         EAEKSNLAYDIVQLPTGLTGIKVTYMEEERNFTTEQVTAMLLSKLKETAESVLKKPVVDC   140
GmmHs110-2      QHELTMIPTKIEERSDNGINICVHYLGQTHFFTPEQVTAMLLTKLKETSSQALEAQVSDC   140
DmelHsp110      QHELTSIPARVEARGDGSIGIKVNYLGEDQHFGPEQLTAMLFTKLKETSAAAMQTQVNDC   140
GmmHsp110-1     QHELNNIPTKVEQMSDGGIGFCVNYLDQQQLFTPEQLTAMLFTKLKETSTHALQAQVNDC   140
ScalHsp110      QRELNSIPTKVKGLPDGSIGYQVNYLDQEQCFTPEQLTAMLFTKLKETSTNALEAQVNDC   140
HsHSPH4         ALYQARFPEHEL-TFDPQRQTVHFQISSQLQFSPEEVLGMVLNYSRSLAEDFAEQPIKDA   172
DmelGrp170      DLYRKRFPYYNI-VGDPERNTVVFRKSDTDEFSVEELVAQLLVKAKQFAQESVQQPITEC   159
GmmGrp170       DLYRKRFPYYDI-IGDPERNTVIFKKNDKEQFLVEELVAQILMKAKEFAQESTHQPITEA   174
ScalGrp170      ELYRKRFPYYDI-IPDAERNTVVFKKSDTEQFSVEELIAQILVKAKEFAQDATNQPITEC   172
                                               *   :: . ::   :. :    .  : :

EcHscC          VISVPAYFSDEQRKHTRLAAELAGLNAVRLINEPTAAAMAYGLHTQQNT-------RSLV   170
HsHSPH3         VISIPSFFTDAERRSVMAAAQVAGLNCLRLMNETTAVALAYGIYKQDLPPLDEKPRNVVF   200
HsHSPH1         VISVPSFFTDAERRSVLDAAQIVGLNCLRLMNDMTAVALNYGIYKQDLPSLDEKPRIVVF   200
HsHSPH2         VVSVPCFYTDAERRSVMDATQIAGLNCLRLMNETTAVALAYGIYKQDLPALEEKPRNVVF   200
GmmHs110-2      VIACPIYFTNAERQALLDAAKIAGLNVLRLLNETTAIALSYGFYKQTLP--SDNPRNVIF   198
DmelHsp110      VIACPVFFTNAERKALLDAAQIAGLNVLRLMNETTATALAYGFYKNDLF--EDKPRNVIF   198
GmmHsp110-1     VIACPVYFTNAERKALLDAAQIAGLNVLRLMNETTATALSYGFYKQDLP--DDKPRNVIF   198
ScalHsp110      VITCPVYFTNAERKALLEAAHIAGLNVLRLMNETTATALSYGFYKQDLP--EDKPRNVIF   198
HsHSPH4         VITVPVFFNQAERRAVLQAARMAGLKVLQLINDNTATALSYGVFRRKDI--NTTAQNIMF   230
DmelGrp170      VLTVPGYFGQAEREALLSAAQLANLKVLQLINDYAAVALNYGVFHRGEI--NETAQYFLF   217
GmmGrp170       VLTVPGYFGQAEREALLTAAQLANIKVLQLINDYTAVALNYGVFHPSGI--NETAQYYIF   232
ScalGrp170      VITVPGYFGQAEREALLTAAQLANLKVLQLINDYTAVALNYGVFHPSGI--NETTQYYIF   230
                *:: * :: : :*.    *:.:..:: ::*:*: :* *: **..              :.

EcHscC          FDLGGGTFDVTVLEYA----------TPVIEVNASAGDNFLGGEDFTHMLVDEVLKRAEV   220
HsHSPH3         IDMGHSAYQVLVCAFN----------KGKLKVLATTFDPYLGGRNFDEALVDYFCDEFKT   250
HsHSPH1         VDMGHSAFQVSACAFN----------KGKLKVLGTAFDPFLGGKNFDEKLVEHFCAEFKT   250
HsHSPH2         VDMGHSAYQVSVCAFN----------RGKLKVLATAFDTTLGGRKFDEVLVNHFCEEFGK   250
GmmHs110-2      VDCGDSSLQVCACAFT----------KGKLKMLASSWDQ-VGGRDFNVAMADYFAKEFKQ   247
DmelHsp110      VDFGHSSLQASACAFT----------KGKLKMLASTWDQ-IGGRDIDLALGDYFAKEFQE   247
GmmHsp110-1     VDCGNSSLQVSACAFT----------KGKLKMLASSWDQ-IGGRDFDAAMADYFAKEFMD   247
ScalHsp110      VDCGHASLQVSACAFT----------KGKLKMLASSWDQ-IGGRDFDTTLAEYFAKEFLD   247
HsHSPH4         YDMGSGSTVCTIVTYQMVKTKEA-GMQPQLQIRGVGFDRTLGGLEMELRLRERLAGLFNE   289
DmelGrp170      YDMGAYKTSAAVVSYQLVKDKQTREINPVVQVLGVGYDRTLGGLEIQLRLRDYLAQEFNA   277
GmmGrp170       YNMGASSTSAAVISYQLVKDKHTKESHPVVEVLGVGYDRTLGGLEIQLRLRDYLADEFNA   292
ScalGrp170      YDMGASHTSSALVSYQLVKDKHTKETNPVVQVLGVGFDRTLGGLEVQLRLRDYLAVEFNA   290
                 : *          :              ::: .   *  :** ..   : : .

EcHscC          AK-------TTLNDSELAALYACVEAAK-----CSNQSPMHIRWQYQDEMRKCEFYENEL   268
HsHSPH3         KY----KINVKENSRALLRLYQECEKLKKLMSANASDLPLNIECFMNDLDVSSKMNRAQF   306
HsHSPH1         KY----KLDAKSKIRALLRLYQECEKLKKLMSSNSTDLPLNIECFMNDKDVSGKMNRSQF   306
HsHSPH2         KY----KLDIKSKIRALLRLSQECEKLKKLMSANASDLPLSIECFMNDVDVSGTMNRGKF   306
GmmHs110-2      RY----KIEIENNSRQWLRLLGEVDKMKKQMSTNNTNLPLDIECFIDDMDVSSSMHRYQM   303
DmelHsp110      RY----KINAKTNARANLRLLTEIEKLKKQMSANSTKLPLNIECFLDDIDVSSSMQRSQM   303
GmmHsp110-1     RY----KINAKSNPRSWLRLLTEIEKLKKQMSANSTKLPLAIECFMDDIDVSSSMQRAQM   303
```

```
ScalHsp110        RY----KINAKTNARSWLRLLTEIEKLKKQMSANSTKLPLGIECFMDEIDVSSSMQRSTM         303
HsHSPH4           QRKGQRAKDVRENPRAMAKLLREANRLKTVLSANADHM-AQIEGLMDDVDFKAKVTRVEF         348
DmelGrp170        LKKT--KTDVTTSPRALAKLFKEAGRLKNVLSANTEFF-AQIENLIEDIDFKLPVTREKL         334
GmmGrp170         MRKT--STDVKTSPRALAKLFKEAGRVKNVLSANMDHF-AQIENVLEDQDFKLQISREKL         349
ScalGrp170        MKKT--KTDVTKSPRALAKLFKEAGRLKNVLSANNDHY-AQIENLIEDQDFKLQVTREKL         347
                              .      *       *             *.   ::   .  . .  :

EcHscC            EDLWLPLLNRLRVPIEQALRDARLKPSQIDSLVLVGGASQMPLVQRIAVRLFGKLP-YQS         327
HsHSPH3           EQLCASLLARVEPPLKAVMEQANLQREDISSIEIVGGATRIPAVKEQITKFFL-KDISTT         365
HsHSPH1           EELCAELLQKIEVPLYSLLEQTHLKVEDVSAVEIVGGATRIPAVKERIAKFFG-KDISTT         365
HsHSPH2           LEMCNDLLARVEPPLRSVLEQTKLKKEDIYAVEIVGGATRIPAVKEKISKFFG-KELSTT         365
GmmHs110-2        EILCDSLLRRAEDTFNRLLIESKLSLKEIHSVEIVGGTTRIPAIKELIEKVFG-LTVCTT         362
DmelHsp110        EELCAPVLQRVEQTFKRLLAESKLQLDDIHSVEIVGGSSRIPSVKQLIEQVFN-KPASTT         362
GmmHsp110-1       EELCGPLLKRVEATFKKLLVESKLPLEEIHSVEIVGGSTRIPSIKLIIEQVFG-KPARTT         362
ScalHsp110        EELCAPLFQRVEMTFKRLLAESKLTLDEIHSVEVVGGSTRIPAIKQLIENVFG-KPASTT         362
HsHSPH4           EELCADLFERVPGPVQQALQSAEMSLDEIEQVILVGGATRVPRVQEVLLKAVGKEELGKN         408
DmelGrp170        EQLCEDLWPRATKPLEEALASSHLSLDVINQVILFGGGTRVPRVQETI-KAVIKQELGKN         393
GmmGrp170         EEICADLWPRIIKPLERALAHSGLSLNIISQVIIFGGGTRVPKVQDTI-KQFIKEDLGKS         408
ScalGrp170        EEICSDLWPRVIKPLEQALSSSGLTLDVVSQVILFGGGTRVPKVQETL-KQYIKQELGKS         406
                    :   :  :    .   :  : :  . :  : :.** :::* ::    .         .

EcHscC            YDPSTIVALGAAIQAACRLRNEDIEEVILTDICPYSLGVEVNRQG-VSGI--F------S         378
HsHSPH3           LNADEAVARGCALQCAILSPAFKVREFSITDLVPYSITLRWKTSF-EDGSGEC-------         417
HsHSPH1           LNADEAVARGCALQCAILSPAFKVREFSVTDAVPFPISLIWNHDS-EDTEGVH-------         417
HsHSPH2           LNADEAVTRGCALQCAILSPAFKVREFSITDVVPYPISLRWNSPA-EEGSSDC-------         417
GmmHs110-2        LNQDEAVSRGAALQCALMSPALRVRKFETTDIQNYAVEVTWTGEGSCIG-SSV-------         414
DmelHsp110        LNQDEAVSRGAALQCAIMSPAVRVREFGVTDIQNYAVKVLWDSEGSAAP-GEI-------         414
GmmHsp110-1       LNQDEAVSRGAALQCAIMSPAVRVREFGVTDIQNYSVKVTWDGEDSMVG-GGI-------         414
ScalHsp110        LNQDESVSRGAALQCAIMSPAVRVREFGVTDIQNYAVKVICDGEGSATGSSGL-------         415
HsHSPH4           INADEAAAMGAVYQAAALSKAFKVKPFVVRDAVVYPILVEFTREV-EEEPGIHSLKHNKR         467
DmelGrp170        LNADESATMGAVYKAADLSAGFKVKKFVVKDATLFPLQVSFERDP-GDGA---AVKQVKR         449
GmmGrp170         LNADEAAAIGAVYKAADLATGFKVKKFVVKDAAVLPIHVAFERET-GNGA---EIKHVKR         464
ScalGrp170        LNADEAAAIGAVYKAADLATGFKVKKFVVKDAVLYPIQVTFERDP-GEGA---EVKQVKR         462
                   : .  .: *.. :.*       :. .   *     : :

EcHscC            PIIERNTTVPVSRVETYSTMHPEQDSITVNVYQGENHKVK----------------NNIL         422
HsHSPH3           EVFCKNHPAPFSKVITFHKK----EPFELEAFYTNLHEVPYPDA-RIGSFTIQNV-----         467
HsHSPH1           EVFSRNHAAPFSKVLTFLRR----GPFELEAFYSDPQGVPYPEA-KIGRFVVQNV-----         467
HsHSPH2           EVFSKNHAAPFSKVLTFYRK----EPFTLEAYYSSPQDLPYPDP-AIAQFSVQKV-----         467
GmmHs110-2        EIFPAFHEAPTSRLLTLSRK----EPFTLTVRYS--QSIPYPDP-IIGHWTIKEI-----         462
DmelHsp110        EIFPQYHASPFSRLLTINRK----GPFNVSIVYG--QQVPYPDQ-TIGVWKVKDV-----         462
GmmHsp110-1       EVFPTFHAAPFSRLLTLNRK----EPFTMTVQYA--QPVPYPDL-IIGRWTIKDI-----         462
ScalHsp110        EVFSVFHTAPFSRMLTLHRK----EPFTVTVLYA--QPIPYPDP-TIGRWTIKDV-----         463
HsHSPH4           VLFSRMGPYPQRKVITFNRY---SHDFNFHINYGDLGFLGPEDLRVFGSQNLTTVKLKGV         524
DmelGrp170        ALFALMNPYPQKKVITFNKH---TDDFEFYVNYADLDRYSKEEIAALGSLNVTKVQLKQV         506
GmmGrp170         VLFGLMNSYPQKKVITFNKH---TSDFQFHVCYGDLTHLSSEERLFVGATNISLVELENI         521
ScalGrp170        VLFGLMNAYPQKKVITFNKH---TDDFDFFVHYGDLNHLSAEEQAYLGGLKISRVELQKI         519
                   ::      *  :: *          : .

EcHscC            VESFDVPLKKTGTYQSIDIRFSYDINGLLEVDVLLEDGSVKSRVINHSPVTLSAQQIEES         482
HsHSPH3           ------FPQSDGDSSKVKVKVRVNIHGIFSVASASVIE---------------KQNLEGD         506
HsHSPH1           ------SAQKDGEKSRVKVKVRVNTHGIFTISTASMVE---------------KVPTEEN         506
HsHSPH2           ------TPQSDGSSSKVKVKVRVNVHGIFSVSSASLVE---------------VHKSEEN         506
GmmHs110-2        ------KPNNRGEYKDIKVKVKINQHGLVVITRATLSD---------------KNDDAER         501
DmelHsp110        ------KPTERGEGQDVKLKVRINNNGIVLISSATLVE---------------KKEAEEA         501
GmmHsp110-1       ------KPNERGEPSEVKVKVRINHHGIVLISSASLVD---------------KKEVEES         501
ScalHsp110        ------KPTDKGELSTIKVKVRINHHGIVLISSAHLVD---------------SKEVEEP         502
HsHSPH4           GDSFKKYP--DYESKGIKAHFNLDESGVLSLDRVESVF--------------ETL-VEDS         567
DmelGrp170        KELLEKSKKELVDNKGIKAYFYLDDSGIFRCTGVEYVY--------------EKQKPEDD         552
GmmGrp170         KDILEKMSNESVEAKGIKAYFALDDSGIFRCTGVEYVY--------------DKQKIE--         565
ScalGrp170        KELLEKTKDESVEAKGIKAFFTLDDSGIFRCTGVEYVY--------------EKQKVETE         565
                                . :.  .  :  *:.

EcHscC            RTRLSALKIYPRDMLINRTFKAKLEEL----WARALGDER--------EEIGRVI-----         525
HsHSPH3           HSDAPMETE--T------SF-K----NENKDNMDKMQVDQ----EEGHQKCHAEH-----         544
HsHSPH1           E--MSSEAD--M------EC-LNQRPPENPDTDKNVQQDN----SEAGTQPQVQTDAQQ-         550
HsHSPH2           E--EPMETD--Q------NA-KEE---------EKMQVDQ--------EEPHVEEQQQ--         536
GmmHs110-2        RSTSLQYTS------------TEQEKQ---ST----IEDF----NSR-KNPKPSH--A--         533
DmelHsp110        AAAAE-------------------QAASEEKP----GDQT----NNT-GEPADGQ--Q--         529
GmmHsp110-1       SSNSQLNEP--Q------TNSAEQLNSGQDQP----QNQI----NNA-GEPMDVS--Q--         540
ScalHsp110        VTPQPA-EP----------------ASAEQPAV----GEQQ----QNN-GEPMEVQ--Q--         533
HsHSPH4           AEEESTLTK--LGNTISSLFGGGTTPDAKENGTDTVQEEEESPAEGSKDEPGEQVELKEE         625
DmelGrp170        ADEDSTLSK--FGSTLSKLFTKEGEEKKDNSEQEEA--------ANAGEEPSKS-EDNEK         601
GmmGrp170         -EDEGTLAK--LGNTISKLFSKDSKEEDKKVEDVKE--------EVGEDEKIKEEKSEEK         614
ScalGrp170        EDDEGTLAK--LGSTISKLFSKDKEEEKKEENKE-S--------SSGSANEGEE------         608
                                                                   :
```

```
EcHscC          -----------TDFDAALQ-------SND---------------------M--ARVDEV          543
HsHSPH3         ------------TPEEEIDHTGAKTKSAV----------------SDKQDRLN------Q          570
HsHSPH1         ------TS--QSPPSPELTSEENKIPDAD----------------KANEKKVDQPPE--A          584
HsHSPH2         ----------QTPAENKAESE-EMETSQA----------------GSKDKKMDQPPQ--A          567
GmmHs110-2      ------NT--STD---------------------------------------KQTNKEM--D       546
DmelHsp110      ------EG--A-------------------------------------------------          532
GmmHsp110-1     ------EA--CKDYEEEYNTTASSPQGGQ--------------GFAQRVKGWFSSGT--S          576
ScalHsp110      ------EA--CNDNEDDDSTNTASSPGGQ--------------GWAQRVKGWFTPGT--E          569
HsHSPH4         AEAPVEDGSQPPPPEPKGDATPEGEKATEKENGDKSEAQKPSEKAEAGPEGVAPAPEGEK          685
DmelGrp170      --AKEEDAS-------KEQ---KSEESTKQDTE--------------------------A          623
GmmGrp170       --VNMENED-------KYNKTTAEKNLDEKENN--------------------------K          639
ScalGrp170      --ANKEEST-------KETEKNETMKAAEAEAS--------------------------K          633

EcHscC          RRRASDYLAIEIP-----------------------------------------------          556
HsHSPH3         TLKKGKVKSIDLPIQSSLCRQLGQDLL-NSYIENEGKMIMQ---DKLEKERNDAKNAVEE          626
HsHSPH1         KKPKIKVVNVELPIEANLVWQLGKDLL-NMYIETEGKMIMQ---DKLEKERNDAKNAVEE          640
HsHSPH2         KKAKVKTSTVDLPIENQLLWQIDREML-NLYIENEGKMIMQ---DKLEKERNDAKNAVEE          623
GmmHs110-2      NENKKSSKRMDLPMEFQTHGIC-QKEL-KNLRLFEERMSAN---DASETKRIDAKNALEE          601
DmelHsp110      DKKKKASKATELPLECTTHGFS-PVDL-SNYTQQESKMIGN---DQKETERIDAKNALEE          587
GmmHsp110-1     EKKKKLSKLIELPMDYQTHGYL-PNEL-NNLIQLEAKMVAN---DAKETKRVDAKNALEE          631
ScalHsp110      K-KKKNTKMIDLPMDCESHGHS-LNDL-NNLIQQEAKMVAN---DAKETERIDAKNALEE          623
HsHSPH4         KQKPARKRRMVEEIGVELVVLDLPDLPEDKLAQSVQKLQDLTLRDLEKQEREKAANSLEA          745
DmelGrp170      KNETIKLVTVKSPVTYESQTQFVVPLVGSAYDQSVAKLAAINKAEEQRVRLESAFNALEA          683
GmmGrp170       KNETVKIVTIKEPLQHKVTFQFTPPLTGKSYEQSLEKLNAINKAETMRSRLESSFNALES          699
ScalGrp170      KNETIKIVTIKEPVAHTLTLQFTAPLSGEAYEKAAEKLDAINKAENLRNRLESSFNALES          693

EcHscC          ------------------------------------------------------------          556
HsHSPH3         YVYDFRDRL-GTVYEKFITPEDLSKLSAVLEDTENWLYEDGEDQPKQVYVDKLQELKKYG          685
HsHSPH1         YVYEFRDKL-CGPYEKFICEQDHQNFLRLLTETEDWLYEEGEDQAKQAYVDKLEELMKIG          699
HsHSPH2         YVYEMRDKL-SGEYEKFVSEDDRNSFTLKLEDTENWLYEDGEDQPKQVYVDKLAELKNLG          682
GmmHs110-2      YVYEMRSKLLGGHLDQHIFPIERDNICTELNDVENWLYEDGVDTDYETYMDKLYLLKQRI          661
DmelHsp110      FVYDMRNKLQGGPFERYVVEAEREKIVSQLNDLENWLYEDGEDCERDIYTSRLQALHQKT          647
GmmHsp110-1     F--------GDGPLERYVVSSARDSICNQLNDLENWLYEDGEDCDHDTYVDKLKSLQNQT          683
ScalHsp110      FVYDMRNKLQGGPLERYVVEAERDAICAQLNDLENWLYEDGEDCDRDTYVNRLKALHNKT          683
HsHSPH4         FIFETQDKLYQPEYQEVSTEEQREEISGKLSAASTWLEDEGVGATTVMLKEKLAELRKLC          805
DmelGrp170      HIIEVQQKLDEESYAKCATAEEKEKLLAECSTLGEWLYEDLEDPKAEIYEEKLAQLKKLS          743
GmmGrp170       HIIDIQQKLEEEEYASCLTSEEKQKILSECSTISDWLYDDSDKTTHDMYEAKLNDLKMLT          759
ScalGrp170      HIIDMQQKLEEEEYASCATEEEKSKILAECSAMSDWLYEDIENPTPEMYENKLKSLKKLT          753

EcHscC          ------------------------------------------------------------          556
HsHSPH3         QPIQMKYMEHEERPKALNDLGKKIQLVMKVIEAYRN----KDERYDHLDPTEMEKVEKCI          741
HsHSPH1         TPVKVRFQEAEERPKMFEELGQRLQHYAKIAADFRN----KDEKYNHIDESEMKKVEKSV          755
HsHSPH2         QPIKIRFQESEERPKLFEELGKQIQQYMKIISSFKN----KEDQYDHLDAADMTKVEKST          738
GmmHs110-2      NPINVRANDYERCPLAFDELERSISFARTVMADFKK----GLDKYAHFTEAESLNISEKI          717
DmelHsp110      DPIKLRASDYEQGPAAFDELKNSIAIARLAVAEFRK----GVPKYDHLTETEFINISETA          703
GmmHsp110-1     NPIKERANDYEFCPAAFDELKQAIAFARTAHSEFKK----GIPKYDHLTETEFMNISETT          739
ScalHsp110      NPIKDRANDYELCPSVFSELKHAINNAHVAVNEFKK----GSPKYDHLTETEFINIAEHA          739
HsHSPH4         QGLFFRVEERKKWPERLSALDNLLNHSSMFLKGARLIPE----MDQIFTEVEMTTLEKVI          861
DmelGrp170      NVFLARHWEHEERPEAIKALKGMIDGAEKFLVTGRNLTKDTNPEKDVFTQVEIDTLDKVI          803
GmmGrp170       NVFMARHWEHEERPEAVKALDNMIDGAQQFLENAKNLTKDANPERDVFTQIEIDTLSRII          819
ScalGrp170      DVFMARHWEHDERPEAMKALTRMIEGAEGFLNSAKNLTKETNPEKDVFTQVEIDTLSKVI          813

EcHscC          ------------------------------------------------------------          556
HsHSPH3         SDAMSWLNSKMNAQNKLSLTQDPVVKVSEIVAKSKELDNFCNPIIY-----KPKPKAEVP          796
HsHSPH1         NEVMEWMNNVMNAQAKKSLDQDPVVRAQEIKTKIKELNNTCEPVVT-----QPKPKIESP          810
HsHSPH2         NEAMEWMNNKLNLQNKQSLTMDPVVKSKEIEAKIKELTSTCSPIIS-----KPKPKVEPP          793
GmmHs110-2      DRTQKWFDRNKARFIEAARTSDCPVNVTQIRYEADMLTSYVNSAIN-----RSKLNRNIP          772
DmelHsp110      DKAQSWLDANLPKFTQSPRTADSPVQISAVRQEVQTLNSCVSSVIN-----RAKPKPTPA          758
GmmHsp110-1     EKVQKWLDINLAKFSQAARTTDSPVKINEIRHEIQTLTTCVNSVIN-----RPKPKPA--          792
ScalHsp110      DKSQKWLDQHMAKFGASPRTVDSPVKVADIRHEVQTLNACVNSVIN-----RPKPKPA--          792
HsHSPH4         NETWAWKNATLAEQAKLPATEKPVLLSKDIEAKMMALDREVQYLLNKAKFTKPRPRPKDK          921
DmelGrp170      TETNAWLKTETAAQKKLAKNADIRLTVKDITDKMSLLDREVKYLVNKIKIWKPKVKPAAE          863
GmmGrp170       DETVTWKNGETEAQNKLQRHEKVRLTVKDITDHMASLDREVKYMVNKIKIWKPKPKPTAK          879
ScalGrp170      SDTNEWKQTESQAQKKLKRHENVRLTIKDITAKMSTLDREVKYLVTKINLWKPKVKPTPK          873

EcHscC          ------------------------------------------------------------          556
HsHSPH3         EDKPKANSEHNGPMDGQSGTETKSDSTK---------DS----SQHTKSS--GEMEV---          838
HsHSPH1         KLERTP-NGPNIDKKEEDLEDKNNFGAEPPHQN-GECYP----N--EKNS--VNMDL---          857
HsHSPH2         KEEQKN-AEQNGPVDG----QGDNPGPQAAEQGTDTAVP----SDSDKKL--PEMDI---          839
GmmHs110-2      KISSATIMSLL-------------------------------------------------          783
DmelHsp110      KTATPP-KDEANAEQNGGEPAAN-SGDKMDV---DN-NG----QSAAGND--PSMEV---          803
GmmHsp110-1     SKPAPA-KDMTGTDQQQQQHNGE---DKKTE----S-AH----TNSAPED--STMDV---          834
```

```
ScalHsp110       AKPAPP-KDNAAGADQHNGENAD-KKSEGDK----T-AH----PNSVPDD--STMDV---         836
HsHSPH4          NGTRAE--PPLNASASDQGE-KVIPPAGQTEDAEPISEPEKVETGSEPGDTEPLELGG--         976
DmelGrp170       KEKKKE--EEV-----------VASGSGDDTKSEDAEQQQEQATKEEQQEQEPVDEI---         907
GmmGrp170        SDKKTS--KKSGEVETEEMG----SGFGNETQSDDHLSS----------QQSPDEDI---         920
ScalGrp170       KDKKKD--KKEGDEEQKSGDEEASSGDDEAEKPNEQSEE-----QKEQQVETPEAEEKSS         926

EcHscC           ----------------------------   556
HsHSPH3          ----------D-----------------   839
HsHSPH1          ----------D-----------------   858
HsHSPH2          ----------D-----------------   840
GmmHs110-2       ----------------------------   783
DmelHsp110       ----------E-----------------   804
GmmHsp110-1      ----------D-----------------   835
ScalHsp110       ----------E-----------------   837
HsHSPH4          -----PGAEPEQKEQSTGQKRPLKNDEL   999
DmelGrp170       --------TPTPAEEETK----TPHSEL   923
GmmGrp170        --------KVNTTEQSTT----TPRTEL   936
ScalGrp170       QETAQDTSENTTPTDDDH----TPRSDL   950
```
