# Supplementary material for: Hsp70/J-protein machinery from Glossina morsitans morsitans, vector of African trypanosomiasis
Source: PLoS One. 2017 Sep 13;12(9):e0183858. doi: 10.1371/journal.pone.0183858 (PMC5597180; doi:10.1371/journal.pone.0183858)
Supplement: S3 Fig — The multiple sequence alignment was performed using the in-built ClustalW program [43] with default parameters on the MEGA7 software [44]. Degree of amino acid conservation is symbolized by the following: (*) all fully conserved residues; (:) one of the residues is fully conserved and (.) residues are weakly conserved. Accession numbers of the sequences used: E. coli: DnaJ (NP_308042.1). S. calcitrans: ScalJA1 (SCAU009538); ScalJA2 (SCAU013613); ScalJA3 (SCAU003912); ScalJB4 (SCAU013247); ScalJB5 (SCAU015003); ScalB11 (SCAU015416). D. melanogaster: DnaJ-1 (NP_523936.2); CG5001 (NP_608586.2); AT19485 (NP_572633.1); Droj2 (NP_650283.1); Tid56 (NP_524932.2); DnaJ-H (NP_609605.1); CG4164 (NP_608525.1). H. sapiens: DnaJA1 (NP_001530.1); DnaJA2 (NP_005871.1); DnaJA3 (NP_005138.3); DnaJA4 (NP_061072.3); DnaJB1 (NP_006136.1); DnaJB4 (NP_008965.2); DnaJB5 (NP_001128476.2); DnaJB11 (NP_057390.1); DnaJB13 (NP_705842.2). Accession numbers for the G. m. morsitans J-protein sequences can be found in Table 2. (PDF) [file pone.0183858.s003.pdf]

```
DmelAT19485     ------------------------------------------------------------     0
HsDnaJB13       ------------------------------------------------------------     0
HsDnaJB5        MGGAEAEPWGRAPGPAIGGRRAGDSCPG-WRRRSRSR---------GRGQR-------LS     43
HsDnaJB1        ------------------------------------------------------------     0
HsDnaJB4        ------------------------------------------------------------     0
GmmJB5          ------------------------------------------------------------     0
DmelCG5001      ------------------------------------------------------------     0
ScalJB5         ------------------------------------------------------------     0
DmelDnaJ-1      ------------------------------------------------------------     0
GmmJB1          ------------------------------------------------------------     0
GmmJB4          ------------------------------------------------------------     0
ScalJB4         ------------------------------------------------------------     0
HsDnaJB11       ------------------------------------------------------------     0
GmmJB11         ------------------------------------------------------------     0
DmelCG4164      ------------------------------------------------------------     0
ScalJB11        ------------------------------------------------------------     0
DmelDnaJ-H      ------------------------------------------------------------     0
GmmJA2          ------------------------------------------------------------     0
ScalJA2         ------------------------------------------------------------     0
HsDnaJA2        ------------------------------------------------------------     0
DmelDroj2       ------------------------------------------------------------     0
GmmJA1          ------------------------------------------------------------     0
ScalJA1         ------------------------------------------------------------     0
HsDnaJA1        ------------------------------------------------------------     0
HsDnaJA4        ------------------------------------------------------------     0
GmmJB15         --------------------------------------------------------MKSV     4
GmmJD1          ------------------------------------------------------------     0
EcDnaJ          ------------------------------------------------------------     0
HsDnaJA3        ---------------------MAARCSTRWLLVV--VGTPRLPAISGRGARPPREGVVGA     37
GmmJA3          ----------------------MISCNSLNLRILRQASIKIVRYQILE-----SNGSVYK     33
DmelTid56       ---------------------MMISCKKLFVFR--Q-----LPAVRR-------------     19
ScalJA3         ----------------------MITCNRLNLHLLRKVSIKNYPAQSLL-------SITYN     31

DmelAT19485     ------------------------------------------------------------     0
HsDnaJB13       ------------------------------------------------------------     0
HsDnaJB5        HGPRRRPQLLTAAPPLQARGAFRSFPHSWGEDFLASLMFKIQLEPLKLRAWT--LNGF--     99
HsDnaJB1        ------------------------------------------------------------     0
HsDnaJB4        ------------------------------------------------------------     0
GmmJB5          ------------------------------------------------------------     0
DmelCG5001      ------------------------------------------------------------     0
ScalJB5         ------------------------------------------------------------     0
DmelDnaJ-1      ------------------------------------------------------------     0
GmmJB1          ------------------------------------MLWEIQLARRLSR--T--QQKF--     18
GmmJB4          ------------------------------------------------------------     0
ScalJB4         ------------------------------------------------------------     0
HsDnaJB11       ------------------------------------------MAPQNL------------     6
GmmJB11         ------------------------------------------MKFLIL------------     6
DmelCG4164      ------------------------------------------MQLIKC------------     6
ScalJB11        ------------------------------------------MKAVAL------------     6
DmelDnaJ-H      ------------------------------------------------------------     0
GmmJA2          ------------------------------------------------------------     0
ScalJA2         ------------------------------------------------------------     0
HsDnaJA2        ------------------------------------------------------------     0
DmelDroj2       ------------------------------------------------------------     0
GmmJA1          ------------------------------------------------------------     0
ScalJA1         ------------------------------------------------------------     0
HsDnaJA1        ------------------------------------------------------------     0
HsDnaJA4        ------------------------------------------MARGGSQSWS--SGESDG     16
GmmJB15         LKSVSTSVLL---------QSFIDFIDCRKTSYV------HQFKTQTQTHYPDRQRETKR     49
GmmJD1          -------------------------------------------------MKNL-EGEIKR     10
EcDnaJ          ------------------------------------------------------------     0
HsDnaJA3        WLS--RKLSV---------PAFASSLTSCGPRAL------LTLRPGVSLTGTK-HNPFIC     79
GmmJA3          FLQVHRP--------------FAAFISLRRP--V------FLS---VDGIAPI-TTATAA     67
DmelTid56       --------CL----------AAAAFSTPRATSYR------ILS---SAGSGST-RADAPQ     51
ScalJA3         VSQQQQPHFL---------ATIAAFTKQC---QK------SPN---YEGGLAG-QQQLPQ     69

DmelAT19485     -------------------MPKDYYKILGIQRTANDGEIRKAYHKQALRYHPDKNK-SPQ     40
HsDnaJB13       -------------------MGQDYYSVLGITRNSEDAQIKQAYRRLALKHHPLKSN-EPS     40
HsDnaJB5        --VKFR-NKETSAG-PVAVMGKDYYKILGIPSGANEDEIKKAYRKMALKYHPDKNK-EPN     154
HsDnaJB1        -------------------MGKDYYQTLGLARGASDEEIKRAYRRQALRYHPDKNK-EPG     40
HsDnaJB4        -------------------MGKDYYCILGIEKGASDEDIKKAYRKQALKFHPDKNK-SPQ     40
GmmJB5          -------------------MGKDYYKTLGIPKTATDDEVKKAYRKLALRYHPDKNK-AAN     40
DmelCG5001      -------------------MGKDYYKILGLPKTATDDEIKKAYRKLALRYHPDKNK-AAN     40
ScalJB5         -------------------MGKDYYKTLGIPKTATDEEIKKAYRKLALRYHPDKNK-AAN     40
DmelDnaJ-1      -------------------MGKDFYKILGLERKASDDEIKKAYRKLALKYHPDKNK-SPQ     40
```

```
GmmJB1          --CVVK-VIWNLIT-HTSTMGKDFYEILGIGKNANDDEIKKAYRKLALKYHPDKNK-TPQ     73
GmmJB4          -------------------MSKNFYDILGISKNANDDEIKKAYRKLALKYHPDKNK-TPQ     40
ScalJB4         -------------------MGKDFYKILGINKGASDDEIKKAYRKLALKYHPDKNK-TPQ     40
HsDnaJB11       --STFC-LLLLYLI-GAVIAGRDFYKILGVPRSASIKDIKKAYRKLALQLHPDRNPDDPQ     62
GmmJB11         --IVIV-QIIVCLF-ETALAGRDFYKILNVRKGASTNEIKKAYRKLAKELHPDKNKHDPD     62
DmelCG4164      --LVII-QLSLLLV-EESFAGRDFYKILNVKKNANTNEVKKAYRRLAKELHPDKNKDDPD     62
ScalJB11        --VLFV-QITLCLL-NSAFAGRDFYQILNVKKSASTNDIKKAYRKLAKELHPDKNKDDPD     62
DmelDnaJ-H      ------------------MDNLNLYDVLKVAPDATDEEIKKNYRKLAKEFHPDKNP---D     39
GmmJA2          ------------------MENLDLYEILGVTKNSTDAEIKKNYRKLAKEFHPDKNP---N     39
ScalJA2         ------------------MEHLDLYEIIGVSKDATDAEIKKNYRKLAKEFHPDKNP---D     39
HsDnaJA2        ---------------MANVADTKLYDILGVPPGASENELKKAYRKLAKEYHPDKNP---N     42
DmelDroj2       -----------------MVKETGYYDILGVKPNATPDELKKAYRKLALKYHPDKNP---N     40
GmmJA1          -----------------MVKETGYYDLLGCKPNASAEELKKAYRKLALKYHPDKNP---N     40
ScalJA1         -----------------MVKETGYYDLLGVKPNATPEELKKAYRKLALKYHPDKNP---N     40
HsDnaJA1        -----------------MVKETTYYDVLGVKPNATQEELKKAYRKLALKYHPDKNP---N     40
HsDnaJA4        --QPKE-QTPEKPR-HKMVKETQYYDILGVKPSASPEEIKKAYRKLALKYHPDKNP---D     69
GmmJB15         MQENFEVSWWLIQTKRRICLKATITSFNTCPRDAITSQIKAAYYALATRYHPDESKRRQQ     109
GmmJD1          -TIEVD-------ENKD-VPMDHHYKLLGISKNATTMQIKAAYCTLAKKFHNYANSNAEN     61
EcDnaJ          ------------------MAKQDYYEILGVSKTAEEREIKKAYKRLAMKYHPDRNQGDKE     42
HsDnaJA3        -TASFH-------TSAP-LAKEDYYQILGVPRNASQKEIKKAYYQLAKKYHPDTNKDDPK     130
GmmJA3          -KRNFH-------SSHP-LNGKDYYQILGVAKNASSKEIKKAYYQLAKKYHPDTNKNDPD     118
DmelTid56       -VRRLH-------TTRD-LLAKDYYATLGVAKNANGKDIKKAYYQLAKKYHPDTNKEDPD     102
ScalJA3         -RRNFH-------TTNA-VKAKDYYQTLGVAKNASAKEIKKAYYQLAKKYHPDTNKEDPD     120
                                                 :   :::  *   * . *   .

DmelAT19485     --AEEIFKQVAKAYEVLSDKKKRGSYDSRNDKGTRRNTANQGSGFGDGTAFGSCGGGSGS     98
HsDnaJB13       --SAEIFRQIAEAYDVLSDPMKRGIYDKFGEEGLKGGIPL--------------EF--GS     82
HsDnaJB5        --AEEKFKEIAEAYDVLSDPKKRGLYDQYGEEGLKTGG------------------GTSG     194
HsDnaJB1        --AEEKFKEIAEAYDVLSDPRKREIFDRYGEEGLKGSGPS--------------GGSGGG     84
HsDnaJB4        --AEEKFKEVAEAYEVLSDPKKREIYDQFGEEGLKGGA------------------GGTD     80
GmmJB5          --AEEKFKEVAEAYEVLTDKNKREIYDKYGEEGLKSGGVR--------------NG--G-     81
DmelCG5001      --AEDKFKEVAEAYEVLSDKSKREVYDKYGEDGLKSGGTR--------------NG--G-     81
ScalJB5         --AEEKFKEVAEAYEVLSDKNKREVYDKYGEDGLKSGGRS--------------NG--T-     81
DmelDnaJ-1      --AEERFKEIAEAYEVLSDKKKRDIFDNYGEDGLKGGQPG--------------PD--GG     82
GmmJB1          --AEERFKQIAEAYEVLSDKRKRDIYDQFGEEGLKGGVPG--------------TT--GK     115
GmmJB4          --AEERFKQIAEAYEVLSDQRKRDIYDQYGEQGLKDGISG--------------ST--GE     82
ScalJB4         --AEERFKEIAEAYEVLSDKKKRDIFDQYGEEGLKGGIPG--------------SG--GG     82
HsDnaJB11       --AQEKFQDLGAAYEVLSDSEKRKQYDTYGEEGLKD-GH---------------------     98
GmmJB11         --ASVKFQDLGAAYEVLSDPDKRKTYDRCGEECVKKDGA---------------------     99
DmelCG4164      --ASTKFQDLGAAYEVLSNPDKRKTYDRCGEECLKKEGM---------------------     99
ScalJB11        --ASTKFQDLGAAYEVLSDPDKRKTYDRCGEECLKKDGM---------------------     99
DmelDnaJ-H      --AGDKFKEISFAYEVLSDPEKRRIYDRYGLKGLQEGAE-------------------G-     77
GmmJA2          --AGDKFKEISFAYEVLSDPEKRKVYDRHGIKGLQEGAD-------------------G-     77
ScalJA2         --AGDKFKEISFAYEVLSDPEKRKIYDRYGIKGLQEGAD-------------------G-     77
HsDnaJA2        --AGDKFKEISFAYEVLSNPEKRELYDRYGEQGLREGSGG----------------GGG-     83
DmelDroj2       --EGEKFKAISQAYEVLSDADKRQVYDEGGEAAIKKGGAD----------------SGD-     81
GmmJA1          --EGEKFKAISQAYEVLSDPEKRSIYDEGGEAAIKKGGAD----------------TGD-     81
ScalJA1         --EGDKFKAISQAYEVLSDPDKRSIYDEGGEAAIKKGGAD----------------TGD-     81
HsDnaJA1        --EGEKFKQISQAYEVLSDAKKRELYDKGGEQAIKEGGAG----------------G-G-     80
HsDnaJA4        --EGEKFKLISQAYEVLSDPKKRDVYDQGGEQAIKEGGSG----------------SPS-     110
GmmJB15         VLISIRFQDILNAYRCLFDEARRHEYDTLQDGNSKTPKNER-------------------     150
GmmJD1          --ESQRFRDIVKAYHCLVNNVKNHEYKLDAKHMNIEEKQNQ-------------------     100
EcDnaJ          --AEAKFKEIKEAYEVLTDSQKRAAYDQYGHAAFEQGGMGGG--------------GFGG     86
HsDnaJA3        --AKEKFSQLAEAYEVLSDEVKRKQYDAYGSAGFDPGASG--------------------     168
GmmJA3          --ASRKFQEVSEAYEVLSDDQKRREFDTYGQTAEEMGRNGG------------------A     158
DmelTid56       --AGRKFQEVSEAYEVLSDEQKRREYDTYGQTAENIGRQGGG--------------FPGG     146
ScalJA3         --ASRKFQEVSEAYEVLSDDTKRREYDTYGQTTENMNRAGGG--------------F---     161
                      *  :  **  * :  :.  :.

DmelAT19485     GSGGGSGGGRQNNPRANFGRFFDNSESYSTFFEDIEND---------FDSDDDVL-----     144
HsDnaJB13       QTPWTTGYVFHGKPEKVFHEFFGGNNPFSEFFDAEGSE-------------VDL------     123
HsDnaJB5        GSSGSFHYTFHGDPHATFASFFGGSNPFDIFFASSRSTRPFSGFDP-DDMDVDEDEDPFG     253
HsDnaJB1        ANGTSFSYTFHGDPHAMFAEFFGGRNPFDTFFGQRNGE------EG-MDIDDPFSGFPMG     137
HsDnaJB4        GQGGTFRYTFHGDPHATFAAFFGGSNPFEIFFGRRMGG------GR-DSEEMEIDGDPFS     133
GmmJB5          NTNGTFTYQFHGDPRATFEQFFGSSNPFASFFDMSDN--LFDKNVFDLDTEHDFFASPFA     139
DmelCG5001      PSSNSFTYQFHGDPRATFAQFFGNSNPFASFFDMGDN--LFDKKVFDLDTEPDFFSSPFG     139
ScalJB5         SSNNTFTYQFHGDPRATFAQFFGSSNPFASFFDMSDN--LFDKNVFDLDTEHDFFSSPFG     139
DmelDnaJ-1      GQPGAYTYQFHGDPRATFAQFFGSSDPFGAFFTGGDN--MFSGGQG-GNT-NEIF-----     133
GmmJB1          ---SNSSYTYHGDPRATFAQFFGNANPFGIFFGAEDFGRMFEQPTIFMTA-DDLF-----     166
GmmJB4          ---PVPNYTYHGDARATFAQFFGNSNPFGGFFGTDDP--MFGQQTIFMSD-DDLY-----     131
ScalJB4         MDGGNGGYSYHGDPRATFAQFFGNADPFGIFFGSGDPTRIFESQTMFMGD-DDMY-----     136
HsDnaJB11       --------------------QSSHGDIFSHFFGDFGF-----------------------     115
GmmJB11         ---------------------DNNADPFASFFGDFGF-----------------------     115
DmelCG4164      --------------------MDHGGDPFSSFFGDFGF-----------------------     116
ScalJB11        --------------------MDHGHDPFGSFFGDFAF-----------------------     116
DmelDnaJ-H      --------------------FSDASEFFAQWFPFDRV-----------------------     94
GmmJA2          --------------------FSDAGEFFAQWFPFSNL-----------------------     94
```

```
ScalJA2         --------------------FSEGNEFFSQWFPFTSM-----------------------     94
HsDnaJA2        --------------------M---DDIFSHIFGGGLF-----------------------     97
DmelDroj2       --------------------FRNPMDFFEKFFGAGFG-----------------------     98
GmmJA1          --------------------FRNPMDFFEKFFGGGFS-----------------------     98
ScalJA1         --------------------YRNPMEFFEKFFGGGFS-----------------------     98
HsDnaJA1        --------------------FGSPMDIFDMFFGGG-------------------------     95
HsDnaJA4        --------------------FSSPMDIFDMFFGGG-------------------------     125
GmmJB15         ---MNTPR-------------------HRKMV----------------------------     160
GmmJD1          ---VTTSSSKSQ--VKLVPSLLDPPTKFINRR----KS----------------------     129
EcDnaJ          GA------DFSD--I---------FGDVFGDIFGGGRGR---------------------     108
HsDnaJA3        --------SQHS--YWKGGPTVDPEELFRKIFGE--FS----------------------     194
GmmJA3          GFAGHGPQGFSQ--SWQFRSTIDPEELFRKIFGDHNFS----------------------     194
DmelTid56       GAGGFGPEGFSQ--SWQFRSSIDPEELFRKIFGEGNFR----------------------     182
ScalJA3         --GGHGPQGFSQ--NWQFRSTIDPEELFRKIFGDGNFR----------------------     195
                                           .

DmelAT19485     -------LGGGAGAPKRR------------CEQQSPQSSIEHVIYVALEDIANGCNRR-M     184
HsDnaJB13       ------NFGGLQG-----------------RGVKKQDPQVERDLYLSLEDLFFGCTKK-I     159
HsDnaJB5        AFGR-FGFNGLSRGPRR-------APEPLYPRRKVQDPPVVHELRVSLEEIYHGSTKR-M     304
HsDnaJB1        MGG----FTNVNF--GR-------SRSAQEPARKKQDPPVTHDLRVSLEEIYSGCTKK-M     183
HsDnaJB4        AFG----FSMNGY--PR-------DRNSVGPSRLKQDPPVIHELRVSLEEIYSGCTKR-M     179
GmmJB5          GLGPRQGLGGA----FRSHSF---NVHTPFKKEKPQDPPVEHDLYVMLEEIYHGCVKK-M     191
DmelCG5001      GIGSRHGLGSGFRPSFRSHSF---NVHTPFKKEQKQDPPVEHDLYVTLEEIYHGCVKK-M     195
ScalJB5         GLGSRHGLGGAFRPSFRSHSF---NVHTPFKKEKQQDPPVEHDLYVTLEEIYHGCVKK-M     195
DmelDnaJ-1      -----WNIGGD-----DMFAF---NAQAP-SRKRQQDPPIEHDLFVSLEEVDKGCIKK-M     178
GmmJB1          -----AQM-GSSGGRFRSRSP---NRKQN-RRGQQQDPPIEYDLYVSLEEIDKGCVKN-M     215
GmmJB4          -----TGMTGGPGGAFRSHSF---NAQPN-RKRQQQDPPIEHDLYVSLEEVDKGCVKK-M     181
ScalJB4         -----M--GGGPGGAFRSQSF---NAQPS-RKRSQQDPPIEHDLFVSLEEIDKGCVKK-M     184
HsDnaJB11       ------MFGGTPR---QQ------------DRNIPRGSDIIVDLEVTLEEVYAGNFVE-V     153
GmmJB11         ------HFGNGDH---QQ-------------HDTPKGANIVMNMYVTLEELYSGNFVE-I     152
DmelCG4164      ------HFGGD-G---QQ-------------QDAPRGADIVMDLYVSLEELYSGNFVE-I     152
ScalJB11        ------HFGGGDP---HQ-------------HDTPKGANIVMNMFVTLEELYSGNFVE-I     153
DmelDnaJ-H      ------SSEGR----------------------GRRNGKVVVKVELTLEEIYVGGMKKKV     126
GmmJA2          ------G-GHSR-------------------EGRGKVGQIVIKLEVTLEEIYNGNIAKTI     128
ScalJA2         ------GGGHGR-------------------DARGKGAQIVIKLEVTLEEIYNGNVSKTV     129
HsDnaJA2        ------GFMGNQS---RS------------RNGRRRGEDMMHPLKVSLEDLYNGKTTK-L     135
DmelDroj2       ------GSGGGR-------------------RRERRGKDVVHQMSVQLEELYNGATRK-L     132
GmmJA1          ---------SGR-------------------RRERRGKDVVHQMSVTLEELYNGATRK-L     129
ScalJA1         ---------SGR-------------------RRERRGKDVVHQLSVQLEELYNGATRK-L     129
HsDnaJA1        ---------GRM-------------------QRERRGKNVVHQLSVTLEDLYNGATRK-L     126
HsDnaJA4        ---------GRM-------------------ARERRGKNVVHQLSVTLEDLYNGVTKK-L     156
GmmJB15         -----------------------------------K--------------SAAQLTKQ-L     170
GmmJD1          ----------------PNSAKDTLQTVAIKEDYLKMANTATNLELTFLEAAHGQKKS-V     171
EcDnaJ          ----------------Q--------------RAARGADLRYNMELTLEEAVRGVTKE-I     136
HsDnaJA3        ----------------SSSFGDF--------QTVFDQPQEYFMELTFNQAAKGVNKE-F     228
GmmJA3          ----------------PNNFEDFA-----ESQFGFGRAQEIVMDLSFAQAARGVNKD-V     231
DmelTid56       ----------------TNSFDDFA-----DSKFGFGQAQEMVMDLTFAQAARGVNKD-V     219
ScalJA3         ----------------SNAFDDFA-----ESQYGFGRSQEVVMDLSFAQAARGVNKD-I     232
                                                                           .

DmelAT19485     -----------------------------------KISRA--------------------     189
HsDnaJB13       -----------------------------------KISRR--------------------     164
HsDnaJB5        -----------------------------------KITRR--------------------     309
HsDnaJB1        -----------------------------------KISHK--------------------     188
HsDnaJB4        -----------------------------------KISRK--------------------     184
GmmJB5          -----------------------------------KISRR--------------------     196
DmelCG5001      -----------------------------------KISRR--------------------     200
ScalJB5         -----------------------------------KISRR--------------------     200
DmelDnaJ-1      -----------------------------------KISRM--------------------     183
GmmJB1          -----------------------------------KITRM--------------------     220
GmmJB4          -----------------------------------KISRM--------------------     186
ScalJB4         -----------------------------------KISRM--------------------     189
HsDnaJB11       VRNKPVARQA--------P---GKRKCN-CR---QEMRTTQLGPGRFQMTQEV-------     191
GmmJB11         VRNKPVLKPA--------S---GTRKCN-CR---QEMVTRNLGPGRFQMIQQT-------     190
DmelCG4164      VRNKPVTKPA--------S---GTRKCN-CR---QEMVTRNLGPGRFQMIQQT-------     190
ScalJB11        VRNKPVLKPA--------A---GTRKCN-CR---QEMVTRNLGPGRFQMMQQL-------     191
DmelDnaJ-H      EYNRQKLCSKCNGDGGPKE---AHESCETCGGAGRAAAFTFMGL---SPFDTTCPTCDGR     180
GmmJA2          EYKRTSFCSECHGDGGPKE---AQQKCTQCNGVGRMASYAFMGL---TAVETICSVCHGR     182
ScalJA2         EYKRTSFCSQCGGDGGPKE---AQEKCSHCNGMGRKASYAFMGL---TAVETICSACHGH     183
HsDnaJA2        QLSKNVLCSACSGQGGKSG---AVQKCSACRGRGVRIMIRQLAPGMVQQMQSVCSDCNGE     192
DmelDroj2       QLQKNVICDKCEGRGGKKG---SIEKCLQCRGNGVETRVQQIAPGIMQHIEQVCRKCSGT     189
GmmJA1          ALQKNVICDKCEGRGGKKG---ATEKCTQCHGAGLETRIQQIGPGLVHHVEKVCSRCSGT     186
ScalJA1         ALQKNVICDKCEGRGGKKG---AIEKCMQCQGAGLESRMQQIGPGLVQHIEQVCRKCSGT     186
HsDnaJA1        ALQKNVICDKCEGRGGKKG---AVECCPNCRGTGMQIRIHQIGPGMVQQIQSVCMECQGH     183
HsDnaJA4        ALQKNVICEKCEGVGGKKG---SVEKCPLCKGRGMQIHIQQIGPGMVQQIQTVCIECKGQ     213
GmmJB15         IS--------------KPI----------------PTTTL--GN---FLSAANK------     189
GmmJD1          EMKYLKKCPSCNGKSHRAARLLGPEQCRKCKGTGQILKKS--VS---FSSMVVCDQCNGK     226
```

```
EcDnaJ            RIPTLEECDVCHGSGAKPG--TQPQTCPTCHGSGQVQMRQ--GF---FAVQQTCPHCQGR      189
HsDnaJA3          TVNIMDTCERCNGKGNEPG--TKVQHCHYCGGSGMETINT--GP---FVMRSTCRRCGGR      281
GmmJA3            NVNIVDTCPKCKGTKCEPG--TKPGRCQYCNGTGMETIST--GP---FVMRSTCRYCQGT      284
DmelTid56         NVNVVDQCPKCAGTKCEPG--TKPGRCQYCNGTGFETVST--GP---FVMRSTCRYCQGT      272
ScalJA3           NVNVVDTCPKCNGTKCEPG--TKPGRCQYCNGTGMETIST--GP---FVMRSTCRYCQGT      285


DmelAT19485       ----------SGRNGVDGVQYDRILTVKIPPGCKAGTKICFPNEGIQLPNLEPA-NVVFI      238
HsDnaJB13         ---------VLNEDGYSSTIKDKILTIDVKPGWRQGTRITFEKEGDQGPNIIPA-DIIFI      214
HsDnaJB5          ---------RLNPDGRTVRTEDKILHIVIKRGWKEGTKITFPKEGDATPDNIPA-DIVFV      359
HsDnaJB1          ---------RLNPDGKSIRNEDKILTIEVKKGWKEGTKITFPKEGDQTSNNIPA-DIVFV      238
HsDnaJB4          ---------RLNADGRSYRSEDKILTIEIKKGWKEGTKITFPREGDETPNSIPA-DIVFI      234
GmmJB5            ---------VQLPDGTS-KKEDKYVSISIKPGWKSGTKVTFQKEGDQIPGRIPA-DIVFI      245
DmelCG5001        ---------IVQADGSS-RKEEKFLAISIKPGWKSGTKVTFQKEGDQAPGKIPA-DIVFI      249
ScalJB5           ---------VQQPDGSS-KKEDKYVSISIKPGWKSGTKVTFQKEGDQSPGKIPS-DIVFI      249
DmelDnaJ-1        ----------ATGSNGP-YKEEKVLRITVKPGWKAGTKITFPQEGDSAPNKTPA-DIVFI      231
GmmJB1            ----------SMATGRP-RKDEKILNINVKPGWKSGTKITFQKEGDEIPGKIPA-DIVFV      268
GmmJB4            ----------SMATGQS-RKEEKVLNITVKPGWKSGTKITFQREGDQTPGKIPA-DIIFI      234
ScalJB4           ----------SMATGQA-RKEEKVLNITVKAGWKAGTKITFQQEGDQTPGKIPA-DIIFI      237
HsDnaJB11         --------VCDECPNVKLVNEERTLEVEIEPGVRDGMEYPFIGEGEPHVDGEPG-DLRFR      242
GmmJB11           --------VCDECPNVMLVNEERTLEIEIEAGMIDGQETRFVAEGEPHMDGDPG-DLIIK      241
DmelCG4164        --------VCDECPNVKLVNEERTLEIEVEQGMVDGQETRFVAEGEPHIDGEPG-DLIVR      241
ScalJB11          --------VCDECPNVKLVNEERTLEIEVEPGMIDGQETRFVAEGEPHMDGDPG-DLMVK      242
DmelDnaJ-H        GFTIRDDKKCSPCQGSGFVEQKMKRDLVVERGAPHMLKVPFANEGHQMRGGEFG-DLIVV      239
GmmJA2            GNVIAENLQCGTCHGNGLVEELAKREITVEKGAPHMLKIPFPSEGHQGLQGSRG-DLIVV      241
ScalJA2           GNIIPEDLRCTSCQGKGLVEENVKREIVIERGAPNMLKIPFFSEGNQGLQGFRG-DMVVV      242
HsDnaJA2          GEVINEKDRCKKCEGKKVIKEVKILEVHVDKGMKHGQRITFTGEADQAPGVEPG-DIVLL      251
DmelDroj2         GETIQEKDRCKNCSGRKTVRERKVLEVHIEKGMRDGQKIVFTGEGDHEPESQPG-DIIIL      248
GmmJA1            GETINEKDRCKQCSGRKTVRERKVLEVHIEKGMRDGQKIVFSGEGDHEPDSQPG-DIVIL      245
ScalJA1           GETISEKDRCKQCHGRKTVRERKVLEVHIEKGMRDGQKIMFSGEGDHEPESQPG-DIIIL      245
HsDnaJA1          GERISPKDRCKSCNGRKIVREKKILEVHIDKGMKDGQKITFHGEGDQEPGLEPG-DIIIV      242
HsDnaJA4          GERINPKDRCESCSGAKVIREKKIIEVHVEKGMKDGQKILFHGEGDQEPELEPG-DVIIV      272
GmmJB15           ---------------------------EDTNGTKSGDIV---------------------      201
GmmJD1            RYINR--NQCDMCDNRGFVLDAAKVVIPIPLGAKTGDKVSVKNPHT-------GQCTNYC      277
EcDnaJ            GTLIK--DPCNKCHGHGRVERSKTLSVKIPAGVDTGDRIRLAGEGEAGEHGAPAGDLYVQ      247
HsDnaJA3          GSIII--SPCVVCRGAGQAKQKKRVMIPVPAGVEDGQTVRMPV---------GKREIFIT      330
GmmJA3            RQYVK--YPCAECEGKGQTVQRKKVTVPVPAGIEDGQTVRMQV---------GKKELFVT      333
DmelTid56         RQHIK--YPCSECEGKGRTVQRRKVTVPVPAGIENGQTVRMQV---------GSKELFVT      321
ScalJA3           RQYIK--YPCAECEGKGQTVQRKKVTVPVPAGIENGQTVRMQV---------GKKELFIT      334
                                                 *

DmelAT19485       IRDKPHPIFRRDGNNLLYT-AEISLKDALCGLHVMVPTLLGRPMELKTDVGEVISPKSVR      297
HsDnaJB13         VKEKLHPRFRRENDNLFFV-NPIPLGKALTCCTVEVRTLDDRLLNIP--INDIIHPKYFK      271
HsDnaJB5          LKDKPHAHFRRDGTNVLYS-ALISLKEALCGCTVNIPTIDGRVIPLP--CNDVIKPGTVK      416
HsDnaJB1          LKDKPHNIFKRDGSDVIYP-ARISLREALCGCTVNVPTLDGRTIPVV--FKDVIRPGMRR      295
HsDnaJB4          IKDKDHPKFKRDGSNIIYT-AKISLREALCGCSINVPTLDGRNIPMS--VNDIVKPGMRR      291
GmmJB5            IRDKPHAMFKREGSDLRYT-ARLTLKQV--------------------------------      272
DmelCG5001        IRDKPHAMFKREGSDLRYT-ARLTLKQALCGVVFQVPTMSGDKLRIS-TMQEIIKPNTVK      307
ScalJB5           IRDKPHAMFKREGSDLRYT-ARLTLKQALCGVVFQVPTMSGDKLRIS-TMQEIIKPNTVK      307
DmelDnaJ-1        IRDKPHSLFKREGIDLKYT-AQISLKQALCGALVSVPTLQGSRIQVN-PNHEIIKPTTTR      289
GmmJB1            IKDRPHPIFKREGTDFRYT-AKISLKQALCGTTVKVPTLQGDKITLS-LYNEVITPNTVK      326
GmmJB4            IRDKPHPLFKREGTDIRYN-AKINLKQALCGTSVQVPTLQGDRITVN-TQGEVIKPNTVK      292
ScalJB4           IRDKPHPLFKREGSDLRYT-AKISLKQALCGTSISVPTLHGDRITVS-SQGEILKPTTVK      295
HsDnaJB11         IKVVKHPIFERRGDDLYTN-VTISLVESLVGFEMDITHLDGHKVHISR--DKITRPGAKL      299
GmmJB11           ILQTPHQRFHRKGDDLYTN-VTISLQDALVGFTMDVTHLDGHKVSVTR--EKITWPGARI      298
DmelCG4164        VQQMPHPRFLRKNDDLYTN-VTISLQDALVGFSMEIKHLDGHLVPVTR--EKVTWPGARI      298
ScalJB11          VMQTPHERFQRKEDDLYTN-VTISLQDALIGFSMDIIHLDGHKVTVTR--EKITWPGARI      299
DmelDnaJ-H        ISQMEHPIFQRRHANLYMRDLEINITEALCGYSHCFKHLDGRNVCLRTYPGEVLQHNQIK      299
GmmJA2            LIQTEHTLFQRQHNDLIMRNIHVNLTQALCGFVHCFKHLDGRQICVATRPGEVIRHQELK      301
ScalJA2           LIQAEHPTFERRNNDLLMRNININITQALCGFVHCFKHLDGRNICISTNPGEVIRHGEFK      302
HsDnaJA2          LQEKEHEVFQRDGNDLHMT-YKIGLVEALCGFQFTFKHLDGRQIVVKYPPGKVIEPGCVR      310
DmelDroj2         LDEKEHSTFAHAGQDLMMK-MPLQLVEALCGFQRIVKTLDDRDLIVSTQPGEVIRHEMTK      307
GmmJA1            LDEKEHHTFVHAGTDLMMK-MPIQLVEALCGFQRVIKTLDERDLVITSPKGEVMKHEMTK      304
ScalJA1           LDEKEHQTFVHAGSDLMMK-MPIQLVEALCGFNRIIKTLDDRDLVFSSAPGEVIRHEMTK      304
HsDnaJA1          LDQKDHAVFTRRGEDLFMC-MDIQLVEALCGFQKPISTLDNRTIVITSHPGQIVKHGDIK      301
HsDnaJA4          LDQKDHSVFQRRGHDLIMK-MKIQLSEALCGFKKTIKTLDNRILVITSKAGEVIKHGDLR      331
GmmJB15           --SVKSDYYPRVEYNIFID-KYITISVAILDGTFNVRGIYETLDLKV-ELDT--ESHSQF      255
GmmJD1            IQVKCSNYFTREGNNVLTD-KYITISEAVLGGTFKVRGVYENVNLKI-APGT--ESHSKF      333
EcDnaJ            VQVKQHPIFEREGNNLYCE-VPINFAMAALGGEIEVPTLDGRVKLKV-PGET--QTGKLF      303
HsDnaJA3          FRVQKSPVFRRDGADIHSD-LFISIAQALLGGTARAQGLYETINVTI-PPGT--QTDQKI      386
GmmJA3            FRVEPSRHFKRKGADVHSS-ATISLAQAVLGGTVRIEGVYEDEWLNI-EPGT--SSHHKI      389
DmelTid56         FRVERSDYFRREGADVHTD-AAISLAQAVLGGTVRVQGVYEDQWINV-EPGT--SSHHKI      377
ScalJA3           FRVEPSRYFRRDGADVHTD-AVISLSQAALGGTIRVEGVYEDQWLNI-EPGT--SSHHKI      390
                          : :   :.      : :
```

```
DmelAT19485     RILGYGLPDSINNSRRGSIVVRFSIQFPDAIS--KELASSLDR-----------------     338
HsDnaJB13       KVPGEGMPLPEDPTKKGDLFIFFDIQFPTRLT--PQKKQMLRQ-----------------     312
HsDnaJB5        RLRGEGLPFPKVPTQRGDLIVEFKVRFPDRLT--PQTRQILKQ-----------------     457
HsDnaJB1        KVPGEGLPLPKTPEKRGDLIIEFEVIFPERIP--QTSRTVLEQ-----------------     336
HsDnaJB4        RIIGYGLPFPKNPDQRGDLLIEFEVSFPDTIS--SSSKEVLRK-----------------     332
GmmJB5          ------------------------------------------------------------     272
DmelCG5001      RIQGYGLPFPKDTTRKGDLLVAFDIQFPEKLT--AAQKEVLKD-----------------     348
ScalJB5         RIQGYGLPFPKDTSRKGDLLVAFDIQFPEKLT--AAQKELLRD-----------------     348
DmelDnaJ-1      RINGLGLPVPKEPSRRGDLIVSFDIKFPDTLA--PSLQNQLSE-----------------     330
GmmJB1          RINGRGLPFHKEPSRRGDLLVSFDIKFPNKIH--RSLKDILAE-----------------     367
GmmJB4          RINNRGLPFPKEPSRRGDLLVSFDIKFPETLQ--PPVKELLDE-----------------     333
ScalJB4         RLSGRGLPFPKEPSKRGDLLVSYDIKFPDSLQ--PGTKELISE-----------------     336
HsDnaJB11       WKKGEGLPNFDNNNIKGSLIITFDVDFPKEQLTEEAREGIKQLLKQGSVQKVYNGLQGY-     358
GmmJB11         VKKGEGMPNYENNNLHGNLHITFDVDFPKKSLNEVEKERTNQKHLRTKLSESYLQWTVKT     358
DmelCG4164      RKKGEGMPNFENNNLTGNLYITFDVEFPKKDLTEEDKEALKKILDQSSINRIYNGL----     354
ScalJB11        RKKGEGMPNYENNNLHGNLYITFDVEFPKKDLDDTEKEDLKKILDQASINRVYNGL----     355
DmelDnaJ-H      MVRGSGMPVFNKATDSGDLYMKFKVKFPDNDFATAPQLAMLED-----------------     342
GmmJA2          MIPGEGMPLRNNPFDRGDLLIHFMVDFPENGFATSEQLEMLET-----------------     344
ScalJA2         VVPVEGMPLRNNPFDRGDLIVQFSIQFPENHFATPEQLAQLET-----------------     345
HsDnaJA2        VVRGEGMPQYRNPFEKGDLYIKFDVQFPENNWINPDKLSELED-----------------     353
DmelDroj2       CIAEEGMPIFKNPMEKGTLIIQFEVIFPEVIN--PSVVPTLKQ-----------------     348
GmmJA1          CVMDEGMPLYKNPLEKGRLIIQFEVIFPESTP--LSVISALEQ-----------------     345
ScalJA1         CIMDEGMPLYKNPLEKGRLIIQFEVIFPDSIP--VSVIPALEQ-----------------     345
HsDnaJA1        CVLNEGMPIYRRPYEKGRLIIEFKVNFPENGFLSPDKLSLLEK-----------------     344
HsDnaJA4        CVRDEGMPIYKAPLEKGILIIQFLVIFPEKHWLSLEKLPQLEA-----------------     374
GmmJB15         KSADKDIKT-R--DAVGDHIATIKVQTSRQ--LNVKRMQLILALAKTEHE-KWFVIICVV     309
GmmJD1          VLKGKGIRS-R--EGPGDHIITVKIKIPRQ--LTIKQRQLMLTFAKTEES-DYQYID---     384
EcDnaJ          RMRGKGVKS-VRGGAQGDLLCRVVVETPVG--LNEKQKQLLQELQESFGG-PTGEHNSPR     359
HsDnaJA3        RMGGKGIPR-INSYGYGDHYIHIKIRVPKR--LTSRQQSLILSYAEDETD-VEGTVNGVT     442
GmmJA3          LLRNKGLKR-VNAHGHGDHYVTIKIEMPKH--LNKEQKELLIKYAELETN-TPGSIHGIT     445
DmelTid56       MLRGKGLKR-VNAHGHGDHYVHVKITVPSAKKLDKKRLALIEAYAELEED-TPGQIHGIA     435
ScalJA3         FLKGKGLKR-VNAHGHGDHYVNVKIEIPKT--LTKEQKELLQRFAELETN-TPGSVHGIT     446

DmelAT19485     -LLQN-------------------------------------------------------     342
HsDnaJB13       -ALLT-------------------------------------------------------     316
HsDnaJB5        -HLPCS------------------------------------------------------     462
HsDnaJB1        -VLPI-------------------------------------------------------     340
HsDnaJB4        -HLPAS------------------------------------------------------     337
GmmJB5          ------------------------------------------------------------     272
DmelCG5001      -ML---------------------------------------------------------     350
ScalJB5         -ML---------------------------------------------------------     350
DmelDnaJ-1      -LLPN-------------------------------------------------------     334
GmmJB1          -MLPNSE-----------------------------------------------------     373
GmmJB4          -ILPN-------------------------------------------------------     337
ScalJB4         -ILPN-------------------------------------------------------     340
HsDnaJB11       ------------------------------------------------------------     358
GmmJB11         LTLPIREGL---------------------------------------------------     367
DmelCG4164      ------------------------------------------------------------     354
ScalJB11        ------------------------------------------------------------     355
DmelDnaJ-H      -LLPPRQPIVIP-KNAEEVQ------MTDYKPQPRQQ---E------------DEDG-QS     378
GmmJA2          -LLPPREPFTMP-EEAEEVL------LVDVQPRAEDS---RGAHGGG-HE-DDDEFD-GN     390
ScalJA2         -LLPPREPFVMP-PEAEEVQ------LVEFQPRDEHD---TRGAHGG-MD-DDEEYE-GG     391
HsDnaJA2        -LLPSRPEVPNIIGETEEVE------LQEFDSTRGSG---GGQ-RRE-AYNDSSDEESSS     401
DmelDroj2       -CLPPAPEVDIP-IDAEQTV------LEDFDPKQRRQ-----QHQRM-AYDEDDGG--YQ     392
GmmJA1          -CLPPRPEVTIP-IDVEQVT------LSDFDPKQRRE----QQHHRM-VYEEDDRYE-QA     391
ScalJA1         -CLPPRPEVEIP-IDAEQVT------LSDFDPKHRGQ---QQQHHRM-PYDEDDRYD-DG     392
HsDnaJA1        -LLPERKEVEET-DEMDQVE------LVDFDPNQERR---RHY-NGE-AYEDDEHHP-RG     390
HsDnaJA4        -LLPPRQKVRIT-DDMDQVE------LKEFCPNEQNW---R-Q-HRE-AYEEDEDGP-QA     419
GmmJB15         NCLEN-------------------------------------------------------     314
GmmJD1          ------------------------------------------------------------     384
EcDnaJ          --------------------------------------------------------S--K     361
HsDnaJA3        LTSSGGSTMDSS-AG-------------------------------SKARREAGEDE--E     468
GmmJA3          KRKDGSSKATTF-ASMGNDNSTQFSSAEEV------------FNKTETKQDDDDTKD--K     490
DmelTid56       NRKDGSKQATSE------------------------------------------------     447
ScalJA3         KRTDGSKKATST-SSTNEQNKSSEASTEKFSENSASQKTSTSTSNSSAKSEEGDKKD--S     503

DmelAT19485     ---------------     342
HsDnaJB13       ---------------     316
HsDnaJB5        ---------------     462
HsDnaJB1        ---------------     340
HsDnaJB4        ---------------     337
GmmJB5          ---------------     272
DmelCG5001      ---------------     350
ScalJB5         ---------------     350
DmelDnaJ-1      ---------------     334
```

GmmJB1	---------------	373
GmmJB4	---------------	337
ScalJB4	---------------	340
HsDnaJB11	---------------	358
GmmJB11	---------------	367
DmelCG4164	---------------	354
ScalJB11	---------------	355
DmelDnaJ-H	SHFEGVQCQTA----	389
GmmJA2	THFERVQCQTG----	401
ScalJA2	PHFERVQCQTA----	402
HsDnaJA2	HHGPGVQCAHQ----	412
DmelDroj2	DGPRVQQCTSS----	403
GmmJA1	--PRVQQCTSS----	400
ScalJA1	PGPRMQQCSSS----	403
HsDnaJA1	----GVQCQTS----	397
HsDnaJA4	----GVQCQTA----	426
GmmJB15	---------------	314
GmmJD1	---------------	384
EcDnaJ	SFFDGVKKFFDDLTR	376
HsDnaJA3	GFLSKLKKMFTS---	480
GmmJA3	GLLSKLKSIFN----	501
DmelTid56	---------------	447
ScalJA3	GFLSKLKSMFN----	514
