# Supplementary figures and images for: Hsp70/J-protein machinery from Glossina morsitans morsitans, vector of African trypanosomiasis
Source: PLoS One. 2017 Sep 13;12(9):e0183858. doi: 10.1371/journal.pone.0183858 (PMC5597180; doi:10.1371/journal.pone.0183858)

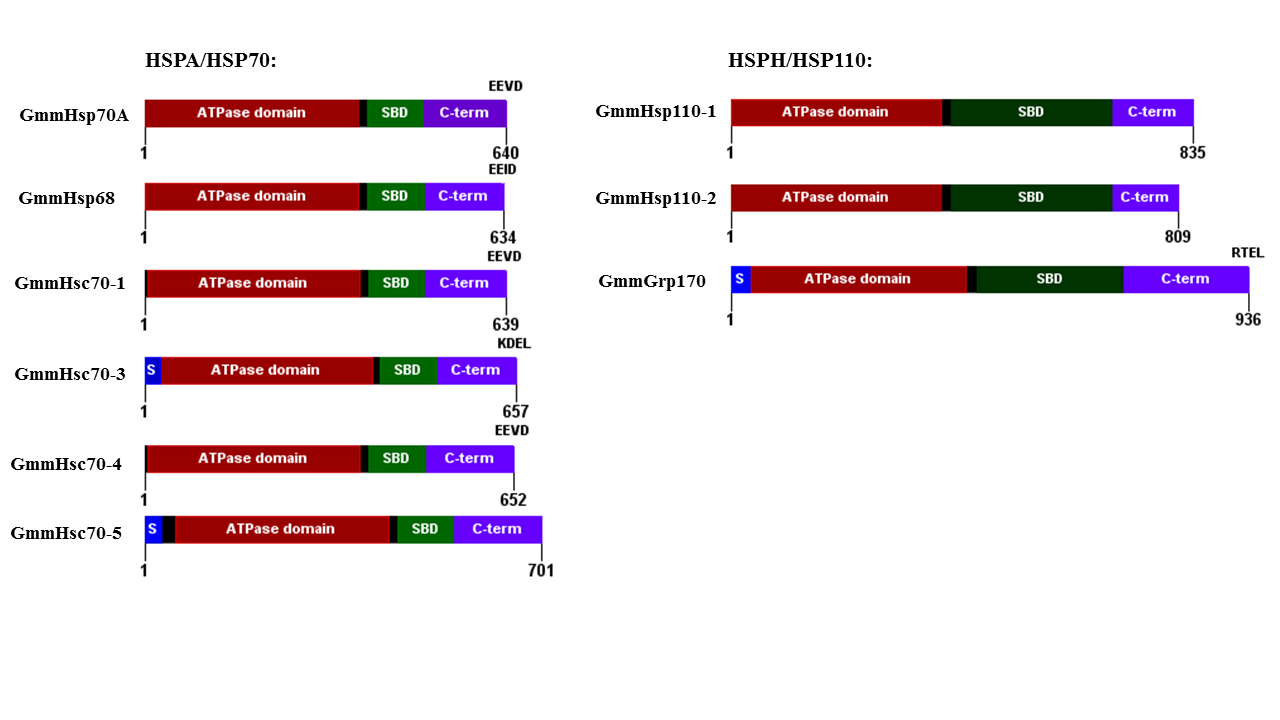

Supplement: S4 Fig — Each protein sequence for the G. m. morsitans Hsp70 superfamily is represented by an open bar with the various protein domains and other associated features that were identified using Prosite [44] and SMART 7 [43] are displayed as colour blocks within the open bar. These domains and associated features include the N-terminal ATPase domain (red), substrate binding domain (SBD; green), putative substrate binding domain for NEFs (SBD; dark green), C-terminal region (C-terminal; purple) and targeting signal peptides (S; dark blue). (TIF) [file pone.0183858.s004.tif]
